# Biological implications of genetic variations in autism spectrum disorders from genomics studies

**DOI:** 10.1042/BSR20210593

**Published:** 2021-07-22

**Authors:** Yue Zhang, Xuanshi Liu, Ruolan Guo, Wenjian Xu, Qi Guo, Chanjuan Hao, Xin Ni, Wei Li

**Affiliations:** Beijing Key Laboratory for Genetics of Birth Defects, Beijing Pediatric Research Institute; MOE Key Laboratory of Major Diseases in Children; Rare Disease Center, National Center for Children’s Health, Beijing Children’s Hospital, Capital Medical University, Beijing, China

**Keywords:** Autism spectrum disorder, genetic basis, genomic structural variation, neurodevelopment, whole-genome sequencing

## Abstract

Autism spectrum disorder (ASD) is a highly heterogeneous neurodevelopmental condition characterized by atypical social interaction and communication together with repetitive behaviors and restricted interests. The prevalence of ASD has been increased these years. Compelling evidence has shown that genetic factors contribute largely to the development of ASD. However, knowledge about its genetic etiology and pathogenesis is limited. Broad applications of genomics studies have revealed the importance of gene mutations at protein-coding regions as well as the interrupted non-coding regions in the development of ASD. In this review, we summarize the current evidence for the known molecular genetic basis and possible pathological mechanisms as well as the risk genes and loci of ASD. Functional studies for the underlying mechanisms are also implicated. The understanding of the genetics and genomics of ASD is important for the genetic diagnosis and intervention for this condition.

## Introduction

Autism spectrum disorder (ASD) is defined as social interaction and communication deficits, restrictive repetitive behaviors across a phenotypic spectrum, with onset during early childhood [1]. Complications often occur including intellectual disability, epilepsy, motor deficits (hypotonia, apraxia or motor delay), gastrointestinal disturbances, and sleep abnormalities. ASD is a common neurodevelopmental disorder occurring in approx. 1% of individuals worldwide. The affected number has been increased rapidly in these years. According to the Centers for Disease Control and Prevention (U.S.A.), the prevalence of ASD is approx. 1:54, with a significantly higher proportion of males affected compared with females [2].

ASD is a complex and highly inheritable disease. Its clinical presentation is highly heterogeneous by encompassing a wide range of cognitive and adaptive abilities. The degree of heritability of ASD has been estimated as 40–90% [3,4], but a significant proportion of genetic risk factors remains undefined.

As the technology of molecular genetics and genomics developed rapidly, inherited risk factors of ASD have been identified through big genomics data in large patient cohort studies. Functional experiments are conducted to explain how these genomic variations play a role in molecular, cellular, or brain functions to uncover the potential pathogenic mechanisms. In this review, we highlight the recent findings of molecular genetics and genomics of ASD, shading light on how genetic risk factors affect cellular functions and clinical phenotypes, to direct the precision diagnosis and intervention of ASD.

## Genetic basis of ASD

Genetic or environmental risk factors of prenatal, perinatal or postnatal period, could cause ASD alone or together [5]. Environmental factors such as exposure to heavy metal, deficiency of vitamin D, advanced parental age, and complications of pregnancy or birth have been shown to be risk factors of ASD [5–7]. According to the twin studies over 50 years, ASD concordance is 50–90% in monozygotic twins, while the concordance in dizygotic twins is 30% [8–11]. It is now accepted that ASD is a highly inheritable condition, the risk becomes higher with a closer kinship of affected individuals [8]. Known genetic causes include (but not limited to) copy number variants (CNVs), *de novo* single nucleotide variants (SNVs), common genetic variants, mosaicism, non-coding and regulatory pathogenic variations, and inherited recessive variants. However, to date, approx. 70% of the affected individuals have no genetic etiology identified [12].

### *De novo* CNVs

CNVs refer to large deletions or duplications often involving in several genes. The association of phenotype with gene dosage exists, but the confirmation of relationship is often difficult. In 2007, comparative genomic hybridization was used to establish a significant association between *de novo* submicroscopic structural variation and autism [13]. From then on, more CNVs related to autism have been identified. The curated CNVs with ASD from SFARI Gene (https://gene.sfari.org/) are shown in Table 1.

**Table 1:** Known CNVs in ASD

Location	CNV type	Syndrome	Spanning range^*^	Associated/candidate genes
1q21.1	Deletion/Duplication	1q21.1 deletion/duplication syndrome	Chr1:145900678-147965543	-
2p16.3	Deletion/Duplication	NRXN1 deletion syndrome	Chr2:47600165- 53040270	*FBXO11, NRXN1*
2q11.2	Deletion/Duplication	2q11.2 deletion syndrome	Chr2:97739057- 98115695	-
3q29	Deletion/Duplication	3q29 deletion/duplication syndrome	Chr3:195676676-197366632	*DLG1, PAK2, TM4SF19*
5q35	Duplication	5q35 duplication	Chr5:178554060-179589550	-
7q11.23	Deletion/Duplication	Williams–Beuren syndrome/Williams–Beuren duplication syndrome	Chr7:72311894- 74129587	*CLIP2, GTF2I, STX1A*
8p23.1	Deletion/Duplication	8p23.1 deletion/8p23.1 duplication syndrome	Chr8:8123460- 11384691	-
15q13.3	Deletion/Duplication	15q13.3 deletion syndrome/15q13.3 duplication	Chr15:30938215-32914140	*ARHGAP11A, CHRNA7, FAN1, OTUD7A, TRPM1*
16p11.2	Deletion/Duplication	16p11.2 deletion/duplication syndrome	Chr16:29692499-30792499	*CORO1A, KCTD13, MAPK3, SEZ6L2, SRCAP*
16p12.2	Deletion/Duplication	16p12.2 deletion/duplication	Chr16:21356420-21577433	-
16p13.11	Deletion/Duplication	16p13.11 microdeletion syndrome/16p13.11 microduplication	Chr16:14972499-16522499	-
16p13.3	Deletion/Duplication	16p13.3 deletion syndrome/16p13.3 duplication	Chr16:3392370- 5752860	*CREBBP*
17p11.2	Deletion/Duplication	Smith–Magenis syndrome/Potocki–Lupski syndrome	Chr17:16532736-20464365	*RAI1*
17q11.2	Deletion/Duplication	17q11.2 deletion syndrome/17q11.2 duplication	Chr17:29015932-29149664	-
17q12	Deletion/Duplication	17q12 deletion/duplication syndrome	Chr17:37228545-39077997	*CACNB1, KRT26, NR1D1, THRA*
22q11.2	Duplication	22q11.2 duplication syndrome	Chr22:21031117-21651381	*LZTR1*
22q13.3	Deletion	22q13.3 deletion syndrome	Chr22:41122568-49565875	*EP300, TCF20, XRCC6*

* Genomic location is referred to Human Genome GRCh37/hg19.

### *De novo* SNVs

The clinical implementation of trio exome sequencing has shown a significant contribution to the discovery of *de novo* SNVs to autism risk [14–16]. As these variants usually affect a single gene, it is particularly important in emphasizing the underlying neurobiology of *de novo* SNVs associated with autism.

Enhanced bioinformatics analyses integrate evolutionary constraints to identify risk genes with a false discovery rate less than or equal to 0.1. In addition to utilizing probability of loss of function (pLI), missense badness, PolyPhen-2 constraint score, researchers are able to identify variants affecting gene functions by predicted impact [17]. These analyses not only confirm enrichment of *de novo* loss-of-function mutations which affect highly constrained genes, but also identify pathogenic missense mutations. Besides, functional experiments are crucial for these validations to better understand the mechanism of pathogenicity.

The SFARI gene database (https://gene.sfari.org/) has comprehensive and updated information on ASD-associated genes [18]. In the released 2020 Q4 database (updated on 13 January 2021), 1003 genes are divided into score 1 (High Confidence), 2 (Strong Candidate), or 3 (Suggestive Evidence) due to the current evidence to support the function of a certain risk gene in ASD development. According to the gene list, the risk genes have a bias of distribution on each chromosome, for instance, high confidence ASD-associated genes (score 1) mainly clustered on the chromosome X. This bias has confirmed the male-to-female ASD ratio which is approx. 4 to 1 [19]. Transcriptome and whole-genome sequencing (WGS) suggest that ASD risk genes may be involved in dysregulation in specific molecular processes, including chromatin modifications, RNA splicing, signaling pathways, gene expression regulation, neuronal communication, cytoskeletal organization, and cell cycling [20–22]. The curated 889 ASD risk genes with SNVs from SFARI Gene (https://gene.sfari.org/) are listed in **Supplementary Table S1** supported by evidence from literature.

### Common genetic variants

Common genetic variants are those variants with higher allele frequencies (usually greater than 0.05). Each of the variants has a small effect on ASD, or together with environmental factors, resulting in an individual bypassing a risk threshold to develop into the disease [4,23,24]. This is also called a polygenic model. Polygenic models are supported by the following multiple lines of evidence: (1) the genetic factors are highly and repeatedly inherited in ASD families [10,25]. (2) The proportion of related phenotypes such as social and behavioral problem is higher in the first-degree relatives of children with ASD compared with the general population [26,27]. (3) Through analyzing the single-nucleotide polymorphism (SNP) data, inherited common variants (minor allele frequency > 0.05) and variants marked by common genetic variants account for a large proportion of the ASD risk in total [3,4,28].

Due to the limited sample size of ASD individuals, specific common variants had not been identified until 2019 by the large-scale genome-wide association study (GWAS) in autism study [29]. Several significant common risk loci delineating the genetic heterogeneity of phenotypic subgroups have been found to reveal that common variants play a large role in high-functioning autism. It has shown that common variants in autism are enriched in regulatory elements, which are predicted to influence the development of the human cortex by utilizing Hi-C data from the developing fetal brain [29]. With increasing GWAS sample size and power, as well as functional experiments, more common risk loci will be identified, which will provide more genetic data for further investigation. The 316 curated known genes with common variants of ASD from SFARI Gene (https://gene.sfari.org/) are summarized in **Supplememtary Table S2** supported by evidence from literature.

### Mosaicism

Mosaic mutations are *de novo* variants occurring after fertilization, only involving some cell lineages of the body. The proportion of autism cases affected by somatic variants is unknown, while recent studies revealed that 0–7.5% of *de novo* mutations in autism were postzygotic mosaic mutations [30,31]. Despite the limitation of small available samples and the absence of parental samples, studies of mosaic mutations in postmortem brain tissue suggest the presence of damaging mosaic mutations in some autism brains. Targeted sequencing and WGS in postmortem autism brain identified potentially risk-modifying somatic mutations present in brain DNA. Somatic mutations may also contribute to the risk of ASD by disrupting the gene regulatory elements [32,33].

Research on mosaic mutations in autism provides another approach to understand cells and circuits critical for the underlying neurobiology, which may help to explain a fraction of cases without genetic causes identified. It is worth noting that mutations at low cell proportion in the brain might not be detectable in peripheral DNA, while peripherally detected somatic mutations may be present in different cell types and distributions within the brain [12]. Single-cell sequencing (scSeq) provides a new solution to identify somatic mutations in different cell lineages.

### Non-coding and regulatory pathogenic variants

*De novo* and inherited non-coding variants have shown to be involved in autism risk [34–38]. Due to lack of robust functional categorization of the non-coding genomic structures, bioinformatics and experimental approaches are often adopted to pursue this issue. Comparative genomics technology is used to identify regions of the human genome with accelerated divergence, or human accelerated regions (HARs), from evolutionarily conserved sequences, specifically reflecting critical function in the human beings, of which many regions are predicted as regulatory function in brain development. A significant amount of both *de novo* CNVs and biallelic SNVs in individuals with autism have been identified through HAR analyses [35,36]. WGS has detected multiple smaller and gene-disruptive CNVs involving dosage sensitivity. Many neurodevelopmental genes such as *ARID1B, SCN2A, NR3C2, PRKCA DSCAM, DISC1, WNT7A, RBFOX1, MBD5, CANX, SAE1*, and *PIK3CA* are associated with ASD by affecting putative regulatory elements of these genes [37]. Further functional experiments (*in vitro* cellular reporter assays and *in vivo* mouse models) provide more evidence with respect to the impact of the identified variants, locating at the active enhancers of *CUX1, PTBP2, GPC4, CDKL5*, therefore such ASD or neural function linked biallelic HAR mutations have been revealed [36]. In addition, it has been shown that paternally inherited *cis*-regulatory structural variants (SVs), including but not limited to *CNTN4, LEO1, RAF1*, and *MEST*, are preferentially transmitted to affected offspring [35].

By utilizing a deep learning method in combination with extensive experimental data, *de novo* variants in probands in the Simons Simplex Collection were annotated [39]. They demonstrated that regulatory *de novo* mutations in probands had a significantly higher predicted functional impact than those in unaffected siblings, while some of the variants were involved in the regulation of previously identified biological pathways, suggesting that both non-coding and coding variations may have effects on the risk of ASD.

Another creative approach using rigorous and unbiased genome-wide category-based *de novo* risk score is adopted to reveal that *de novo* mutations at distal conserved promotors could increase autism risk [38]. Larger samples are required to determine the nuances of how non-coding and regulatory variations affect risk of ASD.

### Inherited recessive variants

It was predicted that inherited recessive variants contribute to autism risk in 1985 [40]. Whole-exome sequencing (WES) has identified rare *de novo* heterozygous mutations, as well as rare recessive mutations inherited from consanguineous families [41,42], indicating that inherited recessive variants play a role in autism liability [40,41].

A study in 2019 has estimated that approx. 5% of total ASD cases are caused by biallelic loss-of-function or damaging missense mutations. An excess of damaging biallelic missense variation was significantly enriched in cases than in controls. This study agreed with the conclusion of earlier studies that females have protective effect for rare recessive mutations [43]. We curated 207 known ASD recessive inherited risk genes from SFARI Gene (https://gene.sfari.org/) in **Supplementary Table S3** supported by evidence from literature.

In conclusion, current studies have mostly focused on protein-truncating variants and recurrent CNVs, however, the contribution of non-coding variants is largely unknown or underestimated. In fact, non-coding variants account for a considerable amount of ASD cases with known molecular etiology. The percentages of ASD individuals harboring known mutations are syndromic (3.4%), *de novo* SNVs (1.34%), and CNVs (1.28%). The heritability of autism in addictive genetic effect (rare inherited and common inherited) is estimated to be 52%, and the non-additive genetic effect is approx. 7% including *de novo* mutations and non-additive effects [4,44]. To ascertain the genetic basis of ASD, more attention should be paid on the non-coding regions of the genome structure. Non-coding variants contribute to ASD development at a complicated mode. It has been revealed that disrupted non-coding RNAs, regulatory elements, or 3D chromatin conformation have profound effects on ASD and neurodevelopment [45].

## ASD and big genomics data

**Figure 1:**
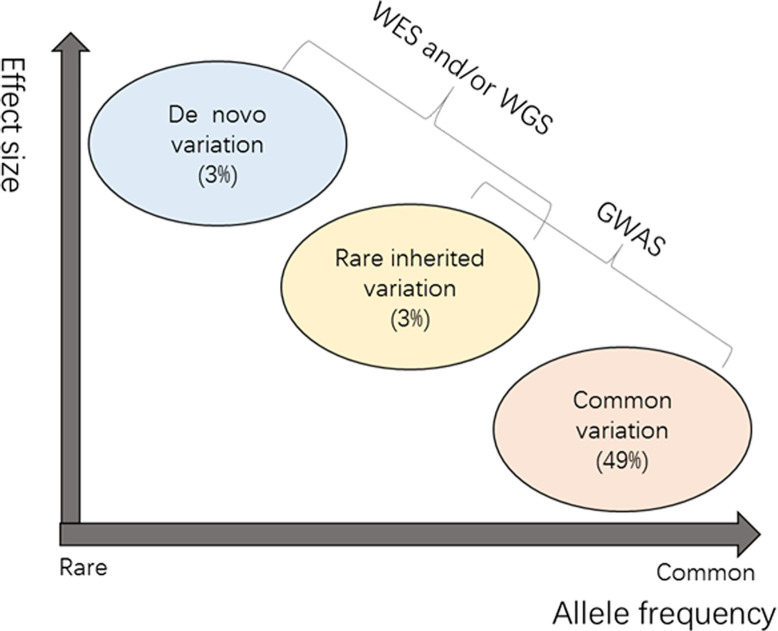
Genetic architecture of ASD This is a sketch map showing liabilities in three mutation classes, namely common variation (minor allele frequency (MAF) > 5%) and rare inherited variation (MAF < 1%) and *de novo* variation [4]. The x-axis represents the allele frequency from rare to common. The effect size is increasing from bottom to top. The liabilities in different mutation classes are shown in brackets.

Family and twin studies on ASD have shown the importance of common variants in heritability, as well as the large effects of rare and *de novo* variants in individuals [4] (Figure 1). Genomic studies of ASD we refer here mainly include results from GWAS, WES, and WGS. Comparison of advantages and limitations of these genomics studies is summarized in Table 2.

**Table 2:** Comparison of advantages and limitations of genomic studies in ASD research

Approach	Advantages	Disadvantages
GWAS	1) Relatively easy to perform technically 2) Meta-analysis can be used to increase the statistical power 3) Common variant in common disease model is often adopted	1) Sample size limitation is common in association studies 2) Population stratification or selection criteria for cases and controls are confounders in GWAS or meta-analysis 3) Replication of risk loci in different populations or labs is rarely seen 4) Biological relevance of risk loci is often very difficult to validate, especially for those SNPs located in the intergenic region 5) Rare pathogenic allele is often missing
WES	1) Sample collection is easy to obtain in a single center 2) Trio-WES is often adopted to analyze allele transmission and *de novo* variants 3) Candidate genes are easily selected from pathogenic or likely pathogenic variants 4) Rare variants are often focused 5) Cost-effective in identifying coding variants or *de novo* variants	1) Variants are limited to the exonic regions or exon/intron boundaries 2) VUSs are difficult to interpretate without functional assays 3) Multicenter validation is often limited
WGS	1) Sample collection is easy 2) SNV, CNV, SV can be simultaneously analyzed at the genomic level 3) *De novo* variants can be found in Trio-WGS 4) Variants from both coding and non-coding regions are covered	1) Relatively expensive for one sample 2) Length and depth of sequencing reads are important for the quality in expense of the costs 3) Variants in non-coding regions are difficult to replicate in animal models 4) Accuracy of prediction models for SVs needs to be improved

### Common variants in GWAS

GWAS is a widely adopted approach to associate common variants with complex diseases. From 2009 on, many GWASs of ASD have been conducted [3,46–49]. However, no variant has been robustly replicated in these GWASs due to sample size limitation. One way of increasing sample size is to perform meta-analysis study by integrating individuals from different ASD cohorts. Recently, a meta-analysis based GWAS with 18381 ASD cases and 27969 controls including iPSYCH samples, Psychiatric Genomic Consortium samples, and five follow-up samples identified five genome-wide significant loci at *LINC02790, AC120036.1, AC090987.1, AC025839.1, RSU1* [29].

Another way is to take advantage of phenotypic similarity to boost statistical power. A cross-trait meta-analysis was conducted based on GWAS data which included 65967 schizophrenia, 41653 bipolar disorder, 46350 ASD, 55374 attention deficit hyperactivity disorder, and 688809 depressions [50]. This study identified ten ASD associated genomic loci at genes *RSRC1, AC099520.1, GALNT10, AC003044.1, SOX7, SORCS3, RBFOX1, DCC, MACROD2, ZNF877P*. Cross-Disorder Group of the Psychiatric Genomics Consortium analyzed 232964 cases and 494162 controls from GWAS of anorexia nervosa, attention-deficit/hyperactivity disorder, ASD, bipolar disorder, major depression, obsessive-compulsive disorder, schizophrenia, and Tourette syndrome [51]. This meta-analysis detected 109 loci across eight disorders, where 23 genomic loci are commonly associated with four or more disorders including genomic loci at the transcription start sites of *DCAF4L1, CTNND1, MRPS33, DFNA5* and at the gene bodies of *DCC, RBFOX1, SORCS3, PLCL1, RGS6, CHADL, KCNB1, SOX5*. In addition to increased sample size, functional annotations on common variants tend to incorporate with multiomics datasets, i.e. expression data from brain [52], HiC seq [29], in order to explain the effects of common variants on their molecular pathways. It should be noticed that GWAS has largely conducted in samples of European origin, which is inefficient to capture population-specific signals.

### Rare and *de novo* variants from WES

The remarkable work on the identification of rare and *de novo* mutations in ASD is from the applications in high-throughput sequencing as well as large family-based cohort. At coding regions, the functional roles of rare and *de novo* mutations are assessed by the potential impact on protein function and structure. After the initial step, discovery and prioritization of ASD-related variants can be done by group-wise tests, e.g. Transmission and *De Novo* Association test (TDNA), giving the low frequency of rare and *de novo* mutations. Current evidence of ASD genes highlights the potential pathogenic role of genes carrying protein truncating variants and probably damaging missense variants. TDNA evaluates mutation burden in a gene-based model with weighted categories of above-mentioned two types of mutations. Researchers applied this model to 3871 cases and 9937 controls, where 22 autosomal genes were implicated including *ADNP, ANK2, ARID1B, CHD8, CUL3, DYRK1A, GRIN2B, KATNAL2, POGZ, SCN2A, SUV420H1, SYNGAP1, TBR1, ASXL3, BCL11A, CACNA2D3, MLL3, CTTNBP2, GABRB3, PTEN, RELN, MIB1* [53]. Along with the growing sample size and heterogeneous insight of ASD, several refined versions are developed by either multiple population data [54], or probable intolerance of loss-of-function variation score (pLI) as a continuous metric to weight mutations [21].

As such, the TDNA model and other group-wise tests serve as a basis in prioritizing ASD genes and modified models could add new insights into ASD etiology, but the gene list generated by group-wise tests could be large and further refinement may be required. Enrichment analysis, protein–protein interaction (PPI) networks are useful approaches in refining the gene list [55]. The key to these approaches is either to find out the significant depleted or enriched groups of genes based on known knowledge, or to prioritize novel genes or weakly associated genes via interaction maps.

Another commonly applied method in prioritizing ASD genes is network analysis. For example, Detecting Association With Networks (DAWN) is a Hidden Markov Model (HMM)-based approach which models two sets of data, rare variants, and gene co-expression. Combining TADA scores and BrainSpan gene co-expression data, DAWN identified 102 genes through complex gene networks from 35584 individuals with 11986 ASD [54]. Majority of these genes are expressed in brain, where 53 are ASD predominant genes (e.g. *ASH1L, CHD8, KMT5B, DEAF1, KDM6B, ANK2, SHANK3, PTEN, DSCAM*), 49 are ASD and neurodevelopmental delay genes (e.g. *ADNP, ANKRD11, ARID1B, MED13L, CHD2,TLK2, CTNNB1, POGZ, FOXP1, SLC6A1, SYNGAP1, GRIN2B, SCN2A, DYRK1A*).

One important aspect is that all these approaches alone are useful in prioritizing candidate genes, but an increasing number of studies have applied combined or refined approaches to boost discovery power giving the complexity of ASD genetics. Moreover, multidimensional data are used to understand the underlying molecular mechanisms of ASD in recent years.

### Rare and *de novo* variants from WGS

The basic ide a of prioritizing variants from WGS is very similar to WES, in which functional annotations and grouping are usually the initial step. By doing so, rare and *de novo* variants can be analyzed collectively in statistical models. However, WGS generates comprehensive types of variants encompassing SNVs, InDels and SVs, as well as vast number of variants locating at non-coding regions compared with WES, where methods have to be modified. The Category-Wide Association Study (CWAS), akin to GWAS, with SNP substituted for annotation categories, can be applied to variants generated from WGS. For example, CWAS was applied to all kinds of variants by grouping variants into various functional regions, such as gene-coding regions, conserved regions, with modest findings only [38]. Like GWAS, a big sample size increases the power of detection. Within 1902 quartet families (parents and one ASD-affected child, one healthy sibling), indications of *de novo* mutations at promoter regions by CWAS were found [34].

Focusing on only SVs, enrichment analysis and group-wise tests are commonly used. Brandler et al. applied enrichment analysis over several functional catalogs in total of 9274 subjects from 2600 families [35]. Burden test, a type of group-wise test, is applied for the comparisons of variant frequencies between cases and controls where novel loci and known tandem repeat expansions were found [56]. The identified SVs often need additional methods for further refinement. A method that adopted the idea that SVs lead to long-range interactions has been developed in our lab [57]. A similar method that took advantage of known disease-related SVs is used to refine the list of candidate SVs [58]. Furthermore, giving the complexity of SVs and a variety of source data providers, many efforts have been done to integrate SVs into a clean dataset. A unified workflow is necessary to accelerate the study on SVs in ASD.

Together, genomics studies established the importance of *de novo* mutations at protein-coding regions in ASD development, as well as with growing evidence for the modest effects of non-coding regions. Multidimensional data may also leverage our knowledge on molecular etiology of ASD, such as expression data [59–61] and epigenetics data [62].

## ASD models

### Cell models

Successful reprogramming of adult somatic cells transforms differentiated cells into induced pluripotent stem cells (iPSCs) [63]. The main features of iPSCs include the self-renewal capability and differentiation potential. As human iPSCs can differentiate into a variety of cell types, somatic cells obtained directly from patients could be induced into specialized *in vitro* cell models to study the disease mechanisms. Compared with other *in vitro* cell models, iPSC models directly derived from patients keep the genetic background of the ASD patients [64,65]. With the cellular models obtained from patients, the biological basis or molecular mechanism of the disorders is investigated to validate the association between the genotype and phenotype, and to develop new cell or pharmacological therapeutic approaches [64,65]. In recent years, numerous *in vitro* cell models have been utilized to study the mechanism of ASD (**Supplementary Table S4**). Challenge of *in vitro* cellular models is how to model the cellular and physiological phenotypes which are most relevant to ASD patients. Although there are a lot of advantages of *in vitro* cell models, cells in culture can not fully recapitulate all the complex mechanism of ASD.

### Animal models

Flourishing genetic achievements has accelerated the generation and characterization of different types of animal models of ASD. The commonly used animal models include zebrafish, mouse, rat, and non-human primates (**Supplementary Table S5**). Mouse or rat genes are highly homologous to human genes, compared with other animal models. Mouse models provide an experimental platform to study molecular mechanisms, cellular pathways, circular disturbances and behavioral analyses of ASD, offering the opportunity to explore whether the behavioral abnormalities could be reversed by potential therapeutics before translating them to humans [66]. However, there are still some drawbacks of mouse models. Neuropsychiatric behaviors assessed by psychiatrists are difficult to be measured or recorded in mice (e.g. language). Comorbidities such as sensory dysfunction, learning deficits, locomotor dysfunction, fear and anxiety could confound with core ASD phenotypes in human during the assessment. Primate models or invertebrates are complementary to mouse models [67]. Non-human primates models could simulate the complex behaviors and higher cortical functions of human, whereas zebrafish and invertebrates could be efficiently manipulated in large-scale parallel experiments [44,67,68]. However, due to the non-conserved non-coding regions in animals, it is difficult to model those SNVs, CNVs, or SVs in the non-coding regions.

## Pathogenic mechanisms of ASD development

There are plenty of hypothetical pathophysiological mechanisms of ASD, which have been tested by studies in humans or model systems from different aspects. Most of these mechanisms demand more work to figure out the exact molecular pathways. Furthermore, these models overlap at some extent. Different stages of brain development may share the same genes or molecular pathways. More interestingly, how early developmental disruption correlates to phenotypes after birth or at later ages remains unknown. According to recent studies, it is shown that the abnormal development of ASD may begin in prenatal period [69]. ASD risk genes are expressed in prenatal and postnatal stages, most of which are expressed broadly as regulatory genes in a variety of biological processes in brain development. The aberrations of ASD risk genes could cause abnormalities through disrupting regulatory networks and dysregulating key signaling pathways such as PI3K/AKT, RAS/ERK, Notch, and Wnt/ β-catenin.

From the first to the third trimesters, namely *Epoch-1*, with the combination of broadly expressed regulatory risk genes and brain-specific risk genes, many embryonic development processes (e.g. cell proliferation, neurogenesis, cell fate determination, and migration) are disrupted. In the third trimester and early postnatal period, namely *Epoch-2*, there may exist dysregulation of cortical wiring (including neurite outgrowth, neural network organization, synaptogenesis) due to a different set of genes.

Here we summarize the main ASD-related genes of different brain developmental stages in Table 3 [70–85], and the prevailing hypotheses of ASD development [44] are discussed below.

**Table 3:** ASD risk genes and associated affected developmental processes

Affected developmental process	Gene symbol
Neuron migration	*ASTN2, AUTS2, CHD8, CNTNAP2, DLX1/2, FOXP1, LIS1, NCAM2, NCKAP1, NDE1, RELN, TBR1, TCF4*
Cell–cell adhesion	*ASTN2, CHD8,CHD9, CHD10, CHD13, NRLG1/2/3/4/4Y, NRXN1/2/3*
Neurite growth	*AUTS2, CSDE1, CTNND2, DOCK4, KIAA2022/NEXMIF, MECP2, NF1, PTEN, RELN, TAOK2, TSC1, TSC2, UBE3A*,
Synapse formation	*CNTNAP2, CTTNBP2, FMR1, TAOK2*
Synaptic function	*CNTNAP2, DIP2A, NRXN1/2/3, SYN1/2/3*
Synaptogenesis	*DLG4, GPHN, MECP2, NRLG1/2/3/4/4Y, NRXN1/2/3, PTEN, SHANK1/2/3, SYN1/2/3, TSC1, TSC2*
Synaptic plasticity	*FMR1, MECP2, SHANK1/2/3, TSC1, TSC2, UBE3A*
Translation	*AGO1, CNOT3, DYRK1A, eEF1A2, eEF2, eIF3g, eIF4B, eIF4E, FMR1, JAKMIP1, PABPC1, PTEN, RPL10, RPS6, TNRC6B, UPF3B*
Intracellular transport	*CYFIP1, LIS1, NDE1,WDFY3*
Neurogenesis	*LIS1, NDE1, PTEN, WDFY3*
Transcription	*MECP2, TCF4*

### Dysregulation of fetal cortical development

Multiple lines of evidence from human genetic studies and postmortem studies support the notion that dysregulation of fetal cortical development could result in ASD [86–88]. Neuropathological studies have revealed a number of cortical developmental errors such as smaller neuron size, more neuron number, mislocated neurons, misoriented pyramidal neurons, disrupted lamination, reduced white matter tracks, and abnormal dendrites in ASD patients [86]. Other studies showed that the cortical minicolumn, which is a basic processing unit of cortical circuits, is more narrow and densely packed [89]. Patches of cortical cells could not be laminated regularly due to lack of specific laminar markers [90]. The brain size is reduced at birth but overgrown during childhood in individuals affected with ASD [91].

It has been reported that the target genes involved in the mTOR pathway harbor more ASD-related variants, and may affect the regulation of processes of cell proliferation, cell growth, and neuronal morphogenesis [92]. The target genes involved in the Wnt pathway may regulate the courses of radial glia self-renewal, neuronal differentiation, and brain dorsoventral pattern [93]. We found that BLOS2 interacts with Notch1 to mediate the endolysosomal trafficking of Notch1. Loss of BLOS2 leads to elevated Notch signaling, which consequently increases the proliferation of neural progenitor cells and inhibits neuronal differentiation during cortical development [94]. Target genes involved in the BAF complex (a multisubunit complex mediating chromatin remodeling) may regulate neurogenesis and neuronal morphogenesis [95]. Mutations of the genes involved in these pathways are considered to play a role in cortical development.

### Synaptic dysfunction

Neuropathological studies have provided strong genetic evidence for synaptic dysfunction. Mutations in genes encoding synaptic cell-adhesion molecules, excitatory and inhibitory synaptic scaffolding molecules, the excitatory glutamatergic receptor, inhibitory GABAergic receptor subunits, inhibitory synaptic scaffolding molecule gephyrin and neurotransmitter release regulators are associated with ASD in numerous studies. Namely, these molecules include neurexins [53,96,97], neuroligins [98], the SH3 and multiple ankyrin-repeat domain (SHANK) proteins [53,97], GRIN2B, GABAR [53,96], GPHN [53], the synaptotagmins [16,53], and synapsins [53,99].

The dysregulation in synaptogenesis and synaptic transmission have effects on ASD [100]. Meanwhile, the glutamatergic and GABAergic synaptic dysfunction raise the hypothesis that disruption in the excitatory/inhibitory (E/I) balance leads to ASD [101]. However, E/I imbalance is also frequently observed in other neuropsychiatric disorders such as epilepsy, Alzheimer’s disease, and schizophrenia [102]. Thus, how E/I imbalance has an effect on ASD pathophysiology demands dissecting its spatiotemporal dynamics, which means we need to figure out whether there is a key period or whether it is circuit specific that an E/I imbalance leads to ASD-relevant behavior in numerous ASD models. Moreover, an E/I imbalance could result from either synaptic physiology changes or altered cell fates that result in shifted ratio of inhibitory and excitatory synaptic neurons [103].

### Abnormalities of gene transcription and translation

Neuronal activity could dynamically regulate gene transcription and protein translation in neurons, to ensure that specific gene could expressed spatially or contextually within subcellular partition [104]. Many studies have shown that disruption of this activity-dependent gene transcription and translation may cause ASD. Mutations of risk genes or risk loci such as *TSC1, TSC2, FMR1* and dup15q11-q13 suggest that ASD patients may result from dysregulated neuronal translation [92,105]. Studies in mice emphasize the viewpoint of molecular enrichment between synaptic function, synaptic plasticity, and translational regulation [106]. Synaptic pruning and stability are also regulated though activity-dependent transcription and translation [107]. As a supporting evidence, it has been reported that ASD patients present increased dendritic spine density in the temporal lobe [108].

Many ASD risk genes are confirmed to have an increased risk for ASD, these genes could be transcriptionally co-regulated (such as *MEF2A, MEF2C*, and *SATB1*) as well as translationally regulated (e.g. *FMR1*), suggesting that a potential convergent mechanism in ASD development is activity-dependent gene regulation [109]. It is critical to explore the link between ASD risk gene-related changes in synapse dynamics and the specific phenotypes presented in ASD models and patients. There is an assumption that small differences in synaptic function and timing will dysregulate the linkage between higher order association regions, including the frontal–parietal, frontal–temporal, and frontal–striatal circuits which mediate social behaviors [110]. To investigate the linkage between synaptic dysfunction and multiple heterogeneous phenotypes in ASD patients, it is necessary to study transcriptional and translational regulation related to spatiotemporal dynamics and the differences in micro- and macro-circuit connection [111].

### Altered neural circuitry

Studies of neuroimaging and neuropathology in ASD patients imply that within the cortex and in cortico–striatal circuits, there exists disruption of resting state network activity as well as altered macrocircuit connectivity [112,113]. By utilizing systematic imaging in ASD-like mouse models, studies illustrated that the parieto–temporal lobe, the cerebellar cortex, the frontal lobe, the hypothalamus, and the striatum are the most commonly affected regions, shared by all the 26 mouse models [114]. Another candidate ASD region is the amygdala, which plays a critical role in modulating the emotion of fear as well as social behaviors [113,115]. Besides, striatal dysfunction is likely the neural basis for repetitive behavior as well as motor routine learning both in mice and in humans [116].

Except for the frontal circuits, cerebellar function also plays a role in social behavior. Individuals with ASD have deficient processing of abstract animations [117] and body motion [100]. A recent study showed that both degenerative cerebellar disease and autistic patients could not handle the immediate perceptual component of the mental state recognition (for example, to distinguish other people’s psychosis from their eye expression) and superior conceptual level of mentalization (for example, to tell a false opinion) [118]. Additionally, functional MRI (fMRI) studies illustrated that social impairment typically observed in ASD patients may link the dysregulation of cerebellar outputs to default network brain areas [119]. Differences in cerebellar volume [120] and decreased gray matter volumes in given cerebellar areas [121] have been recognized from the earlier neuroimaging studies of ASD. Early cerebellar damage is responsible for increased internalizing behaviors, emotional and attentional deficits, and social contact disorder [122], suggesting that the autistic characteristic behaviors could derive from atypical cerebellar development [123].

From cells to systems, animal models have also shown how the cerebellum is involved in autism [124,125]. All of the 26 autism mouse models revealed cerebellar abnormalities through the clustering analysis [126]. Knockout of *Tsc1* in mouse cerebellar Purkinje cells could cause core ASD-like behaviors [127], suggesting that social deficits of autism could be derived from cerebellar dysfunction in mice. As in humans, disrupted cerebellar development in early stage in rodents may result in ASD-like phenotypes [128]. However, how to correlate the phenotypes of mouse to human circuits is still a challenging task. Moreover, many relevant brain areas in humans, like the frontal and temporal lobes, have changes dramatically during the evolution of primates [129]. Together with mouse models, primate models can be a complement to utilize comparative studies, which may reveal new candidate brain circuits related to ASD pathogenesis.

Studies of cerebellar structural and functional connectivity in ASD patients provide evidence for the involvement of cerebellar in autism [130]. Decreased cerebellar white matter density [131] and larger cerebellar white matter volume [132] in autism has been reported. Diffusion imaging studies showed that the cerebellum and the cerebral cortex connected pathways change its integrity [133], suggesting that in the middle and superior cerebellar peduncles, fractional anisotropy (FA) could be decreased, while mean diffusivity increased. Thus, cerebellar white matter volume could be utilized as a predictor of future autism diagnosis [134], while decreased cerebellar FA correlates with autism severity [135].

The findings of resting-state functional connectivity implied atypical cerebro–cerebellar networks of ASD, presenting decreased connectivity within settled networks, most of which worked for social intercourse. Besides, in autism there are increased connectivity between cerebellum non-motor areas and sensori-motor cerebral cortical areas, implying atypical linkage between sensori-motor and non-motor cerebro–cerebellar circuits [136].

### Dysregulated neuron-glia signaling and neuroinflammation

It is reported that there are numerous activated microglia and astrocytosis in multiple brain areas in brains from individuals with ASD. Imaging studies utilizing positron emission tomography (PET) [137] and studies on postmortem brains [88] show a large number of activated microglial and astrocytosis cells in the brains of ASD affected individuals [138]. Synaptic dysfunction in ASD patients, which causing abnormal synapses numbers, functions, and E/I balance which finally result in autistic phenotype, could be caused by dysregulated synaptic pruning and homeostasis due to the vicious cycle of up-regulated microglia and astrocytes [139,140]. As synaptic development and pruning could be regulated by astrocytes and microglia [141,142], it could provide another clue for therapeutic targets [143].

There is a gender bias in ASD incidence: the affected males are four-times higher than the affected females. In current studies, the properties of microglia have a discrepancy in different sex. Compared with males, the cultured microglial cells of females have stronger ability of phagocytosis [144]. According to a microglia-specific eIF4E overexpressing mice experiment, only male mice showed the phenotype of abnormal behaviors and microglial morphologies ability [145]. Abnormal phagocytic ability of microglia may be the mechanism of activated microglia in ASD brain: the accumulation of degenerated cells and materials caused by impaired phagocytic ability enhancing activation and proliferation of microglia via damage-associated molecular patterns, while the damaged phagocytosis is unaffected [146].

The dramatically increased incidence of ASD may not be simply considered as changes in diagnostic criteria and/or methods [147]. It has shown that environmental factors which affecting immunological responses in human brain may lead to autism. Microglia in fetal brains could be activated by environmental factors through maternal immune activation (MIA), which consequently lead to ASD. In a study of mouse model, infected with microorganisms like herpes simplex virus during pregnancy caused MIA, hyperactivating fetal brain microglia, and finally resulting in autistic behaviors in the offspring [148]. The mechanism of how MIA-induced activated microglia impact the synaptic transmission, causing autistic symptoms is still not fully clear.

### Impaired adult neurogenesis

Neurodevelopment continues after birth. Circuitry maturation or plasticity is further established upon adult neurogenesis. Adult neurogenesis is subject to epigenetic changes. Our results have shown that dysbindin-1C, an isoform of schizophrenia susceptibility gene *DTNBP1*, is involved in the maturation of adult newborn neurons in the dentate gyrus (DG) by regulating the survival of hilar mossy cells [149,150]. Similarly, *FMR1* is involved in the cell survival at the ventral subregion of the DG [151]. FMRP plays an important role in adult hippocampal neurogenesis and hippocampus-dependent learning by regulating the adult neural stem cell (aNSC) fate through the translational regulation of GSK3β [152]. Loss of FMRP compromises the differentiation of aNSCs by impacting many mitosis and neurogenesis genes at transcription or translation level. In addition, knockdown of necdin, an FMRP-repressed transcriptional factor, rescues aNSC differentiation [153]. Another mechanism related to loss of FMRP is the increased protein synthesis of histone acetyltransferase EP300 and ubiquitination-mediated degradation of histone deacetylase HDAC1 in aNSCs [154]. As adult hippocampal neurogenesis converges several pathways, other ASD risk genes may be involved in this process.

### Epigenetic and transcriptomic differences

Except for the *FMR1* gene in regulating histone modifications as mentioned above [154], it has been shown that DNA methylation differences in genomic regions linked with immunity and neuronal regulation in ASD brain [155,156]. Histone H3K27 acetylation is also clarified to have an effect on genes involved in synaptic transmission and morphogenesis [157]. By integrating omics studies of mRNA expression, miRNA expression, DNA methylation, and histone acetylation from ASD and control brains, researchers have dissected a convergent molecular subtype of ASD with shared dysregulation across both the epigenome and transcriptome. They expanded the repertoire of differentially expressed genes in ASD as well as identified a component of up-regulated immune processes which is related to hypomethylation. By utilizing eQTL and chromosome conformation datasets, differentially acetylated regions with their cognate genes could imply an enrichment of ASD genetic risk variants in hyperacetylated non-coding regulatory regions linked to neuronal genes [158]. Besides, the expression differences in one-carbon metabolites transcript of *TGR-AS1, SQSTM1, HLA-C*, and *RFESD* were identified to be related to ASD through differential expression analysis [159].

## Conclusion and perspectives

ASD has a complex origin, arising from both genetic risks and environmental exposures. As the increase in sample sizes and development of statistical and biological approaches, there are more and more evidence to uncover diverse genetic mechanisms and biological pathways of ASD. Big genomics data and bioinformatics and experimental innovation accelerate the investigation of ASD genetic risk factors, especially the common variants and those variants in non-coding and regulatory regions.

A challenge in the omics era is how to integrate the meaningful link from multiomics data. Meaningful genomic variants can be evidenced by transcriptomic and proteomic data. scSeq is now a trend to explore the abnormalities in spatiotemporal ASD brains and in inflammatory cells of both brain regions and bloodstream that are potential features or biomarkers of ASD.

In future, along with the understanding of the genetics and genomics of ASD, studies should try to integrate the intricate connections among different genetic sources, biological pathways and brain connectomes, exploiting potential biomarkers and therapeutics for individuals affected with ASD. It is well accepted that ASD can be categorized into different subtypes by inputting DSM-V scores, NMR images, and omics data. Uncovering these subtypes will be another challenge in ASD research. Combined with new science and technology such as artificial intelligence (AI), it could be possible to improve the genetic diagnosis and intervention for this condition.

## Supplementary Material

Supplementary Tables S1-S5

## Abbreviations

aNSC: adult neural stem cell
ASD: autism spectrum disorder
CNV: copy number variant
CWAS: category-wide association study
DAWN: Detecting Association With Networks
DG: dentate gyrus
E/I: excitatory/inhibitory
eQTL: expression quantitative trait loci
FA: fractional anisotropy
GWAS: genome-wide association study
HAR: human accelerated region
InDel: insertion and deletion
iPSC: induced pluripotent stem cell
MIA: maternal immune activation
scSeq: single-cell sequencing
SNP: single-nucleotide polymorphism
SNV: single nucleotide variant
SV: structural variant
TDNA: Transmission and *De Novo* Association test
WES: whole-exome sequencing
WGS: whole-genome sequencing
