# Supplementary material for: Biological implications of genetic variations in autism spectrum disorders from genomics studies
Source: Biosci Rep. 2021 Jul 22;41(7):BSR20210593. doi: 10.1042/BSR20210593 (PMC8298259; doi:10.1042/BSR20210593)
Supplement: Supplementary Tables S1-S5 [file BSR-2021-0593C_supp.pdf]

Table S1. ASD risk genes with single nucleotide variants.

Gene symbol	Gene name	Ensembl ID	Chromosome	Genetics category
ABAT	4-aminobutyrate aminotransferase	ENSG00000183044	16	Rare Single Gene Mutation, Genetic Association
ABCA10	ATP-binding cassette, sub-family A (ABC1), member 10	ENSG00000154263	17	Rare Single Gene Mutation
ABCA13	ATP binding cassette subfamily A member 13	ENSG00000179869	7	Rare Single Gene Mutation, Functional
ABCA7	ATP-binding cassette, sub-family A (ABC1), member 7	ENSG00000064687	19	Rare Single Gene Mutation
ACE	angiotensin I converting enzyme	ENSG00000159640	17	Rare Single Gene Mutation, Genetic Association
ACHE	Acetylcholinesterase (Yt blood group)	ENSG00000087085	7	Rare Single Gene Mutation
ACTB	actin beta	ENSG00000075624	7	Rare Single Gene Mutation, Syndromic
ACTL6B	actin like 6B	ENSG00000077080	7	Rare Single Gene Mutation, Syndromic
ACTN4	actinin alpha 4	ENSG00000130402	19	Rare Single Gene Mutation
ACY1	aminoacylase 1	ENSG00000243989	3	Rare Single Gene Mutation, Syndromic
ADA	adenosine deaminase	ENSG00000196839	20	Rare Single Gene Mutation, Genetic Association
ADCY3	adenylate cyclase 3	ENSG00000138031	2	Rare Single Gene Mutation
ADCY5	Adenylate cyclase 5	ENSG00000173175	3	Rare Single Gene Mutation
ADK	adenosine kinase	ENSG00000156110	10	Rare Single Gene Mutation
ADNP	Activity-dependent neuroprotector homeobox	ENSG00000101126	20	Rare Single Gene Mutation, Syndromic, Functional
ADORA3	Adenosine A3 receptor	ENSG00000282608	1	Rare Single Gene Mutation, Functional
ADSL	adenylosuccinate lyase	ENSG00000239900	22	Rare Single Gene Mutation, Syndromic
AFF2	AF4/FMR2 family, member 2	ENSG00000155966	X	Rare Single Gene Mutation, Syndromic
AGAP1	ArfGAP with GTPase domain, ankyrin repeat and PH domain 1	ENSG00000157985	2	Rare Single Gene Mutation

AGAP2	ArfGAP with GTPase domain, ankyrin repeat and PH domain 2	ENSG00000135439	12	Rare Single Gene Mutation
ADSS2	adenylosuccinate synthase 2	ENSG00000035687	1	Rare Single Gene Mutation
AGBL4	ATP/GTP binding protein-like 4	ENSG00000186094	1	Rare Single Gene Mutation
AGMO	alkylglycerol monooxygenase	ENSG00000187546	7	Rare Single Gene Mutation, Genetic Association
AGO1	argonaute 1, RISC catalytic component	ENSG00000092847	1	Rare Single Gene Mutation
AGO3	argonaute RISC catalytic component 3	ENSG00000126070	1	Rare Single Gene Mutation
AGO4	argonaute RISC catalytic component 4	ENSG00000134698	1	Rare Single Gene Mutation
AGTR2	angiotensin II receptor, type 2	ENSG00000180772	X	Rare Single Gene Mutation
AHDC1	AT-hook DNA binding motif containing 1	ENSG00000126705	1	Rare Single Gene Mutation, Syndromic
AHI1	Abelson helper integration site 1	ENSG00000135541	6	Rare Single Gene Mutation, Syndromic, Genetic Association
AKAP9	A kinase (PRKA) anchor protein 9	ENSG00000127914	7	Rare Single Gene Mutation
ALDH1A3	aldehyde dehydrogenase 1 family member A3	ENSG00000184254	15	Rare Single Gene Mutation, Syndromic
ALDH5A1	aldehyde dehydrogenase 5 family, member A1 (succinate-semialdehyde dehydrogenase)	ENSG00000112294	6	Rare Single Gene Mutation, Syndromic
AMPD1	Adenosine monophosphate deaminase 1	ENSG00000116748	1	Rare Single Gene Mutation, Genetic Association
AMT	Aminomethyltransferase	ENSG00000145020	3	Rare Single Gene Mutation

ANK2	Ankyrin 2, neuronal	ENSG00000145362	4	Rare Single Gene Mutation
ANK3	ankyrin 3	ENSG00000151150	10	Rare Single Gene Mutation, Genetic Association
AGAP5	ArfGAP with GTPase domain, ankyrin repeat and PH domain 5	ENSG00000172650	10	Rare Single Gene Mutation
ANKRD11	ankyrin repeat domain 11	ENSG00000167522	16	Rare Single Gene Mutation, Syndromic
ANKS1B	ankyrin repeat and sterile alpha motif domain containing 1B	ENSG00000185046	12	Rare Single Gene Mutation, Syndromic
ANXA1	Annexin A1	ENSG00000135046	9	Rare Single Gene Mutation
AP1S2	adaptor related protein complex 1 sigma 2 subunit	ENSG00000182287	X	Rare Single Gene Mutation, Syndromic
APBA2	amyloid beta (A4) precursor protein-binding, family A, member 2	ENSG00000034053	15	Rare Single Gene Mutation
APBB1	amyloid beta precursor protein binding family B member 1	ENSG00000166313	11	Rare Single Gene Mutation, Functional
APH1A	APH1A gamma secretase subunit	ENSG00000117362	1	Rare Single Gene Mutation
ARHGAP11B	Rho GTPase activating protein 11B	ENSG00000285077	15	Rare Single Gene Mutation
ARHGAP32	Rho GTPase activating protein 32	ENSG00000134909	11	Rare Single Gene Mutation, Functional
ARHGAP5	Rho GTPase activating protein 5	ENSG00000100852	14	Rare Single Gene Mutation
ARHGEF10	Rho guanine nucleotide exchange factor 10	ENSG00000104728	8	Rare Single Gene Mutation, Functional
ARHGEF9	Cdc42 guanine nucleotide exchange factor (GEF) 9	ENSG00000131089	X	Rare Single Gene Mutation, Syndromic
ARID1B	AT-rich interaction domain 1B	ENSG00000049618	6	Rare Single Gene Mutation, Syndromic

ARID2	AT-rich interaction domain 2	ENSG00000189079	12	Rare Single Gene Mutation, Syndromic
ARNT2	aryl-hydrocarbon receptor nuclear translocator 2	ENSG00000172379	15	Rare Single Gene Mutation, Syndromic, Genetic Association
ARX	aristaless related homeobox	ENSG00000004848	X	Rare Single Gene Mutation, Syndromic
ASAP2	ArfGAP with SH3 domain, ankyrin repeat and PH domain 2	ENSG00000151693	2	Rare Single Gene Mutation
ASB14	ankyrin repeat and SOCS box containing 14	ENSG00000239388	3	Rare Single Gene Mutation
ASH1L	Ash1 (absent, small, or homeotic)-like (Drosophila)	ENSG00000116539	1	Rare Single Gene Mutation, Syndromic
ASMT	acetylserotonin O-methyltransferase	ENSG00000196433	X,Y	Rare Single Gene Mutation, Genetic Association
ASPM	abnormal spindle microtubule assembly	ENSG00000066279	1	Rare Single Gene Mutation, Syndromic, Functional
ASTN2	astrotactin 2	ENSG00000148219	9	Rare Single Gene Mutation, Genetic Association
AP2S1	adaptor related protein complex 2 subunit sigma 1	ENSG00000042753	19	Rare Single Gene Mutation
ASXL3	Additional sex combs like 3 (Drosophila)	ENSG00000141431	18	Rare Single Gene Mutation, Syndromic
ATP10A	Probable phospholipid-transporting ATPase VA	ENSG00000206190	15	Rare Single Gene Mutation, Genetic Association, Functional
ATP1A1	ATPase Na+/K+ transporting subunit alpha 1	ENSG00000163399	1	Rare Single Gene Mutation, Syndromic
ATP1A3	ATPase Na+/K+ transporting subunit alpha 3	ENSG00000105409	19	Rare Single Gene Mutation, Syndromic, Functional

ATP2B2	ATPase, Ca++ transporting, plasma membrane 2	ENSG00000157087	3	Rare Single Gene Mutation, Genetic Association
ATP6V0A2	ATPase H+ transporting V0 subunit a2	ENSG00000185344	12	Rare Single Gene Mutation
ATRX	alpha thalassemia/mental retardation syndrome X-linked	ENSG00000085224	X	Rare Single Gene Mutation, Syndromic
AUTS2	autism susceptibility candidate 2	ENSG00000158321	7	Rare Single Gene Mutation, Syndromic, Genetic Association, Functional
AVPR1A	arginine vasopressin receptor 1A	ENSG00000166148	12	Rare Single Gene Mutation, Genetic Association
AZGP1	alpha-2-glycoprotein 1, zinc-binding	ENSG00000160862	7	Rare Single Gene Mutation
BAZ2B	bromodomain adjacent to zinc finger domain 2B	ENSG00000123636	2	Rare Single Gene Mutation
BBS4	Bardet-Biedl syndrome 4	ENSG00000140463	15	Rare Single Gene Mutation, Syndromic
BCAS1	breast carcinoma amplified sequence 1	ENSG00000064787	20	Rare Single Gene Mutation
BCKDK	Branched chain ketoacid dehydrogenase kinase	ENSG00000103507	16	Rare Single Gene Mutation
BCL11A	B-cell CLL/lymphoma 11A (zinc finger protein)	ENSG00000119866	2	Rare Single Gene Mutation, Syndromic
BCORL1	BCL6 corepressor like 1	ENSG00000085185	X	Rare Single Gene Mutation, Syndromic
BIRC6	Baculoviral IAP repeat containing 6	ENSG00000115760	2	Rare Single Gene Mutation
BRAF	v-raf murine sarcoma viral oncogene homolog	ENSG00000157764	7	Rare Single Gene Mutation, Syndromic
BRCA2	breast cancer 2, early onset	ENSG00000139618	13	Rare Single Gene Mutation
BRD4	bromodomain containing 4	ENSG00000141867	19	Rare Single Gene Mutation, Syndromic

BRSK2	BR serine/threonine kinase 2	ENSG00000174672	11	Rare Single Gene Mutation, Syndromic
BTAF1	RNA polymerase II, B-TFIID transcription factor-associated, 170kDa (Mot1 homolog, S. cerevisiae)	ENSG00000095564	10	Rare Single Gene Mutation
BTRC	beta-transducin repeat containing E3 ubiquitin protein ligase	ENSG00000166167	10	Rare Single Gene Mutation
C12orf57	Chromosome 12 open reading frame 57	ENSG00000111678	12	Rare Single Gene Mutation, Syndromic
C15orf62	chromosome 15 open reading frame 62	ENSG00000188277	15	Rare Single Gene Mutation
C4B	complement component 4B	ENSG00000203710	6	Rare Single Gene Mutation, Genetic Association, Functional
CA6	carbonic anhydrase VI	ENSG00000131686	1	Rare Single Gene Mutation
CACNA1A	Calcium channel, voltage-dependent, P/Q type, alpha 1A subunit	ENSG00000141837	19	Rare Single Gene Mutation, Genetic Association
CACNA1B	calcium voltage-gated channel subunit alpha1 B	ENSG00000148408	9	Rare Single Gene Mutation, Syndromic, Genetic Association
CACNA1C	calcium channel, voltage-dependent, L type, alpha 1C subunit	ENSG00000151067	12	Rare Single Gene Mutation, Syndromic, Genetic Association, Functional
CACNA1D	calcium channel, voltage-dependent, L type, alpha 1D	ENSG00000157388	3	Rare Single Gene Mutation, Syndromic, Genetic Association, Functional
CACNA1E	calcium voltage-gated channel subunit alpha1 E	ENSG00000198216	1	Rare Single Gene Mutation
CACNA1F	calcium channel, voltage-dependent, alpha 1F	ENSG00000102001	X	Rare Single Gene Mutation, Genetic Association

CACNA1G	calcium channel, voltage-dependent, T type, alpha 1G subunit	ENSG00000006283	17	Rare Single Gene Mutation, Syndromic, Genetic Association
BRINP3	BMP/retinoic acid inducible neural specific 3	ENSG00000162670	1	Rare Single Gene Mutation, Functional
CACNA1H	calcium channel, voltage-dependent, alpha 1H subunit	ENSG00000196557	16	Rare Single Gene Mutation
CACNA1I	Calcium channel, voltage-dependent, T type, alpha 1I subunit	ENSG00000100346	22	Rare Single Gene Mutation, Genetic Association
CACNA2D1	calcium voltage-gated channel auxiliary subunit alpha2delta 1	ENSG00000153956	7	Rare Single Gene Mutation
CACNA2D3	Calcium channel, voltage-dependent, alpha 2/delta subunit 3	ENSG00000157445	3	Rare Single Gene Mutation
CACNB2	Calcium channel, voltage-dependent, beta 2 subunit	ENSG00000165995	10	Rare Single Gene Mutation, Genetic Association
CADM1	cell adhesion molecule 1	ENSG00000182985	11	Rare Single Gene Mutation
CADM2	Cell adhesion molecule 2	ENSG00000175161	3	Rare Single Gene Mutation, Genetic Association
CADPS	calcium dependent secretion activator	ENSG00000163618	3	Rare Single Gene Mutation, Genetic Association
CADPS2	Ca2+-dependent activator protein for secretion 2	ENSG00000081803	7	Rare Single Gene Mutation, Functional
CAMK2A	calcium/calmodulin dependent protein kinase II alpha	ENSG00000070808	5	Rare Single Gene Mutation, Syndromic, Genetic Association, Functional
CAMK2B	calcium/calmodulin dependent protein kinase II beta	ENSG00000058404	7	Rare Single Gene Mutation, Syndromic
CAPN12	Calpain 12	ENSG00000182472	19	Rare Single Gene Mutation

CAPRIN1	Cell cycle associated protein 1	ENSG00000135387	11	Rare Single Gene Mutation, Functional
CARD11	caspase recruitment domain family member	ENSG00000198286	7	Rare Single Gene Mutation
CASC4	cancer susceptibility candidate 4	ENSG00000166734	15	Rare Single Gene Mutation
CASK	calcium/calmodulin dependent serine protein kinase	ENSG00000147044	X	Rare Single Gene Mutation, Syndromic
CASZ1	castor zinc finger 1	ENSG00000130940	1	Rare Single Gene Mutation
CC2D1A	Coiled-coil and C2 domain containing 1A	ENSG00000132024	19	Rare Single Gene Mutation, Functional
CCDC88C	Coiled-coil domain containing 88C	ENSG00000015133	14	Rare Single Gene Mutation
CCDC91	coiled-coil domain containing 91	ENSG00000123106	12	Rare Single Gene Mutation
CCIN	calicin	ENSG00000185972	9	Rare Single Gene Mutation
CCNG1	cyclin G1	ENSG00000113328	5	Rare Single Gene Mutation
CCNK	cyclin K	ENSG00000090061	14	Rare Single Gene Mutation, Syndromic
CCSER1	coiled-coil serine rich protein 1	ENSG00000184305	4	Rare Single Gene Mutation
CCT4	Chaperonin containing TCP1, subunit 4 (delta)	ENSG00000115484	2	Rare Single Gene Mutation
CD276	CD276molecule	ENSG00000103855	15	Rare Single Gene Mutation
CD38	CD38 molecule	ENSG00000004468	4	Rare Single Gene Mutation, Genetic Association, Functional
CDC42BPB	CDC42 binding protein kinase beta (DMPK-like)	ENSG00000198752	14	Rare Single Gene Mutation, Syndromic
CDH10	cadherin 10, type 2 (T2-cadherin)	ENSG00000040731	5	Rare Single Gene Mutation, Genetic Association
CDH11	cadherin 11	ENSG00000140937	16	Rare Single Gene Mutation
CDH13	cadherin 13	ENSG00000140945	16	Rare Single Gene Mutation
CDH8	cadherin 8, type 2	ENSG00000150394	16	Rare Single Gene Mutation

CDH9	cadherin 9, type 2 (T1-cadherin)	ENSG00000113100	5	Rare Single Gene Mutation, Genetic Association
CDK13	cyclin dependent kinase 13	ENSG00000065883	7	Rare Single Gene Mutation, Syndromic
CACNB1	calcium voltage-gated channel auxiliary subunit beta 1	ENSG00000067191	17	Rare Single Gene Mutation
CDKL5	cyclin-dependent kinase-like 5	ENSG00000008086	X	Rare Single Gene Mutation, Syndromic
CDON	cell adhesion associated, oncogene regulated	ENSG00000064309	11	Rare Single Gene Mutation
CECR2	CECR2, histone acetyl-lysine reader	ENSG00000099954	22	Rare Single Gene Mutation
CELF4	CUGBP, Elav-like family member 4	ENSG00000101489	18	Rare Single Gene Mutation, Functional
CELF6	CUGBP, Elav-like family member 6	ENSG00000140488	15	Rare Single Gene Mutation, Genetic Association
CEP135	centrosomal protein 135	ENSG00000174799	4	Rare Single Gene Mutation, Syndromic
CEP290	Centrosomal protein 290kDa	ENSG00000198707	12	Rare Single Gene Mutation, Syndromic
CEP41	testis specific, 14	ENSG00000106477	7	Rare Single Gene Mutation, Syndromic
CGNL1	Cingulin-like 1	ENSG00000128849	15	Rare Single Gene Mutation
CHAMP1	chromosome alignment maintaining phosphoprotein 1	ENSG00000198824	13	Rare Single Gene Mutation, Syndromic
CHD1	chromodomain helicase DNA binding protein 1	ENSG00000153922	5	Rare Single Gene Mutation
CHD2	Chromodomain helicase DNA binding protein 2	ENSG00000173575	15	Rare Single Gene Mutation, Syndromic
CHD3	chromodomain helicase DNA binding protein 3	ENSG00000170004	17	Rare Single Gene Mutation, Syndromic
CHD7	chromodomain helicase DNA binding protein 7	ENSG00000171316	8	Rare Single Gene Mutation, Syndromic

CHD8	chromodomain helicase DNA binding protein 8	ENSG00000100888	14	Rare Single Gene Mutation, Syndromic, Functional
CHKB	Choline kinase beta	ENSG00000100288	22	Rare Single Gene Mutation, Syndromic
CHM	CHMRab escort protein	ENSG00000188419	X	Rare Single Gene Mutation
CHMP1A	charged multivesicular body protein 1A	ENSG00000131165	16	Rare Single Gene Mutation, Syndromic
CHRM3	cholinergic receptor muscarinic 3	ENSG00000133019	1	Rare Single Gene Mutation, Genetic Association
CHRNA7	cholinergic receptor, nicotinic, alpha 7	ENSG00000175344	15	Rare Single Gene Mutation
CHRNB3	cholinergic receptor nicotinic beta 3 subunit	ENSG00000147432	8	Rare Single Gene Mutation
CIB2	Calcium and integrin binding family member 2	ENSG00000136425	15	Rare Single Gene Mutation
CIC	capicua transcriptional repressor	ENSG00000079432	19	Rare Single Gene Mutation, Functional
CLASP1	cytoplasmic linker associated protein 1	ENSG00000074054	2	Rare Single Gene Mutation
CLCN4	chloride voltage-gated channel 4	ENSG00000073464	X	Rare Single Gene Mutation, Syndromic
CLN8	Ceroid-lipofuscinosis, neuronal 8 (epilepsy, progressive with mental retardation)	ENSG00000182372	8	Rare Single Gene Mutation, Syndromic
CLTCL1	clathrin, heavy chain-like 1	ENSG00000070371	22	Rare Single Gene Mutation
CMIP	c-Maf inducing protein	ENSG00000153815	16	Rare Single Gene Mutation, Genetic Association
CMPK2	cytidine/uridine monophosphate kinase 2	ENSG00000134326	2	Rare Single Gene Mutation
CNGB3	cyclic nucleotide gated channel beta 3	ENSG00000170289	8	Rare Single Gene Mutation

CNKSR2	connector enhancer of kinase suppressor of Ras 2	ENSG00000149970	X	Rare Single Gene Mutation, Syndromic
CNOT3	CCR4-NOT transcription complex subunit 3	ENSG00000088038	19	Rare Single Gene Mutation, Syndromic
CNR1	cannabinoid receptor 1 (brain)	ENSG00000118432	6	Rare Single Gene Mutation, Genetic Association
CNTN3	contactin 3	ENSG00000113805	3	Rare Single Gene Mutation
CNTN4	contactin 4	ENSG00000144619	3	Rare Single Gene Mutation, Syndromic, Genetic Association
CNTN5	Contactin 5	ENSG00000149972	11	Rare Single Gene Mutation, Genetic Association
CNTN6	Contactin 6	ENSG00000134115	3	Rare Single Gene Mutation, Genetic Association
CNTNAP2	contactin associated protein-like 2	ENSG00000174469	7	Rare Single Gene Mutation, Syndromic, Genetic Association
CNTNAP3	contactin associated protein-like 3	ENSG00000106714	9	Rare Single Gene Mutation, Functional
CNTNAP4	Contactin associated protein-like 4	ENSG00000152910	16	Rare Single Gene Mutation, Functional
CNTNAP5	contactin associated protein-like 5	ENSG00000155052	2	Rare Single Gene Mutation, Genetic Association
COL28A1	collagen type XXVIII alpha 1 chain	ENSG00000215018	7	Rare Single Gene Mutation
CPT2	carnitine palmitoyltransferase 2	ENSG00000157184	1	Rare Single Gene Mutation
CPZ	carboxypeptidase Z	ENSG00000109625	4	Rare Single Gene Mutation
CREBBP	CREB binding protein	ENSG00000005339	16	Rare Single Gene Mutation, Syndromic, Genetic Association
CNOT1	CCR4-NOT transcription complex subunit 1	ENSG00000125107	16	Rare Single Gene Mutation, Syndromic
CORO1A	coronin 1A	ENSG00000102879	16	Rare Single Gene Mutation
CSDE1	cold shock domain containing E1	ENSG00000009307	1	Rare Single Gene Mutation, Syndromic
CSMD1	CUB and Sushi multiple domains 1	ENSG00000183117	8	Rare Single Gene Mutation, Genetic Association
CSNK1E	casein kinase 1 epsilon	ENSG00000213923	22	Rare Single Gene Mutation, Functional

CSNK2A1	casein kinase 2 alpha 1	ENSG00000101266	20	Rare Single Gene Mutation, Syndromic
CTCF	CCCTC-binding factor	ENSG00000102974	16	Rare Single Gene Mutation, Syndromic, Functional
CTNNA3	catenin (cadherin-associated protein), alpha 3	ENSG00000183230	10	Rare Single Gene Mutation, Genetic Association
CTNNB1	catenin beta 1	ENSG00000168036	3	Rare Single Gene Mutation, Syndromic
CTNND2	Catenin (cadherin-associated protein), delta 2	ENSG00000169862	5	Rare Single Gene Mutation
CTTNBP2	cortactin binding protein	ENSG00000077063	7	Rare Single Gene Mutation, Syndromic, Functional
CUL3	Cullin 3	ENSG00000036257	2	Rare Single Gene Mutation, Genetic Association
CUL7	Cullin 7	ENSG00000044090	6	Rare Single Gene Mutation
CUX1	cut like homeobox 1	ENSG00000257923	7	Rare Single Gene Mutation, Functional
CUX2	cut like homeobox 2	ENSG00000111249	12	Rare Single Gene Mutation, Syndromic
CX3CR1	Chemokine (C-X3-C motif) receptor 1	ENSG00000168329	3	Rare Single Gene Mutation, Functional
CYFIP1	cytoplasmic FMR1 interacting protein 1	ENSG00000273749	15	Rare Single Gene Mutation, Genetic Association, Functional
CYLC2	cylicin, basic protein of sperm head cytoskeleton 2	ENSG00000155833	9	Rare Single Gene Mutation
CYP27A1	cytochrome P450 family 27 subfamily A member 1	ENSG00000135929	2	Rare Single Gene Mutation, Syndromic
DAGLA	diacylglycerol lipase alpha	ENSG00000134780	11	Rare Single Gene Mutation, Functional
DAPP1	Dual adaptor of phosphotyrosine and 3-phosphoinositides	ENSG00000070190	4	Rare Single Gene Mutation
DDX3X	DEAD (Asp-Glu-Ala-Asp) box helicase 3, X-linked	ENSG00000215301	X	Rare Single Gene Mutation, Syndromic
DDX53	DEAD (Asp-Glu-Ala-Asp) box polypeptide 53	ENSG00000184735	X	Rare Single Gene Mutation

DEAF1	DEAF1 transcription factor	ENSG00000177030	11	Rare Single Gene Mutation, Syndromic
DENR	density-regulated protein	ENSG00000139726	12	Rare Single Gene Mutation
DEPDC5	DEP domain containing 5	ENSG00000100150	22	Rare Single Gene Mutation, Syndromic
DHCR7	7-dehydrocholesterol reductase	ENSG00000172893	11	Rare Single Gene Mutation, Syndromic
DHX30	DExH-box helicase 30	ENSG00000132153	3	Rare Single Gene Mutation, Syndromic
DIP2A	DIP2 disco-interacting protein 2 homolog A (Drosophila)	ENSG00000160305	21	Rare Single Gene Mutation, Functional
DIP2C	disco interacting protein 2 homolog C	ENSG00000151240	10	Rare Single Gene Mutation
DISC1	disrupted in schizophrenia 1	ENSG00000162946	1	Rare Single Gene Mutation, Syndromic, Genetic Association
DIPK2A	divergent protein kinase domain 2A	ENSG00000181744	3	Rare Single Gene Mutation
DDHD2	DDHD domain containing 2	ENSG00000085788	8	Rare Single Gene Mutation, Genetic Association, Functional
DIXDC1	DIX domain containing 1	ENSG00000150764	11	Rare Single Gene Mutation, Functional
DLG1	discs large MAGUK scaffold protein 1	ENSG00000075711	3	Rare Single Gene Mutation
DLG2	discs large MAGUK scaffold protein 2	ENSG00000150672	11	Rare Single Gene Mutation
DLG4	discs large MAGUK scaffold protein 4	ENSG00000132535	17	Rare Single Gene Mutation, Syndromic, Functional
DLGAP1	DLG associated protein 1	ENSG00000170579	18	Rare Single Gene Mutation, Functional
DLGAP2	discs, large (Drosophila) homolog-associated protein 2	ENSG00000198010	8	Rare Single Gene Mutation
DLGAP3	DLG associated protein 3	ENSG00000116544	1	Rare Single Gene Mutation, Genetic Association, Functional
DLL1	delta like canonical Notch ligand 1	ENSG00000198719	6	Rare Single Gene Mutation, Syndromic
DLX3	distal-less homeobox 3	ENSG00000064195	17	Rare Single Gene Mutation

DLX6	distal-less homeobox 6	ENSG00000006377	7	Rare Single Gene Mutation
DMD	dystrophin (muscular dystrophy, Duchenne and Becker types)	ENSG00000198947	X	Rare Single Gene Mutation, Syndromic, Genetic Association
DMPK	dystrophia myotonica-protein kinase	ENSG00000104936	19	Rare Single Gene Mutation, Syndromic
DMWD	DM1 locus, WD repeat containing	ENSG00000185800	19	Rare Single Gene Mutation
DMXL2	Dmx-like 2	ENSG00000104093	15	Rare Single Gene Mutation
DNAH10	Dynein, axonemal, heavy chain 10	ENSG00000197653	12	Rare Single Gene Mutation
DNAH17	dynein axonemal heavy chain 17	ENSG00000187775	17	Rare Single Gene Mutation, Genetic Association
DNAH3	dynein axonemal heavy chain 3	ENSG00000158486	16	Rare Single Gene Mutation
DNER	Delta/notch-like EGF repeat containing	ENSG00000187957	2	Rare Single Gene Mutation, Genetic Association
DNMT3A	DNA (cytosine-5-)-methyltransferase 3 alpha	ENSG00000119772	2	Rare Single Gene Mutation, Syndromic, Genetic Association
DOCK1	Dedicator of cytokinesis 1	ENSG00000150760	10	Rare Single Gene Mutation
DOCK4	Dedicator of cytokinesis 4	ENSG00000128512	7	Rare Single Gene Mutation, Genetic Association, Functional
DOCK8	dedicator of cytokinesis 8	ENSG00000107099	9	Rare Single Gene Mutation
DPP10	Dipeptidyl-peptidase 10	ENSG00000175497	2	Rare Single Gene Mutation, Genetic Association
DPP3	dipeptidyl peptidase 3	ENSG00000254986	11	Rare Single Gene Mutation
DPP4	Dipeptidyl-peptidase 4	ENSG00000197635	2	Rare Single Gene Mutation, Genetic Association
DPP6	dipeptidyl-peptidase 6	ENSG00000130226	7	Rare Single Gene Mutation, Genetic Association, Functional
DPYD	dihydropyrimidine dehydrogenase	ENSG00000188641	1	Rare Single Gene Mutation, Syndromic, Genetic Association
DPYSL2	dihydropyrimidinase like 2	ENSG00000092964	8	Rare Single Gene Mutation, Genetic Association, Functional

DPYSL3	dihydropyrimidinase like 3	ENSG00000113657	5	Rare Single Gene Mutation
DRD2	Dopamine receptor D2	ENSG00000149295	11	Rare Single Gene Mutation, Genetic Association
DRD3	dopamine receptor D3	ENSG00000151577	3	Rare Single Gene Mutation, Genetic Association
DSCAM	Down syndrome cell adhesion molecule	ENSG00000171587	21	Rare Single Gene Mutation, Genetic Association
DST	Dystonin	ENSG00000151914	6	Rare Single Gene Mutation
DUSP15	dual specificity phosphatase 15	ENSG00000149599	20	Rare Single Gene Mutation, Genetic Association
DVL3	Dishevelled segment polarity protein 3	ENSG00000161202	3	Rare Single Gene Mutation, Functional
DYDC1	DPY30 domain containing 1	ENSG00000170788	10	Rare Single Gene Mutation
DYDC2	DPY30 domain containing 2	ENSG00000133665	10	
DYNC1H1	dynein cytoplasmic 1 heavy chain 1	ENSG00000197102	14	Rare Single Gene Mutation, Syndromic
DYRK1A	Dual-specificity tyrosine-(Y)-phosphorylation regulated kinase 1A	ENSG00000157540	21	Rare Single Gene Mutation, Syndromic, Functional
EBF3	early B-cell factor 3	ENSG00000108001	10	Rare Single Gene Mutation, Syndromic
EEF1A2	Eukaryotic translation elongation factor 1 alpha 2	ENSG00000101210	20	Rare Single Gene Mutation, Syndromic
EFR3A	EFR3 homolog A (S. cerevisiae)	ENSG00000132294	8	Rare Single Gene Mutation
EGR3	early growth response 3	ENSG00000179388	8	Rare Single Gene Mutation
EHMT1	Euchromatic histone-lysine N-methyltransferase 1	ENSG00000181090	9	Rare Single Gene Mutation, Syndromic, Functional
EIF3G	eukaryotic translation initiation factor 3 subunit G	ENSG00000130811	19	Rare Single Gene Mutation

EIF4E	eukaryotic translation initiation factor 4E	ENSG00000151247	4	Rare Single Gene Mutation, Genetic Association
EIF4G1	eukaryotic translation initiation factor 4 gamma 1	ENSG00000114867	3	Rare Single Gene Mutation, Functional
ELAVL2	ELAV like neuron-specific RNA binding protein 2	ENSG00000107105	9	Rare Single Gene Mutation, Genetic Association, Functional
ELAVL3	ELAV like neuron-specific RNA binding protein 3	ENSG00000196361	19	Rare Single Gene Mutation
ELP4	Elongator acetyltransferase complex subunit 4	ENSG00000109911	11	Rare Single Gene Mutation, Genetic Association
EN2	engrailed homolog 2	ENSG00000164778	7	Rare Single Gene Mutation, Genetic Association, Functional
EP300	E1A binding protein p300	ENSG00000100393	22	Rare Single Gene Mutation, Syndromic
EMSY	EMSY, BRCA2 interacting transcriptional repressor	ENSG00000158636	11	Rare Single Gene Mutation, Functional
EP400	E1A binding protein p400	ENSG00000183495	12	Rare Single Gene Mutation
EPC2	Enhancer of polycomb homolog 2 (Drosophila)	ENSG00000135999	2	Rare Single Gene Mutation
EPHB2	EPH receptor B2	ENSG00000133216	1	Rare Single Gene Mutation
EPPK1	epiplakin 1	ENSG00000261150	8	Rare Single Gene Mutation
ERMN	ermin	ENSG00000136541	2	Rare Single Gene Mutation
ESR2	estrogen receptor 2 (ER beta)	ENSG00000140009	14	Rare Single Gene Mutation, Syndromic, Genetic Association
ESRRB	estrogen-related receptor beta	ENSG00000119715	14	Rare Single Gene Mutation, Genetic Association
ETFB	Electron-transfer-flavoprotein, beta polypeptide	ENSG00000105379	19	Rare Single Gene Mutation

EXOC3	exocyst complex component 3	ENSG00000180104	5	Rare Single Gene Mutation
EXOC5	exocyst complex component 5	ENSG00000070367	14	Rare Single Gene Mutation
EXOC6	exocyst complex component 6	ENSG00000138190	10	Rare Single Gene Mutation
EXOC6B	exocyst complex component 6B	ENSG00000144036	2	Rare Single Gene Mutation
EXT1	Exostosin 1	ENSG00000182197	8	Rare Single Gene Mutation, Genetic Association
FABP5	fatty acid binding protein 5 (psoriasis-associated)	ENSG00000164687	8	Rare Single Gene Mutation, Functional
ERBIN	erbb2 interacting protein	ENSG00000112851	5	Rare Single Gene Mutation
FAM47A	family with sequence similarity 47 member A	ENSG00000185448	X	Rare Single Gene Mutation
FAM92B	Family with sequence similarity 92, member B	ENSG00000153789	16	Rare Single Gene Mutation
FAM98C	family with sequence similarity 98 member C	ENSG00000130244	19	Rare Single Gene Mutation
FAN1	FANCD2/FANCI-associated nuclease 1	ENSG00000198690	15	Rare Single Gene Mutation, Genetic Association
FAT1	FAT atypical cadherin 1	ENSG00000083857	4	Rare Single Gene Mutation
FBN1	Fibrillin 1	ENSG00000166147	15	Rare Single Gene Mutation
FBXO11	F-box protein 11	ENSG00000138081	2	Rare Single Gene Mutation, Syndromic
FBXO40	F-box protein 40	ENSG00000163833	3	Rare Single Gene Mutation, Genetic Association
FCRL6	Fc receptor like 6	ENSG00000181036	1	Rare Single Gene Mutation
FEZF2	FEZ family zinc finger 2	ENSG00000153266	3	Rare Single Gene Mutation, Genetic Association
FGA	Fibrinogen alpha chain	ENSG00000171560	4	Rare Single Gene Mutation, Genetic Association
FHIT	fragile histidine triad	ENSG00000189283	3	Rare Single Gene Mutation, Genetic Association
FMR1	fragile X mental retardation 1	ENSG00000102081	X	Rare Single Gene Mutation, Syndromic, Genetic Association, Functional
FOXG1	Forkhead box G1	ENSG00000176165	14	Rare Single Gene Mutation, Syndromic, Genetic Association
FOXP1	forkhead box P1	ENSG00000114861	3	Rare Single Gene Mutation, Syndromic, Genetic Association, Functional

ENPP1	ectonucleotide pyrophosphatase/phosphodiesterase 1	ENSG00000197594	6	Rare Single Gene Mutation
FGF14	fibroblast growth factor 14	ENSG00000102466	13	Rare Single Gene Mutation
FOXP2	forkhead box P2	ENSG00000128573	7	Rare Single Gene Mutation, Genetic Association
FRK	fyn-related kinase	ENSG00000111816	6	Rare Single Gene Mutation, Genetic Association
FRMPD4	FERM and PDZ domain containing 4	ENSG00000169933	X	Rare Single Gene Mutation, Syndromic
GABBR2	gamma-aminobutyric acid type B receptor subunit 2	ENSG00000136928	9	Rare Single Gene Mutation, Syndromic, Genetic Association, Functional
GABRA3	Gamma-aminobutyric acid (GABA) A receptor, alpha 3	ENSG00000011677	X	Rare Single Gene Mutation
GABRA4	gamma-aminobutyric acid (GABA) A receptor, alpha 4	ENSG00000109158	4	Rare Single Gene Mutation, Genetic Association, Functional
GABRB3	gamma-aminobutyric acid (GABA) A receptor, beta 3	ENSG00000166206	15	Rare Single Gene Mutation, Genetic Association
GALNT13	polypeptide N-acetylgalactosaminyltransferase 13	ENSG00000144278	2	Rare Single Gene Mutation
GALNT14	polypeptide N-acetylgalactosaminyltransferase 14	ENSG00000158089	2	Rare Single Gene Mutation, Genetic Association
GALNT8	polypeptide N-acetylgalactosaminyltransferase 8	ENSG00000130035	12	Rare Single Gene Mutation
GDA	guanine deaminase	ENSG00000119125	9	Rare Single Gene Mutation, Genetic Association
GGNBP2	gametogenetin binding protein 2	ENSG00000278311	17	Rare Single Gene Mutation

GIGYF1	GRB10 interacting GYF protein 1	ENSG00000146830	7	Rare Single Gene Mutation
GIGYF2	GRB10 interacting GYF protein 2	ENSG00000204120	2	Rare Single Gene Mutation
GLIS1	GLIS family zinc finger 1	ENSG00000174332	1	Rare Single Gene Mutation, Genetic Association
GLO1	glyoxalase I	ENSG00000124767	6	Rare Single Gene Mutation, Genetic Association
GLRA2	glycine receptor, alpha 2	ENSG00000101958	X	Rare Single Gene Mutation, Functional
GNAS	GNAS complex locus	ENSG00000087460	20	Rare Single Gene Mutation
GNB1L	guanine nucleotide binding protein (G protein), beta polypeptide 1-like	ENSG00000185838	22	Rare Single Gene Mutation, Genetic Association
GPC4	glypican 4	ENSG00000076716	X	Rare Single Gene Mutation
GPC6	glypican 6	ENSG00000183098	13	Rare Single Gene Mutation, Genetic Association
GPD2	glycerol-3-phosphate dehydrogenase 2	ENSG00000115159	2	Rare Single Gene Mutation, Genetic Association
GPHN	Gephyrin	ENSG00000171723	14	Rare Single Gene Mutation
GPR37	G protein-coupled receptor 37	ENSG00000170775	7	Rare Single Gene Mutation
GPR85	G protein-coupled receptor 85	ENSG00000164604	7	Rare Single Gene Mutation, Genetic Association
GRIA1	glutamate ionotropic receptor AMPA type subunit 1	ENSG00000155511	5	Rare Single Gene Mutation
GRIA2	glutamate ionotropic receptor AMPA type subunit 2	ENSG00000120251	4	Rare Single Gene Mutation
GRID1	Glutamate receptor, ionotropic, delta 1	ENSG00000182771	10	Rare Single Gene Mutation, Genetic Association
GRID2	glutamate receptor, ionotropic, delta 2	ENSG00000152208	4	Rare Single Gene Mutation, Syndromic, Genetic Association
GRID2IP	Grid2 interacting protein	ENSG00000215045	7	Rare Single Gene Mutation

GRIK2	glutamate ionotropic receptor kainate type subunit 2	ENSG00000164418	6	Rare Single Gene Mutation, Genetic Association
FXN	frataxin	ENSG00000165060	9	Rare Single Gene Mutation
GABRB2	gamma-aminobutyric acid type A receptor subunit beta2	ENSG00000145864	5	Rare Single Gene Mutation
GFAP	glial fibrillary acidic protein	ENSG00000131095	17	Rare Single Gene Mutation
GNAI1	G protein subunit alpha i1	ENSG00000127955	7	Rare Single Gene Mutation
GRIK3	glutamate ionotropic receptor kainate type subunit 3	ENSG00000163873	1	Rare Single Gene Mutation, Genetic Association
GRIK4	Glutamate receptor, ionotropic, kainate 4	ENSG00000149403	11	Rare Single Gene Mutation, Functional
GRIK5	Glutamate receptor, ionotropic, kainate 5	ENSG00000105737	19	Rare Single Gene Mutation
GRIN1	Glutamate receptor, ionotropic, N-methyl D-aspartate 1	ENSG00000176884	9	Rare Single Gene Mutation, Functional
GRIN2A	glutamate receptor, ionotropic, N-methyl D-aspartate 2A	ENSG00000183454	16	Rare Single Gene Mutation, Syndromic, Genetic Association
GRIN2B	glutamate receptor, inotropic, N-methyl D-apartate 2B	ENSG00000273079	12	Rare Single Gene Mutation, Syndromic, Genetic Association
GRIP1	glutamate receptor interacting protein 1	ENSG00000155974	12	Rare Single Gene Mutation
GRM5	glutamate metabotropic receptor 5	ENSG00000168959	11	Rare Single Gene Mutation, Genetic Association, Functional
GRM7	Glutamate receptor, metabotropic 7	ENSG00000196277	3	Rare Single Gene Mutation, Genetic Association

GTF2I	general transcription factor IIi	ENSG00000263001	7	Rare Single Gene Mutation, Genetic Association
GUCY1A2	guanylate cyclase 1 soluble subunit alpha 2	ENSG00000152402	11	Rare Single Gene Mutation, Genetic Association
HCFC1	host cell factor C1	ENSG00000172534	X	Rare Single Gene Mutation, Syndromic
HCN1	Hyperpolarization activated cyclic nucleotide-gated potassium channel 1	ENSG00000164588	5	Rare Single Gene Mutation, Genetic Association
H2BC11	H2B clustered histone 11	ENSG00000124635	6	Rare Single Gene Mutation
HDAC4	histone deacetylase 4	ENSG00000068024	2	Rare Single Gene Mutation, Syndromic, Genetic Association
HDLBP	high density lipoprotein binding protein	ENSG00000115677	2	Rare Single Gene Mutation
HECTD4	HECT domain E3 ubiquitin protein ligase 4	ENSG00000173064	12	Rare Single Gene Mutation
HECW2	HECT, C2 and WW domain containing E3 ubiquitin protein ligase 2	ENSG00000138411	2	Rare Single Gene Mutation, Syndromic
HEPACAM	hepatic and glial cell adhesion molecule	ENSG00000165478	11	Rare Single Gene Mutation, Syndromic
HERC2	HECT and RLD domain containing E3 ubiquitin protein ligase 2	ENSG00000128731	15	Rare Single Gene Mutation
HIVEP3	human immunodeficiency virus type I enhancer binding protein 3	ENSG00000127124	1	Rare Single Gene Mutation, Genetic Association
HLA-DPB1	major histocompatibility complex, class II, DP beta 1	ENSG00000223865	6	Rare Single Gene Mutation, Genetic Association
HNRNPH2	heterogeneous nuclear ribonucleoprotein H2	ENSG00000126945	X	Rare Single Gene Mutation, Syndromic
HNRNPU	heterogeneous nuclear ribonucleoprotein U	ENSG00000153187	1	Rare Single Gene Mutation, Syndromic, Functional

HOMER1	Homer homolog 1 (Drosophila)	ENSG00000152413	5	Rare Single Gene Mutation, Functional
HOXA1	homeobox A1	ENSG00000105991	7	Rare Single Gene Mutation, Syndromic, Genetic Association
HRAS	v-Ha-ras Harvey rat sarcoma viral oncogene homolog	ENSG00000174775	11	Rare Single Gene Mutation, Syndromic, Genetic Association
HTR1B	5-hydroxytryptamine (serotonin) receptor 1B	ENSG00000135312	6	Rare Single Gene Mutation, Genetic Association
HTR3A	5-hydroxytryptamine (serotonin) receptor 3A	ENSG00000166736	11	Rare Single Gene Mutation, Genetic Association, Functional
HTR3C	5-hydroxytryptamine (serotonin) receptor 3, family member C	ENSG00000178084	3	Rare Single Gene Mutation, Genetic Association
HUWE1	HECT, UBA and WWE domain containing 1, E3 ubiquitin protein ligase	ENSG00000086758	X	Rare Single Gene Mutation, Syndromic
HYDIN	HYDIN, axonemal central pair apparatus	ENSG00000157423	16	Rare Single Gene Mutation, Genetic Association
ICA1	islet cell autoantigen 1	ENSG00000003147	7	Rare Single Gene Mutation
IL1R2	interleukin 1 receptor, type II	ENSG00000115590	2	Rare Single Gene Mutation
IL1RAPL1	interleukin 1 receptor accessory protein-like 1	ENSG00000169306	X	Rare Single Gene Mutation
IL1RAPL2	interleukin 1 receptor accessory protein-like 2	ENSG00000189108	X	Rare Single Gene Mutation, Genetic Association
ILF2	Interleukin enhancer binding factor 2	ENSG00000143621	1	Rare Single Gene Mutation
IMMP2L	IMP2 inner mitochondrial membrane peptidase-like (S. cerevisiae)	ENSG00000184903	7	Rare Single Gene Mutation, Genetic Association, Functional
INPP1	inositol polyphosphate-1-phosphatase	ENSG00000151689	2	Rare Single Gene Mutation, Genetic Association

INTS1	integrator complex subunit 1	ENSG00000164880	7	Rare Single Gene Mutation, Syndromic
INTS6	Integrator complex subunit 6	ENSG00000102786	13	Rare Single Gene Mutation
IQGAP3	IQ motif containing GTPase activating protein 3	ENSG00000183856	1	Rare Single Gene Mutation
IQSEC2	IQ motif and Sec7 domain 2	ENSG00000124313	X	Rare Single Gene Mutation, Syndromic, Functional
IRF2BPL	Interferon regulatory factor 2 binding protein-like	ENSG00000119669	14	Rare Single Gene Mutation, Syndromic
ITGB3	integrin, beta 3 (platelet glycoprotein IIIa, antigen CD61)	ENSG00000259207	17	Rare Single Gene Mutation, Genetic Association
ITPR1	inositol 1,4,5-trisphosphate receptor type 1	ENSG00000150995	3	Rare Single Gene Mutation, Genetic Association
ITSN1	intersectin 1	ENSG00000205726	21	Rare Single Gene Mutation
JARID2	jumonji and AT-rich interaction domain containing 2	ENSG00000008083	6	Rare Single Gene Mutation, Syndromic, Genetic Association
JMJD1C	jumonji domain containing 1C	ENSG00000171988	10	Rare Single Gene Mutation
KANK1	KN motif and ankyrin repeat domains 1	ENSG00000107104	9	Rare Single Gene Mutation
KANSL1	KAT8 regulatory NSL complex subunit 1	ENSG00000120071	17	Rare Single Gene Mutation, Syndromic
KAT2B	K(lysine) acetyltransferase 2B	ENSG00000114166	3	Rare Single Gene Mutation
KAT6A	K(lysine) acetyltransferase 6A	ENSG00000083168	8	Rare Single Gene Mutation, Syndromic

KATNAL1	katanin catalytic subunit A1 like 1	ENSG00000102781	13	Rare Single Gene Mutation, Functional
KATNAL2	Katanin p60 subunit A-like 2	ENSG00000167216	18	Rare Single Gene Mutation
KCNB1	potassium voltage-gated channel subfamily B member 1	ENSG00000158445	20	Rare Single Gene Mutation, Syndromic
KCNC1	potassium voltage-gated channel subfamily C member 1	ENSG00000129159	11	Rare Single Gene Mutation
KCND2	potassium voltage-gated channel subfamily D member 2	ENSG00000184408	7	Rare Single Gene Mutation, Genetic Association
KCND3	potassium voltage-gated channel subfamily D member 3	ENSG00000171385	1	Rare Single Gene Mutation, Syndromic, Genetic Association
KCNJ10	potassium voltage-gated channel subfamily J member 10	ENSG00000177807	1	Rare Single Gene Mutation, Syndromic, Genetic Association
KCNJ15	potassium voltage-gated channel subfamily J member 15	ENSG00000157551	21	Rare Single Gene Mutation
KCNK7	potassium two pore domain channel subfamily K member 7	ENSG00000173338	11	Rare Single Gene Mutation
KCNMA1	potassium large conductance calcium-activated channel, subfamily M, alpha member 1	ENSG00000156113	10	Rare Single Gene Mutation, Syndromic
IGF1	insulin like growth factor 1	ENSG00000017427	12	Rare Single Gene Mutation, Functional

KCNQ2	potassium voltage-gated channel subfamily Q member 2	ENSG00000075043	20	Rare Single Gene Mutation, Syndromic, Genetic Association
KCNQ3	potassium voltage-gated channel subfamily Q member 3	ENSG00000184156	8	Rare Single Gene Mutation, Genetic Association
KCNS3	potassium voltage-gated channel modifier subfamily S member 3	ENSG00000170745	2	Rare Single Gene Mutation
KCTD13	Potassium channel tetramerisation domain containing 13	ENSG00000174943	16	Rare Single Gene Mutation, Functional
KDM1B	lysine demethylase 1B	ENSG00000165097	6	Rare Single Gene Mutation
KDM4B	lysine demethylase 4B	ENSG00000127663	19	Rare Single Gene Mutation, Functional
KDM4C	lysine demethylase 4C	ENSG00000107077	9	Rare Single Gene Mutation
KDM5B	Lysine (K)-specific demethylase 5B	ENSG00000117139	1	Rare Single Gene Mutation, Syndromic, Functional
KDM5C	lysine demethylase 5C	ENSG00000126012	X	Rare Single Gene Mutation, Syndromic, Functional
KDM6A	lysine demethylase 6A	ENSG00000147050	X	Rare Single Gene Mutation, Syndromic
KDM6B	Lysine (K)-specific demethylase 6B	ENSG00000132510	17	Rare Single Gene Mutation, Syndromic
KHDRBS2	KH domain containing, RNA binding, signal transduction associated 2	ENSG00000112232	6	Rare Single Gene Mutation
KIAA1586	KIAA1586	ENSG00000168116	6	Rare Single Gene Mutation
KIF13B	Kinesin family member 13B	ENSG00000197892	8	Rare Single Gene Mutation
KIF14	kinesin family member 14	ENSG00000118193	1	Rare Single Gene Mutation, Syndromic
KIF5C	Kinesin family member 5C	ENSG00000168280	2	Rare Single Gene Mutation, Syndromic
KIRREL3	Kin of IRRE like 3 (Drosophila)	ENSG00000149571	11	Rare Single Gene Mutation
KLF16	Kruppel like factor 16	ENSG00000129911	19	Rare Single Gene Mutation

KMT2A	Lysine (K)-specific methyltransferase 2A	ENSG00000118058	11	Rare Single Gene Mutation, Syndromic
KMT2C	Lysine (K)-specific methyltransferase 2C	ENSG00000055609	7	Rare Single Gene Mutation, Syndromic, Functional
KMT2E	Lysine (K)-specific methyltransferase 2E	ENSG00000005483	7	Rare Single Gene Mutation, Syndromic, Genetic Association
KMT5B	lysine methyltransferase 5B	ENSG00000110066	11	Rare Single Gene Mutation, Syndromic
KIAA0232	KIAA0232	ENSG00000170871	4	Rare Single Gene Mutation
KNG1	kininogen 1	ENSG00000113889	3	Rare Single Gene Mutation
KPTN	kaptin, actin binding protein	ENSG00000118162	19	Rare Single Gene Mutation, Syndromic
KRR1	KRR1, small subunit (SSU) processome component, homolog (yeast)	ENSG00000111615	12	Rare Single Gene Mutation, Genetic Association
KRT26	keratin 26	ENSG00000186393	17	Rare Single Gene Mutation
LAMA1	Laminin, alpha 1	ENSG00000101680	18	Rare Single Gene Mutation, Genetic Association
LAMB1	laminin, beta 1	ENSG00000091136	7	Rare Single Gene Mutation, Genetic Association
LAS1L	LAS1 like ribosome biogenesis factor	ENSG00000001497	X	Rare Single Gene Mutation, Syndromic
LDB1	LIM domain binding 1	ENSG00000198728	10	Rare Single Gene Mutation
LDLR	low density lipoprotein receptor	ENSG00000130164	19	Rare Single Gene Mutation
LEO1	LEO1 homolog, Paf1/RNA polymerase II complex component	ENSG00000166477	15	Rare Single Gene Mutation
LEP	Leptin	ENSG00000174697	7	Rare Single Gene Mutation
LILRB2	leukocyte immunoglobulin like receptor B2	ENSG00000131042	19	Rare Single Gene Mutation

LIN7B	lin-7 homolog B, crumbs cell polarity complex component	ENSG00000104863	19	Rare Single Gene Mutation
LRBA	LPS-responsive vesicle trafficking, beach and anchor containing	ENSG00000198589	4	Rare Single Gene Mutation
LRFN2	leucine rich repeat and fibronectin type III domain containing 2	ENSG00000156564	6	Rare Single Gene Mutation, Genetic Association, Functional
LRFN5	leucine rich repeat and fibronectin type III domain containing 5	ENSG00000165379	14	Rare Single Gene Mutation, Genetic Association
LRP1	LDL receptor related protein 1	ENSG00000123384	12	Rare Single Gene Mutation
LRP2	LDL receptor related protein 2	ENSG00000081479	2	Rare Single Gene Mutation, Syndromic, Genetic Association
LRRC1	leucine rich repeat containing 1	ENSG00000137269	6	Rare Single Gene Mutation, Genetic Association
LRRC4	leucine rich repeat containing 4	ENSG00000128594	7	Rare Single Gene Mutation, Functional
LZTR1	Leucine-zipper-like transcription regulator 1	ENSG00000099949	22	Rare Single Gene Mutation, Syndromic
MACROD2	MACRO domain containing 2	ENSG00000172264	20	Rare Single Gene Mutation, Genetic Association
MAGEL2	MAGE-like 2	ENSG00000254585	15	Rare Single Gene Mutation, Syndromic
MAOA	monoamine oxidase A	ENSG00000189221	X	Rare Single Gene Mutation, Syndromic, Genetic Association, Functional
MAOB	monoamine oxidase B	ENSG00000069535	X	Rare Single Gene Mutation, Genetic Association, Functional
MAPK3	mitogen-activated protein kinase 3	ENSG00000102882	16	Rare Single Gene Mutation, Functional
MARK1	microtubule affinity regulating kinase 1	ENSG00000116141	1	Rare Single Gene Mutation, Genetic Association

MBD1	methyl-CpG binding domain protein 1	ENSG00000141644	18	Rare Single Gene Mutation
MBD3	methyl-CpG binding domain protein 3	ENSG00000071655	19	Rare Single Gene Mutation
MBD4	methyl-CpG binding domain protein 4	ENSG00000129071	3	Rare Single Gene Mutation
MBD5	Methyl-CpG binding domain protein 5	ENSG00000204406	2	Rare Single Gene Mutation, Syndromic, Functional
MBD6	Methyl-CpG binding domain protein 6	ENSG00000166987	12	Rare Single Gene Mutation
MBOAT7	membrane bound O-acyltransferase domain containing 7	ENSG00000125505	19	Rare Single Gene Mutation, Syndromic
MCM4	minichromosome maintenance complex component 4	ENSG00000104738	8	Rare Single Gene Mutation
MCM6	minichromosome maintenance complex component 6	ENSG00000076003	2	Rare Single Gene Mutation
MCPH1	microcephalin 1	ENSG00000147316	8	Rare Single Gene Mutation
MDGA2	MAM domain containing glycosylphosphatidylinositol anchor 2	ENSG00000139915	14	Rare Single Gene Mutation, Genetic Association
MECP2	Methyl CpG binding protein 2	ENSG00000169057	X	Rare Single Gene Mutation, Syndromic, Functional
LRRC4C	leucine rich repeat containing 4C	ENSG00000148948	11	Rare Single Gene Mutation
MAP1A	microtubule associated protein 1A	ENSG00000166963	15	Rare Single Gene Mutation
MED12L	mediator complex subunit 12L	ENSG00000144893	3	Rare Single Gene Mutation, Syndromic
MED13	mediator complex subunit 13	ENSG00000108510	17	Rare Single Gene Mutation, Syndromic

MED13L	Mediator complex subunit 13-like	ENSG00000123066	12	Rare Single Gene Mutation, Syndromic
MEF2C	myocyte enhancer factor 2C	ENSG00000081189	5	Rare Single Gene Mutation, Syndromic, Genetic Association, Functional
MEGF10	multiple EGF like domains 10	ENSG00000145794	5	Rare Single Gene Mutation, Genetic Association
MEGF11	multiple EGF like domains 11	ENSG00000157890	15	Rare Single Gene Mutation
MEIS2	Meis homeobox 2	ENSG00000134138	15	Rare Single Gene Mutation, Syndromic
MEMO1	mediator of cell motility 1	ENSG00000162959	2	Rare Single Gene Mutation, Functional
MET	met proto-oncogene (hepatocyte growth factor receptor)	ENSG00000105976	7	Rare Single Gene Mutation, Genetic Association, Functional
MFRP	Membrane frizzled-related protein	ENSG00000235718	11	Rare Single Gene Mutation
MIB1	Mindbomb E3 ubiquitin protein ligase 1	ENSG00000101752	18	Rare Single Gene Mutation
MLANA	melan-A	ENSG00000120215	9	Rare Single Gene Mutation
MPP6	membrane palmitoylated protein 6	ENSG00000105926	7	Rare Single Gene Mutation
MSANTD2	Myb/SANT DNA binding domain containing 2	ENSG00000120458	11	Rare Single Gene Mutation
METTL26	methyltransferase like 26	ENSG00000130731	16	Rare Single Gene Mutation
MIR137	microRNA 137	ENSG00000284202	1	Rare Single Gene Mutation, Syndromic, Genetic Association, Functional
MRTFB	myocardin related transcription factor B	ENSG00000186260	16	Rare Single Gene Mutation, Genetic Association
MSR1	macrophage scavenger receptor 1	ENSG00000038945	8	Rare Single Gene Mutation
MTHFR	methylenetetrahydrofolate reductase (NAD(P)H)	ENSG00000177000	1	Rare Single Gene Mutation, Syndromic, Genetic Association

MTOR	Mechanistic target of rapamycin (serine/threonine kinase)	ENSG00000198793	1	Rare Single Gene Mutation, Syndromic, Functional
MUC12	mucin 12, cell surface associated	ENSG00000205277	7	Rare Single Gene Mutation
MUC4	mucin 4, cell surface associated	ENSG00000145113	3	Rare Single Gene Mutation
MYH10	myosin heavy chain 10	ENSG00000133026	17	Rare Single Gene Mutation
MYH4	Myosin, heavy chain 4, skeletal muscle	ENSG00000264424	17	Rare Single Gene Mutation
MYO16	myosin XVI	ENSG00000041515	13	Rare Single Gene Mutation, Genetic Association
MYO1E	myosin IE	ENSG00000157483	15	Rare Single Gene Mutation
MYO5A	myosin VA	ENSG00000197535	15	Rare Single Gene Mutation, Genetic Association
MYO5C	myosin VC	ENSG00000128833	15	Rare Single Gene Mutation
MYO9B	Myosin IXB	ENSG00000099331	19	Rare Single Gene Mutation
MYT1L	Myelin transcription factor 1-like	ENSG00000186487	2	Rare Single Gene Mutation, Syndromic, Genetic Association
NAA15	N(alpha)-acetyltransferase 15, NatA auxiliary subunit	ENSG00000164134	4	Rare Single Gene Mutation, Syndromic
MKX	mohawk homeobox	ENSG00000150051	10	Rare Single Gene Mutation
MSX2	msh homeobox 2	ENSG00000120149	5	Rare Single Gene Mutation, Syndromic
MYLK	myosin light chain kinase	ENSG00000065534	3	Rare Single Gene Mutation
MYOCD	myocardin	ENSG00000141052	17	Rare Single Gene Mutation
NAALADL2	N-acetylated alpha-linked acidic dipeptidase-like 2	ENSG00000177694	3	Rare Single Gene Mutation, Genetic Association
NACC1	nucleus accumbens associated 1	ENSG00000160877	19	Rare Single Gene Mutation, Syndromic
NAV2	neuron navigator 2	ENSG00000166833	11	Rare Single Gene Mutation
NBEA	neurobeachin	ENSG00000172915	13	Rare Single Gene Mutation, Syndromic, Functional
NCKAP1	NCK-associated protein 1	ENSG00000061676	2	Rare Single Gene Mutation
NCKAP5	NCK-associated protein 5	ENSG00000176771	2	Rare Single Gene Mutation

NCOR1	nuclear receptor corepressor 1	ENSG00000141027	17	Rare Single Gene Mutation, Functional
NEGR1	neuronal growth regulator 1	ENSG00000172260	1	Rare Single Gene Mutation, Genetic Association, Functional
NEO1	Neogenin 1	ENSG00000067141	15	
NF1	neurofibromin 1 (neurofibromatosis, von Recklinghausen disease, Watson disease)	ENSG00000196712	17	Rare Single Gene Mutation, Syndromic, Genetic Association
NFE2L3	nuclear factor, erythroid 2 like 3	ENSG00000050344	7	Rare Single Gene Mutation
NFIA	nuclear factor I/A	ENSG00000162599	1	Rare Single Gene Mutation
NFIB	nuclear factor I B	ENSG00000147862	9	Rare Single Gene Mutation, Syndromic
NFIX	nuclear factor I/X (CCAAT-binding transcription factor)	ENSG00000008441	19	Rare Single Gene Mutation, Syndromic
NINL	Ninein-like	ENSG00000101004	20	Rare Single Gene Mutation
NIPA1	non imprinted in Prader-Willi/Angelman syndrome 1	ENSG00000170113	15	Rare Single Gene Mutation
NIPA2	non imprinted in Prader-Willi/Angelman syndrome 2	ENSG00000140157	15	Rare Single Gene Mutation
NIPBL	Nipped-B homolog (Drosophila)	ENSG00000164190	5	Rare Single Gene Mutation, Syndromic
NEXMIF	neurite extension and migration factor	ENSG00000050030	X	Rare Single Gene Mutation, Syndromic, Functional
NLGN1	neuroligin 1	ENSG00000169760	3	Rare Single Gene Mutation, Genetic Association
NLGN2	Neuroligin 2	ENSG00000169992	17	Rare Single Gene Mutation, Functional
NLGN3	neuroligin 3	ENSG00000196338	X	Rare Single Gene Mutation
NLGN4X	neuroligin 4, X-linked	ENSG00000146938	X	Rare Single Gene Mutation, Genetic Association
NLGN4Y	neuroligin 4, Y-linked	ENSG00000165246	Y	Rare Single Gene Mutation, Genetic Association, Functional

NR1D1	nuclear receptor subfamily 1 group D member 1	ENSG00000126368	17	Rare Single Gene Mutation
NR2F1	nuclear receptor subfamily 2 group F member 1	ENSG00000175745	5	Rare Single Gene Mutation, Syndromic, Functional
NR3C2	Nuclear receptor subfamily 3, group C, member 2	ENSG00000151623	4	Rare Single Gene Mutation, Syndromic
NR4A2	nuclear receptor subfamily 4 group A member 2	ENSG00000153234	2	Rare Single Gene Mutation, Syndromic
NRCAM	neuronal cell adhesion molecule	ENSG00000091129	7	Rare Single Gene Mutation, Genetic Association
NRP2	neuropilin 2	ENSG00000118257	2	Rare Single Gene Mutation, Genetic Association
NRXN1	neurexin 1	ENSG00000179915	2	Rare Single Gene Mutation, Syndromic, Genetic Association, Functional
NCAPH2	non-SMC condensin II complex subunit H2	ENSG00000025770	22	Rare Single Gene Mutation
NCOA1	nuclear receptor coactivator 1	ENSG00000084676	2	Rare Single Gene Mutation
NRXN2	neurexin 2	ENSG00000110076	11	Rare Single Gene Mutation, Genetic Association
NRXN3	neurexin 3	ENSG00000021645	14	Rare Single Gene Mutation, Genetic Association
NSD1	nuclear receptor binding SET domain protein 1	ENSG00000165671	5	Rare Single Gene Mutation, Syndromic
NTNG1	netrin G1	ENSG00000162631	1	Rare Single Gene Mutation, Syndromic, Genetic Association
NTRK1	neurotrophic tyrosine kinase, receptor, type 1	ENSG00000198400	1	Rare Single Gene Mutation, Syndromic
NTRK3	neurotrophic tyrosine kinase, receptor, type 3	ENSG00000140538	15	Rare Single Gene Mutation, Syndromic, Genetic Association
NUAK1	NUAK family, SNF1-like kinase, 1	ENSG00000074590	12	Rare Single Gene Mutation, Functional

NUDCD2	NudC domain containing 2	ENSG00000170584	5	Rare Single Gene Mutation
NUP133	nucleoporin 133kDa	ENSG00000069248	1	Rare Single Gene Mutation
NXPH1	neurexophilin 1	ENSG00000122584	7	Rare Single Gene Mutation
OCRL	oculocerebrorenal syndrome of Lowe	ENSG00000122126	X	Rare Single Gene Mutation, Syndromic
ODF3L2	outer dense fiber of sperm tails 3-like 2	ENSG00000181781	19	Rare Single Gene Mutation
OFD1	OFD1, centriole and centriolar satellite protein	ENSG00000046651	X	Rare Single Gene Mutation
OPHN1	oligophrenin 1	ENSG00000079482	X	Rare Single Gene Mutation, Syndromic
OR1C1	olfactory receptor, family 1, subfamily C, member 1	ENSG00000221888	1	Rare Single Gene Mutation
OR2T10	olfactory receptor family 2 subfamily T member 10	ENSG00000184022	1	Rare Single Gene Mutation
OR52M1	Olfactory receptor, family 52, subfamily M, member 1	ENSG00000197790	11	Rare Single Gene Mutation
OTUD7A	OTU deubiquitinase 7A	ENSG00000169918	15	Rare Single Gene Mutation, Functional
NUP155	nucleoporin 155	ENSG00000113569	5	Rare Single Gene Mutation
NXF1	nuclear RNA export factor 1	ENSG00000162231	11	Rare Single Gene Mutation
OXT	oxytocin/neurophysin I prepropeptide	ENSG00000101405	20	Rare Single Gene Mutation, Genetic Association
OXTR	oxytocin receptor	ENSG00000180914	3	Rare Single Gene Mutation, Genetic Association, Functional
P2RX5	Purinergic receptor P2X, ligand gated ion channel, 5	ENSG00000083454	17	Rare Single Gene Mutation
P4HA2	Prolyl 4-hydroxylase, alpha polypeptide II	ENSG00000072682	5	Rare Single Gene Mutation
PACS1	phosphofurin acidic cluster sorting protein 1	ENSG00000175115	11	Rare Single Gene Mutation, Syndromic

PACS2	phosphofurin acidic cluster sorting protein 2	ENSG00000179364	14	Rare Single Gene Mutation, Syndromic
PAFAH1B2	platelet activating factor acetylhydrolase 1b catalytic subunit 2	ENSG00000168092	11	Rare Single Gene Mutation
PAH	Phenylalanine hydroxylase	ENSG00000171759	12	Rare Single Gene Mutation, Syndromic
PAK1	p21 (RAC1) activated kinase 1	ENSG00000149269	11	Rare Single Gene Mutation, Syndromic
PAK2	p21 (RAC1) activated kinase 2	ENSG00000180370	3	Rare Single Gene Mutation
PAPOLG	poly(A) polymerase gamma	ENSG00000115421	2	Rare Single Gene Mutation
PARD3B	Par-3 partitioning defective 3 homolog B (C. elegans)	ENSG00000116117	2	Rare Single Gene Mutation, Genetic Association
PATJ	PATJ, crumbs cell polarity complex component	ENSG00000132849	1	Rare Single Gene Mutation, Genetic Association
PAX5	Paired box 5	ENSG00000196092	9	Rare Single Gene Mutation
PAX6	Paired box 6	ENSG00000007372	11	Rare Single Gene Mutation, Syndromic, Functional
PBX1	PBX homeobox 1	ENSG00000185630	1	Rare Single Gene Mutation
PCCA	propionyl-CoA carboxylase alpha subunit	ENSG00000175198	13	Rare Single Gene Mutation, Syndromic
PCCB	propionyl-CoA carboxylase beta subunit	ENSG00000114054	3	Rare Single Gene Mutation, Syndromic
PCDH10	protocadherin 10	ENSG00000138650	4	Rare Single Gene Mutation
PCDH11X	protocadherin 11 X-	ENSG00000102290	X	Rare Single Gene Mutation
PCDH15	protocadherin related 15	ENSG00000150275	10	Rare Single Gene Mutation, Syndromic, Genetic Association
PCDH19	protocadherin 19	ENSG00000165194	X	Rare Single Gene Mutation, Syndromic, Functional
PCDH9	protocadherin 9	ENSG00000184226	13	Rare Single Gene Mutation, Genetic Association
PCDHA1	Protocadherin alpha 1	ENSG00000204970	5	Rare Single Gene Mutation, Genetic Association
PCDHA10	Protocadherin alpha 10	ENSG00000250120	5	Rare Single Gene Mutation, Genetic Association

PCDHA11	Protocadherin alpha 11	ENSG00000249158	5	Rare Single Gene Mutation, Genetic Association
PCDHA12	Protocadherin alpha 12	ENSG00000251664	5	Rare Single Gene Mutation, Genetic Association
PCDHA13	Protocadherin alpha 13	ENSG00000239389	5	Rare Single Gene Mutation, Genetic Association
PCDHA2	Protocadherin alpha 2	ENSG00000204969	5	Rare Single Gene Mutation, Genetic Association
PCDHA3	Protocadherin alpha 3	ENSG00000255408	5	Rare Single Gene Mutation, Genetic Association
PCDHA4	Protocadherin alpha 4	ENSG00000204967	5	Rare Single Gene Mutation, Genetic Association
PCDHA5	Protocadherin alpha 5	ENSG00000204965	5	Rare Single Gene Mutation, Genetic Association
PCDHA6	Protocadherin alpha 6	ENSG00000081842	5	Rare Single Gene Mutation, Genetic Association
PCDHA7	Protocadherin alpha 7	ENSG00000204963	5	Rare Single Gene Mutation, Genetic Association
PCDHA8	Protocadherin alpha 8	ENSG00000204962	5	Rare Single Gene Mutation, Genetic Association
PCDHA9	Protocadherin alpha 9	ENSG00000204961	5	Rare Single Gene Mutation, Genetic Association
PCDHAC1	Protocadherin alpha subfamily C, 1	ENSG00000248383	5	Rare Single Gene Mutation, Genetic Association
PCM1	pericentriolar material 1	ENSG00000078674	8	Rare Single Gene Mutation
PDCD1	programmed cell death 1	ENSG00000188389	2	Rare Single Gene Mutation
PDE1C	phosphodiesterase 1C	ENSG00000154678	7	Rare Single Gene Mutation, Genetic Association
PDK2	pyruvate dehydrogenase kinase 2	ENSG00000005882	17	Rare Single Gene Mutation
PER1	period homolog 1 (Drosophila)	ENSG00000179094	17	Rare Single Gene Mutation, Genetic Association
PER2	period circadian clock 2	ENSG00000132326	2	Rare Single Gene Mutation
PEX7	peroxisomal biogenesis factor 7	ENSG00000112357	6	Rare Single Gene Mutation, Genetic Association
PHF2	PHD finger protein 2	ENSG00000197724	9	Rare Single Gene Mutation
PHF21A	PHD finger protein 21A	ENSG00000135365	11	Rare Single Gene Mutation, Syndromic
PHF3	PHD finger protein 3	ENSG00000118482	6	Rare Single Gene Mutation
PHF8	PHD finger protein 8	ENSG00000172943	X	Rare Single Gene Mutation, Syndromic
PHIP	pleckstrin homology domain interacting	ENSG00000146247	6	Rare Single Gene Mutation, Syndromic
PEBP4	phosphatidylethanolamine binding protein 4	ENSG00000134020	8	Rare Single Gene Mutation
PHF12	PHD finger protein 12	ENSG00000109118	17	Rare Single Gene Mutation

PHRF1	PHD and ring finger domains 1	ENSG00000070047	11	Rare Single Gene Mutation
PIK3R2	phosphoinositide-3-kinase regulatory subunit	ENSG00000105647	19	Rare Single Gene Mutation, Syndromic
PITX1	paired-like homeodomain 1	ENSG00000069011	5	Rare Single Gene Mutation, Genetic Association
PLAUR	Plasminogen activator, urokinase receptor	ENSG00000011422	19	Rare Single Gene Mutation, Genetic Association
PLCB1	phospholipase C, beta 1 (phosphoinositide-specific)	ENSG00000182621	20	Rare Single Gene Mutation
PLCD4	phospholipase C delta 4	ENSG00000115556	2	Rare Single Gene Mutation
PLN	phospholamban	ENSG00000198523	6	Rare Single Gene Mutation
PLXNA3	plexin A3	ENSG00000130827	X	Rare Single Gene Mutation
PLXNA4	Plexin A4	ENSG00000221866	7	Rare Single Gene Mutation, Functional
PLXNB1	plexin B1	ENSG00000164050	3	Rare Single Gene Mutation
PNPLA7	patatin like phospholipase domain containing 7	ENSG00000130653	9	Rare Single Gene Mutation
POGZ	Pogo transposable element with ZNF	ENSG00000143442	1	Rare Single Gene Mutation, Syndromic, Functional
POLA2	DNA polymerase alpha 2, accessory subunit	ENSG00000014138	11	Rare Single Gene Mutation
POMGNT1	protein O-linked mannose N-acetylglucosaminyltransferase 1 (beta 1,2-)	ENSG00000085998	1	Rare Single Gene Mutation, Syndromic
POMT1	protein O-mannosyltransferase 1	ENSG00000130714	9	Rare Single Gene Mutation
POT1	Protection of telomeres 1 homolog (S. pombe)	ENSG00000128513	7	Rare Single Gene Mutation
PPFIA1	PTPRF interacting protein alpha 1	ENSG00000131626	11	Rare Single Gene Mutation

PPM1D	protein phosphatase, Mg2+/Mn2+ dependent 1D	ENSG00000170836	17	Rare Single Gene Mutation, Syndromic
PPP2R1B	protein phosphatase 2 regulatory subunit A, beta	ENSG00000137713	11	Rare Single Gene Mutation
PPP2R5D	Protein phosphatase 2, regulatory subunit B', delta	ENSG00000112640	6	Rare Single Gene Mutation, Syndromic
PREX1	Phosphatidylinositol-3,4,5-trisphosphate-dependent Rac exchange factor 1	ENSG00000124126	20	Rare Single Gene Mutation, Genetic Association
PRICKLE1	Prickle homolog 1 (Drosophila)	ENSG00000139174	12	Rare Single Gene Mutation, Syndromic, Functional
PRICKLE2	prickle planar cell polarity protein 2	ENSG00000163637	3	Rare Single Gene Mutation, Functional
PRKAR1B	protein kinase cAMP-dependent type I regulatory subunit beta	ENSG00000188191	7	Rare Single Gene Mutation
PRKCA	protein kinase C alpha	ENSG00000154229	17	Rare Single Gene Mutation
PRKCB	protein kinase C beta	ENSG00000166501	16	Rare Single Gene Mutation, Genetic Association
PRKD1	Protein kinase D1	ENSG00000184304	14	Rare Single Gene Mutation, Syndromic
PRKD2	protein kinase D2	ENSG00000105287	19	Rare Single Gene Mutation
PRKDC	protein kinase, DNA-activated, catalytic polypeptide	ENSG00000253729	8	Rare Single Gene Mutation, Syndromic, Functional
PRKN	parkin RBR E3 ubiquitin protein ligase	ENSG00000185345	6	Rare Single Gene Mutation, Genetic Association
PRPF39	pre-mRNA processing factor 39	ENSG00000185246	14	Rare Single Gene Mutation
PRR12	proline rich 12	ENSG00000126464	19	Rare Single Gene Mutation, Syndromic
PRUNE2	prune homolog 2	ENSG00000106772	9	Rare Single Gene Mutation, Syndromic

PSD3	pleckstrin and Sec7 domain containing 3	ENSG00000156011	8	Rare Single Gene Mutation, Genetic Association
PTBP2	polypyrimidine tract binding protein 2	ENSG00000117569	1	Rare Single Gene Mutation, Genetic Association
PTCHD1	patched domain containing 1	ENSG00000165186	X	Rare Single Gene Mutation, Genetic Association
PTEN	phosphatase and tensin homolog (mutated in multiple advanced cancers 1)	ENSG00000171862	10	Rare Single Gene Mutation, Syndromic, Functional
PTCHD1-AS	PTCHD1antisense RNA (head to head)	ENSG00000233067	X	Rare Single Gene Mutation
POLR3A	RNA polymerase III subunit A	ENSG00000148606	10	Rare Single Gene Mutation, Syndromic
PPP1R9B	protein phosphatase 1 regulatory subunit 9B	ENSG00000108819	17	Rare Single Gene Mutation
PPP5C	protein phosphatase 5 catalytic subunit	ENSG00000011485	19	Rare Single Gene Mutation
PTGS2	prostaglandin-endoperoxide synthase 2	ENSG00000073756	1	Rare Single Gene Mutation, Genetic Association, Functional
PTK7	Protein tyrosine kinase 7 (inactive)	ENSG00000112655	6	Rare Single Gene Mutation
PTPN11	protein tyrosine phosphatase, non-receptor type 11	ENSG00000179295	12	Rare Single Gene Mutation, Syndromic
PTPRB	protein tyrosine phosphatase, receptor type B	ENSG00000127329	12	Rare Single Gene Mutation, Genetic Association
PTPRC	protein tyrosine phosphatase, receptor type, C	ENSG00000081237	1	Rare Single Gene Mutation, Genetic Association

PTPRT	protein tyrosine phosphatase, receptor type, T	ENSG00000196090	20	Rare Single Gene Mutation, Functional
PXDN	peroxidasin	ENSG00000130508	2	Rare Single Gene Mutation
PYHIN1	Pyrin and HIN domain family, member 1	ENSG00000163564	1	Rare Single Gene Mutation
QRICH1	glutamine rich 1	ENSG00000198218	3	Rare Single Gene Mutation
RAB11FIP5	RAB11 family interacting protein 5	ENSG00000135631	2	Rare Single Gene Mutation
RAB2A	RAB2A, member RAS oncogene family	ENSG00000104388	8	Rare Single Gene Mutation
RAB39B	RAB39B, member RAS oncogene family	ENSG00000155961	X	Rare Single Gene Mutation, Syndromic, Functional
RAB43	RAB43, member RAS oncogene family	ENSG00000172780	3	Rare Single Gene Mutation
RAD21L1	RAD21 cohesin complex component like 1	ENSG00000244588	20	Rare Single Gene Mutation
RAI1	retinoic acid induced 1	ENSG00000108557	17	Rare Single Gene Mutation, Syndromic
RALGAPB	Ral GTPase activating protein non-catalytic beta subunit	ENSG00000170471	20	Rare Single Gene Mutation
RANBP17	RAN binding protein 17	ENSG00000204764	5	Rare Single Gene Mutation
RAPGEF4	Rap guanine nucleotide exchange factor (GEF) 4	ENSG00000091428	2	Rare Single Gene Mutation
RASSF5	Ras association domain family member 5	ENSG00000266094	1	Rare Single Gene Mutation, Genetic Association
RBFOX1	RNA binding protein, fox-1 homolog (C.	ENSG00000078328	16	Rare Single Gene Mutation, Genetic Association
RBM27	RNA binding motif protein 27	ENSG00000091009	5	Rare Single Gene Mutation
REEP3	receptor accessory protein 3	ENSG00000165476	10	Rare Single Gene Mutation

RELN	Reelin	ENSG00000189056	7	Rare Single Gene Mutation, Syndromic, Genetic Association, Functional
RERE	Arginine-glutamic acid dipeptide (RE) repeats	ENSG00000142599	1	Rare Single Gene Mutation, Syndromic, Genetic Association
RFX3	regulatory factor X3	ENSG00000080298	9	Rare Single Gene Mutation
RGS7	regulator of G-protein signaling 7	ENSG00000182901	1	Rare Single Gene Mutation
RIMS1	Regulating synaptic membrane exocytosis 1	ENSG00000079841	6	Rare Single Gene Mutation, Genetic Association
RIMS3	regulating synaptic membrane exocytosis 3	ENSG00000117016	1	Rare Single Gene Mutation
RLIM	Ring finger protein, LIM domain interacting	ENSG00000131263	X	Rare Single Gene Mutation, Syndromic
RNF135	Ring finger protein 135	ENSG00000181481	17	Rare Single Gene Mutation, Syndromic, Genetic Association
RNF25	ring finger protein 25	ENSG00000163481	2	Rare Single Gene Mutation
RNF38	ring finger protein 38	ENSG00000137075	9	Rare Single Gene Mutation
ROBO2	roundabout guidance receptor 2	ENSG00000185008	3	Rare Single Gene Mutation, Genetic Association, Functional
RORA	RAR-related orphan receptor A	ENSG00000069667	15	Rare Single Gene Mutation, Syndromic, Genetic Association, Functional
RORB	RAR related orphan receptor B	ENSG00000198963	9	Rare Single Gene Mutation, Syndromic, Functional
RPL10	ribosomal protein L10	ENSG00000147403	X	Rare Single Gene Mutation, Syndromic
RPS6KA2	ribosomal protein S6 kinase, 90kDa, polypeptide 2	ENSG00000071242	6	Rare Single Gene Mutation
RPS6KA3	Ribosomal protein S6 kinase, 90kDa, polypeptide 3	ENSG00000177189	X	Rare Single Gene Mutation, Syndromic
SAE1	SUMO1 activating enzyme subunit 1	ENSG00000142230	19	Rare Single Gene Mutation
SAMD11	sterile alpha motif domain containing 11	ENSG00000187634	1	Rare Single Gene Mutation

SASH1	SAM and SH3 domain containing 1	ENSG00000111961	6	Rare Single Gene Mutation
SATB2	SATB homeobox 2	ENSG00000119042	2	Rare Single Gene Mutation, Syndromic, Genetic Association
RP11-1407O15.2		ENSG00000182591	17	Rare Single Gene Mutation
RIMS2	regulating synaptic membrane exocytosis 2	ENSG00000176406	8	Rare Single Gene Mutation, Syndromic, Genetic Association
SATB1	SATB homeobox 1	ENSG00000182568	3	Rare Single Gene Mutation
SBF1	SET binding factor 1	ENSG00000100241	22	Rare Single Gene Mutation
SCFD2	sec1 family domain containing 2	ENSG00000184178	4	Rare Single Gene Mutation
SCN1A	sodium channel, voltage-gated, type I, alpha subunit	ENSG00000144285	2	Rare Single Gene Mutation, Syndromic, Genetic Association
SCN2A	sodium channel, voltage-gated, type II, alpha subunit	ENSG00000136531	2	Rare Single Gene Mutation, Syndromic
SCN4A	Sodium channel, voltage gated, type IV alpha subunit	ENSG00000007314	17	Rare Single Gene Mutation
SCN8A	sodium channel, voltage gated, type VIII, alpha subunit	ENSG00000196876	12	Rare Single Gene Mutation, Syndromic
SCN9A	sodium voltage-gated channel alpha subunit 9	ENSG00000169432	2	Rare Single Gene Mutation
SCP2	sterol carrier protein 2	ENSG00000116171	1	Rare Single Gene Mutation
SDC2	syndecan 2 (heparan sulfate proteoglycan 1, cell surface-associated, fibroglycan)	ENSG00000169439	8	Rare Single Gene Mutation, Genetic Association

SEMA5A	sema domain, seven thrombospondin repeats (type 1 and type 1-like), transmembrane domain (TM) and short cytoplasmic domain, (semaphorin) 5A	ENSG00000112902	5	Rare Single Gene Mutation, Genetic Association, Functional
SET	SETnuclear proto-oncogene	ENSG00000152217	9	Rare Single Gene Mutation
SETBP1	SET binding protein 1	ENSG00000152217	18	Rare Single Gene Mutation, Syndromic
SETD1B	SET domain containing 1B	ENSG00000139718	12	Rare Single Gene Mutation, Syndromic
SETD2	SET domain containing 2	ENSG00000181555	3	Rare Single Gene Mutation, Syndromic
SETD5	SET domain containing 5	ENSG00000168137	3	Rare Single Gene Mutation, Syndromic
SETDB1	SET domain, bifurcated 1	ENSG00000143379	1	Rare Single Gene Mutation
SETDB2	SET domain, bifurcated 2	ENSG00000136169	13	Rare Single Gene Mutation, Syndromic
SEZ6L2	SEZ6L2 seizure related 6 homolog (mouse)-like 2	ENSG00000174938	16	Rare Single Gene Mutation, Genetic Association
SF3B1	splicing factor 3b subunit 1	ENSG00000115524	2	Rare Single Gene Mutation
SGSH	N-sulfoglucosamine sulfohydrolase	ENSG00000181523	17	Rare Single Gene Mutation, Syndromic
SGSM3	Small G protein signaling modulator 3	ENSG00000100359	22	Rare Single Gene Mutation
SH3RF3	SH3 domain containing ring finger 3	ENSG00000172985	2	Rare Single Gene Mutation
SHANK1	SH3 and multiple ankyrin repeat domains 1	ENSG00000161681	19	Rare Single Gene Mutation, Genetic Association
SHANK2	SH3 and multiple ankyrin repeat domains 2	ENSG00000162105	11	Rare Single Gene Mutation, Syndromic, Genetic Association, Functional
SHANK3	SH3 and multiple ankyrin repeat domains 3	ENSG00000251322	22	Rare Single Gene Mutation, Syndromic, Genetic Association, Functional

SETD1A	SET domain containing 1A, histone lysine methyltransferase	ENSG00000099381	16	Rare Single Gene Mutation, Syndromic
SHOX	short stature homeobox	ENSG00000185960	X,Y	Rare Single Gene Mutation
SIK1	Salt-inducible kinase 1	ENSG00000142178	21	Rare Single Gene Mutation, Syndromic
SIN3A	SIN3 transcription regulator family member A	ENSG00000169375	15	Rare Single Gene Mutation, Syndromic
SLC12A5	Solute carrier family 12 (potassium/chloride transporter), member 5	ENSG00000124140	20	Rare Single Gene Mutation
SLC1A1	solute carrier family 1 (neuronal/epithelial high affinity glutamate transporter, system Xag), member 1	ENSG00000106688	9	Rare Single Gene Mutation, Genetic Association
SLC1A2	Solute carrier family 1 (glial high affinity glutamate transporter), member 2	ENSG00000110436	11	Rare Single Gene Mutation, Genetic Association, Functional
SLC22A9	solute carrier family 22 member 9	ENSG00000149742	11	Rare Single Gene Mutation
SLC24A2	solute carrier family 24 member 2	ENSG00000155886	9	Rare Single Gene Mutation
SLC25A12	solute carrier family 25 (mitochondrial carrier, Aralar), member 12	ENSG00000115840	2	Rare Single Gene Mutation, Genetic Association, Functional
SLC25A39	solute carrier family 25 member 39	ENSG00000013306	17	Rare Single Gene Mutation
SLC27A4	Solute carrier family 27 (fatty acid transporter), member 4	ENSG00000167114	9	Rare Single Gene Mutation

SLC29A4	solute carrier family 29 member 4	ENSG00000164638	7	Rare Single Gene Mutation
SLC38A10	solute carrier family 38, member 10	ENSG00000157637	17	Rare Single Gene Mutation
SLC45A1	solute carrier family 45 member 1	ENSG00000162426	1	Rare Single Gene Mutation, Syndromic
SLC4A10	solute carrier family 4, sodium bicarbonate transporter-like, member 10	ENSG00000144290	2	Rare Single Gene Mutation, Genetic Association
SLC6A1	Solute carrier family 6 (neurotransmitter transporter), member 1	ENSG00000157103	3	Rare Single Gene Mutation, Syndromic, Genetic Association
SKI	SKIproto-oncogene	ENSG00000157933	1	Rare Single Gene Mutation
SLC6A3	Solute carrier family 6 (neurotransmitter transporter), member 3	ENSG00000142319	5	Rare Single Gene Mutation, Genetic Association, Functional
SLC6A4	solute carrier family 6 (neurotransmitter transporter, serotonin), member 4	ENSG00000108576	17	Rare Single Gene Mutation, Genetic Association
SLC6A8	solute carrier family 6 (neurotransmitter transporter, creatine), member 8	ENSG00000130821	X	Rare Single Gene Mutation, Syndromic
SLC7A3	Solute carrier family 7 (cationic amino acid transporter, y+ system), member 3	ENSG00000165349	X	Rare Single Gene Mutation
SLC7A5	solute carrier family 7 member 5	ENSG00000103257	16	Rare Single Gene Mutation, Functional
SLC7A7	solute carrier family 7 member 7	ENSG00000155465	14	Rare Single Gene Mutation

SLC9A6	solute carrier family 9 (sodium/hydrogen exchanger), member 6	ENSG00000198689	X	Rare Single Gene Mutation, Syndromic, Functional
SLC9A9	solute carrier family 9 (sodium/hydrogen exchanger), member 9	ENSG00000181804	3	Rare Single Gene Mutation, Genetic Association, Functional
SLCO1B3	Solute carrier organic anion transporter family, member 1B3	ENSG00000111700	12	Rare Single Gene Mutation
SLITRK5	SLIT and NTRK like family member 5	ENSG00000165300	13	Rare Single Gene Mutation, Functional
SMAD4	SMAD family member 4	ENSG00000141646	18	Rare Single Gene Mutation
SMARCA2	SWI/SNF related, matrix associated, actin dependent regulator of chromatin, subfamily a, member 2	ENSG00000080503	9	Rare Single Gene Mutation, Syndromic, Genetic Association
SMARCA4	SWI/SNF related, matrix associated, actin dependent regulator of chromatin, subfamily a, member 4	ENSG00000127616	19	Rare Single Gene Mutation, Syndromic
SMARCC2	SWI/SNF related, matrix associated, actin dependent regulator of chromatin, subfamily c, member 2	ENSG00000139613	12	Rare Single Gene Mutation, Syndromic, Functional
SMC1A	structural maintenance of chromosomes 1A	ENSG00000072501	X	Rare Single Gene Mutation, Syndromic
SMC3	structural maintenance of chromosomes 3	ENSG00000108055	10	Rare Single Gene Mutation, Syndromic

SMG6	SMG6, nonsense mediated mRNA decay factor	ENSG00000070366	17	Rare Single Gene Mutation
SMURF1	SMAD specific E3 ubiquitin protein ligase 1	ENSG00000198742	7	Rare Single Gene Mutation
SNAP25	Synaptosomal-associated protein, 25kDa	ENSG00000132639	20	Rare Single Gene Mutation, Genetic Association
SND1	staphylococcal nuclease and tudor domain containing 1	ENSG00000197157	7	Rare Single Gene Mutation, Genetic Association
SNTG2	syntrophin gamma 2	ENSG00000172554	2	Rare Single Gene Mutation
SNX14	Sorting nexin 14	ENSG00000135317	6	Rare Single Gene Mutation, Syndromic
SNX5	sorting nexin 5	ENSG00000089006	20	Rare Single Gene Mutation
SON	SONDNA binding	ENSG00000115904	21	Rare Single Gene Mutation, Syndromic, Functional
SOX5	SRY-box 5	ENSG00000134532	12	Rare Single Gene Mutation, Syndromic, Genetic Association
SPARCL1	SPARC like 1	ENSG00000152583	4	Rare Single Gene Mutation, Functional
SPAST	Spastin	ENSG00000021574	2	Rare Single Gene Mutation
SPEN	spenfamily transcriptional repressor	ENSG00000065526	1	Rare Single Gene Mutation
SPP2	secreted phosphoprotein	ENSG00000072080	2	Rare Single Gene Mutation
SRCAP	Snf2 related CREBBP activator protein	ENSG00000080603	16	Rare Single Gene Mutation
SRGAP3	SLIT-ROBO Rho GTPase activating protein 3	ENSG00000196220	3	Rare Single Gene Mutation
SRSF11	serine and arginine rich splicing factor 11	ENSG00000116754	1	Rare Single Gene Mutation
SSPO	SCO-spondin	ENSG00000197558	7	Rare Single Gene Mutation
SSRP1	structure specific recognition protein 1	ENSG00000149136	11	Rare Single Gene Mutation
ST7	suppression of tumorigenicity 7	ENSG00000004866	7	Rare Single Gene Mutation

ST8SIA2	ST8 alpha-N-acetyl-neuraminide alpha-2,8-sialyltransferase 2	ENSG00000140557	15	Rare Single Gene Mutation, Genetic Association
STAG1	stromal antigen 1	ENSG00000118007	3	Rare Single Gene Mutation, Syndromic
STX1A	Syntaxin 1A (brain)	ENSG00000106089	7	Rare Single Gene Mutation, Genetic Association
STXBP1	Syntaxin binding protein	ENSG00000136854	9	Rare Single Gene Mutation, Syndromic, Functional
SORCS3	sortilin related VPS10 domain containing receptor 3	ENSG00000156395	10	Rare Single Gene Mutation, Syndromic, Genetic Association
STXBP5	Syntaxin binding protein 5 (tomosyn)	ENSG00000164506	6	Rare Single Gene Mutation
STYK1	Serine/threonine/tyrosine kinase 1	ENSG00000060140	12	Rare Single Gene Mutation, Genetic Association
SYAP1	Synapse associated protein 1	ENSG00000169895	X	Rare Single Gene Mutation
SYN1	Synapsin 1	ENSG00000008056	X	Rare Single Gene Mutation, Functional
SYN2	Synapsin II	ENSG00000157152	3	Rare Single Gene Mutation, Genetic Association, Functional
SYNCRIP	synaptotagmin binding cytoplasmic RNA interacting protein	ENSG00000135316	6	Rare Single Gene Mutation
SYNE1	spectrin repeat containing, nuclear	ENSG00000131018	6	Rare Single Gene Mutation, Genetic Association
SYNGAP1	synaptic Ras GTPase activating protein 1	ENSG00000197283	6	Rare Single Gene Mutation, Syndromic
SRPRA	SRP receptor subunit alpha	ENSG00000182934	11	Rare Single Gene Mutation
SYNJ1	synaptojanin 1	ENSG00000159082	21	Rare Single Gene Mutation
SYT1	synaptotagmin 1	ENSG00000067715	12	Rare Single Gene Mutation, Syndromic
TAF1	TATA-box binding protein associated factor 1	ENSG00000147133	X	Rare Single Gene Mutation, Syndromic

TAF1C	TATA-box binding protein associated factor, RNA polymerase I subunit C	ENSG00000103168	16	Rare Single Gene Mutation, Genetic Association
TAF6	TATA-box binding protein associated factor 6	ENSG00000106290	7	Rare Single Gene Mutation
TANC2	etratricopeptide repeat, ankyrin repeat and coiled-coil containing 2	ENSG00000170921	17	Rare Single Gene Mutation, Syndromic
TAOK1	TAO kinase 1	ENSG00000160551	17	Rare Single Gene Mutation, Syndromic
TAOK2	TAO kinase 2	ENSG00000149930	16	Rare Single Gene Mutation, Functional
TBC1D31	TBC1 domain family, member 31	ENSG00000156787	8	Rare Single Gene Mutation
TBC1D5	TBC1 domain family, member 5	ENSG00000131374	3	Rare Single Gene Mutation, Genetic Association
TBL1XR1	transducin beta like 1 X-linked receptor 1	ENSG00000177565	3	Rare Single Gene Mutation, Syndromic
TBR1	T-box, brain, 1	ENSG00000136535	2	Rare Single Gene Mutation, Syndromic, Genetic Association, Functional
SUPT16H	SPT16 homolog, facilitates chromatin remodeling subunit	ENSG00000092201	14	Rare Single Gene Mutation
SYBU	syntabulin	ENSG00000147642	8	Rare Single Gene Mutation
SYCE1	synaptonemal complex central element protein 1	ENSG00000171772	10	Rare Single Gene Mutation
SYP	synaptophysin	ENSG00000102003	X	Rare Single Gene Mutation
TBX22	T-box transcription factor 22	ENSG00000122145	X	Rare Single Gene Mutation
TCF20	Transcription factor 20 (AR1)	ENSG00000100207	22	Rare Single Gene Mutation, Syndromic
TCF4	Transcription factor 4	ENSG00000196628	18	Rare Single Gene Mutation, Syndromic, Genetic Association, Functional

TCF7L2	Transcription factor 7-like 2 (T-cell specific, HMG-box)	ENSG00000148737	10	Rare Single Gene Mutation
TECTA	tectorin alpha	ENSG00000109927	11	Rare Single Gene Mutation
TERF2	Telomeric repeat binding factor 2	ENSG00000132604	16	Rare Single Gene Mutation
TERB2	telomere repeat binding bouquet formation protein 2	ENSG00000167014	15	Rare Single Gene Mutation
TET2	Tet methylcytosine dioxygenase 2	ENSG00000168769	4	Rare Single Gene Mutation
THBS1	Thrombospondin 1	ENSG00000137801	15	Rare Single Gene Mutation, Genetic Association
THRA	thyroid hormone receptor alpha	ENSG00000126351	17	Rare Single Gene Mutation, Functional
TLK2	tousled-like kinase 2	ENSG00000146872	17	Rare Single Gene Mutation, Syndromic
TM4SF19	transmembrane 4 L six family member 19	ENSG00000145107	3	Rare Single Gene Mutation
TM4SF20	Transmembrane 4 L six family member 20	ENSG00000168955	2	Rare Single Gene Mutation
TMEM39B	transmembrane protein 39B	ENSG00000121775	1	Rare Single Gene Mutation
TMLHE	trimethyllysine hydroxylase, epsilon	ENSG00000185973	X	Rare Single Gene Mutation, Genetic Association
TNRC6B	Trinucleotide repeat containing 6B	ENSG00000100354	22	Rare Single Gene Mutation, Syndromic
TOP3B	Topoisomerase (DNA) III beta	ENSG00000100038	22	Rare Single Gene Mutation, Genetic Association, Functional
TRAF7	TNF receptor associated factor 7	ENSG00000131653	16	Rare Single Gene Mutation, Syndromic
TRAPPC6B	trafficking protein particle complex 6B	ENSG00000182400	14	Rare Single Gene Mutation, Syndromic
TRAPPC9	trafficking protein particle complex 9	ENSG00000167632	8	Rare Single Gene Mutation, Syndromic

TRIO	Trio Rho guanine nucleotide exchange factor	ENSG00000038382	5	Rare Single Gene Mutation, Syndromic
TRIP12	Thyroid hormone receptor interactor 12	ENSG00000153827	2	Rare Single Gene Mutation, Syndromic
TRPC6	Transient receptor potential cation channel, subfamily C, member 6	ENSG00000137672	11	Rare Single Gene Mutation
TRPM1	transient receptor potential cation channel subfamily M member 1	ENSG00000134160	15	Rare Single Gene Mutation
TRRAP	transformation/transcription domain associated protein	ENSG00000196367	7	Rare Single Gene Mutation, Syndromic
TSC1	tuberous sclerosis 1	ENSG00000165699	9	Rare Single Gene Mutation, Syndromic
TSC2	tuberous sclerosis 2	ENSG00000103197	16	Rare Single Gene Mutation, Syndromic, Genetic Association
TEK	TEKreceptortyrosine kinase	ENSG00000120156	9	Rare Single Gene Mutation
TET3	tet methylcytosine dioxygenase 3	ENSG00000187605	2	Rare Single Gene Mutation, Syndromic
TFB2M	transcription factor B2, mitochondrial	ENSG00000162851	1	Rare Single Gene Mutation
TM9SF4	transmembrane 9 superfamily member 4	ENSG00000101337	20	Rare Single Gene Mutation
TMEM134	transmembrane protein 134	ENSG00000172663	11	Rare Single Gene Mutation
TRAPPC2L	trafficking protein particle complex 2 like	ENSG00000167515	16	Rare Single Gene Mutation, Syndromic
TRIM23	tripartite motif containing 23	ENSG00000113595	5	Rare Single Gene Mutation
TRIM32	tripartite motif containing 32	ENSG00000119401	9	Rare Single Gene Mutation, Functional

TSHZ3	teashirt zinc finger homeobox 3	ENSG00000121297	19	Rare Single Gene Mutation, Functional
TSPAN17	tetraspanin 17	ENSG00000048140	5	Rare Single Gene Mutation
TSPAN4	tetraspanin 4	ENSG00000214063	11	Rare Single Gene Mutation
TSPAN7	tetraspanin 7	ENSG00000156298	X	Rare Single Gene Mutation
TTI2	TELO2 interacting protein 2	ENSG00000129696	8	Rare Single Gene Mutation
TTN	titin	ENSG00000155657	2	Rare Single Gene Mutation, Syndromic
TNS2	tensin 2	ENSG00000111077	12	Rare Single Gene Mutation
TSPOAP1	TSPO associated protein 1	ENSG00000005379	17	Rare Single Gene Mutation
TUBGCP5	tubulin, gamma complex associated protein 5	ENSG00000275835	15	Rare Single Gene Mutation
UBE3A	ubiquitin protein ligase E3A	ENSG00000114062	15	Rare Single Gene Mutation, Syndromic, Genetic Association
UBE3C	Ubiquitin protein ligase E3C	ENSG00000009335	7	Rare Single Gene Mutation
UBN2	ubinuclein 2	ENSG00000157741	7	Rare Single Gene Mutation
UBR5	ubiquitin protein ligase E3 component n-recognin 5	ENSG00000104517	8	Rare Single Gene Mutation
UIMC1	ubiquitin interaction motif containing 1	ENSG00000087206	5	Rare Single Gene Mutation
UNC13A	unc-13 homolog A	ENSG00000130477	19	Rare Single Gene Mutation, Syndromic
UNC79	unc-79 homolog, NALCN channel complex subunit	ENSG00000133958	14	Rare Single Gene Mutation
UNC80	unc-80 homolog, NALCN activator	ENSG00000144406	2	Rare Single Gene Mutation
UPF2	UPF2, regulator of nonsense mediated mRNA decay	ENSG00000151461	10	Rare Single Gene Mutation, Functional

UPF3B	UPF3B, regulator of nonsense mediated mRNA decay	ENSG00000125351	X	Rare Single Gene Mutation, Syndromic
USH2A	usherin	ENSG00000042781	1	Rare Single Gene Mutation
USP15	ubiquitin specific peptidase 15	ENSG00000135655	12	Rare Single Gene Mutation
USP45	Ubiquitin specific peptidase 45	ENSG00000123552	6	Rare Single Gene Mutation
USP7	Ubiquitin specific peptidase 7 (herpes virus-associated)	ENSG00000187555	16	Rare Single Gene Mutation, Syndromic
USP9X	ubiquitin specific peptidase 9 X-linked	ENSG00000124486	X	Rare Single Gene Mutation, Syndromic, Functional
USP9Y	ubiquitin specific peptidase 9, Y-linked	ENSG00000114374	Y	Rare Single Gene Mutation, Genetic Association
VAMP2	vesicle associated membrane protein 2	ENSG00000220205	17	Rare Single Gene Mutation, Syndromic
VIL1	Villin 1	ENSG00000127831	2	Rare Single Gene Mutation
VPS13B	vacuolar protein sorting 13 homolog B (yeast)	ENSG00000132549	8	Rare Single Gene Mutation, Syndromic
VSIG4	V-set and immunoglobulin domain	ENSG00000155659	X	Rare Single Gene Mutation
WAC	WW domain containing adaptor with coiled-coil	ENSG00000095787	10	Rare Single Gene Mutation, Syndromic
WDFY3	WD repeat and FYVE domain containing 3	ENSG00000163625	4	Rare Single Gene Mutation, Syndromic, Functional
UNC5D	unc-5 netrin receptor D	ENSG00000156687	8	Rare Single Gene Mutation
VEZF1	vascular endothelial zinc finger 1	ENSG00000136451	17	Rare Single Gene Mutation
VWA7	von Willebrand factor A domain containing 7	ENSG00000204396	6	Rare Single Gene Mutation
WDFY4	WDFY family member 4	ENSG00000128815	10	Rare Single Gene Mutation
WDR26	WD repeat domain 26	ENSG00000162923	1	Rare Single Gene Mutation, Syndromic

WNK3	WNK lysine deficient protein kinase 3	ENSG00000196632	X	Rare Single Gene Mutation
WWOX	WW domain containing oxidoreductase	ENSG00000186153	16	Rare Single Gene Mutation, Syndromic
WWP1	WW domain containing E3 ubiquitin protein ligase 1	ENSG00000123124	8	Rare Single Gene Mutation
XPC	xeroderma pigmentosum, complementation group C	ENSG00000154767	3	Rare Single Gene Mutation, Syndromic
XPO1	exportin 1	ENSG00000082898	2	Rare Single Gene Mutation, Genetic Association
YEATS2	YEATS domain containing 2	ENSG00000163872	3	Rare Single Gene Mutation, Genetic Association
YTHDC2	YTH domain containing	ENSG00000047188	5	Rare Single Gene Mutation, Genetic Association
YWHAE	tyrosine 3-monooxygenase/tryptophan 5-monooxygenase activation protein epsilon	ENSG00000108953	17	Rare Single Gene Mutation
UBR1	ubiquitin protein ligase E3 component n-recognin 1	ENSG00000159459	15	Rare Single Gene Mutation
XRCC6	X-ray repair cross complementing 6	ENSG00000196419	22	Rare Single Gene Mutation
YWHAG	tyrosine 3-monooxygenase/tryptophan 5-monooxygenase activation protein gamma	ENSG00000170027	7	Rare Single Gene Mutation, Syndromic
YY1	YY1transcription factor	ENSG00000100811	14	Rare Single Gene Mutation, Syndromic, Functional
ZBTB20	Zinc finger and BTB domain containing 20	ENSG00000181722	3	Rare Single Gene Mutation, Syndromic
ZC3H11A	zinc finger CCCH-type containing 11A	ENSG00000058673	1	Rare Single Gene Mutation
ZC3H4	zinc finger CCCH-type containing 4	ENSG00000130749	19	Rare Single Gene Mutation

ZFYVE26	zinc finger FYVE-type containing 26	ENSG00000072121	14	Rare Single Gene Mutation
ZMYND11	Zinc finger, MYND-type containing 11	ENSG00000015171	10	Rare Single Gene Mutation, Syndromic
ZNF18	zinc finger protein 18	ENSG00000154957	17	Rare Single Gene Mutation
ZNF292	zinc finger protein 292	ENSG00000188994	6	Rare Single Gene Mutation, Syndromic
ZMIZ1	zinc finger MIZ-type containing 1	ENSG00000108175	10	Rare Single Gene Mutation, Syndromic
ZMYND8	zinc finger MYND-type containing 8	ENSG00000101040	20	Rare Single Gene Mutation
ZNF462	Zinc finger protein 462	ENSG00000148143	9	Rare Single Gene Mutation, Syndromic
ZNF517	Zinc finger protein 517	ENSG00000197363	8	Rare Single Gene Mutation
ZNF548	zinc finger protein 548	ENSG00000188785	19	Rare Single Gene Mutation
ZNF559	Zinc finger protein 559	ENSG00000188321	19	Rare Single Gene Mutation
ZNF626	zinc finger protein 626	ENSG00000188171	19	Rare Single Gene Mutation
ZNF713	Zinc finger protein 713	ENSG00000178665	7	Rare Single Gene Mutation
ZNF774	Zinc finger protein 774	ENSG00000196391	15	Rare Single Gene Mutation
ZNF804A	Zinc finger protein 804A	ENSG00000170396	2	Rare Single Gene Mutation, Genetic Association
ZWILCH	zwilchkinetochore protein	ENSG00000174442	15	Rare Single Gene Mutation

Reference: SFARI Gene. [(accessed on 29 October 2020)]; Available online: https://gene.sfari.org/.

Table S2. The known genes with common variants of ASD.

Gene symbol	Gene name	Ensembl ID	Chromosome	Genetics category
ABAT	4-aminobutyrate aminotransferase	ENSG00000183044	16	Rare Single Gene Mutation, Genetic Association
ACE	angiotensin I converting enzyme	ENSG00000159640	17	Rare Single Gene Mutation, Genetic Association
ADA	adenosine deaminase	ENSG00000196839	20	Rare Single Gene Mutation, Genetic Association
AGMO	alkylglycerol monooxygenase	ENSG00000187546	7	Rare Single Gene Mutation, Genetic Association
AHI1	Abelson helper integration site 1	ENSG00000135541	6	Rare Single Gene Mutation, Syndromic, Genetic Association
AMPD1	Adenosine monophosphate deaminase 1	ENSG00000116748	1	Rare Single Gene Mutation, Genetic Association
ANK3	ankyrin 3	ENSG00000151150	10	Rare Single Gene Mutation, Genetic Association
ARNT2	aryl-hydrocarbon receptor nuclear translocator 2	ENSG00000172379	15	Rare Single Gene Mutation, Syndromic, Genetic Association
ASMT	acetylserotonin O-methyltransferase	ENSG00000196433	X,Y	Rare Single Gene Mutation, Genetic Association
ASTN2	astrotactin 2	ENSG00000148219	9	Rare Single Gene Mutation, Genetic Association
ADORA2A	adenosine A2a receptor	ENSG00000128271	22	Genetic Association
ADRB2	adrenergic, beta-2-, receptor, surface	ENSG00000169252	5	Genetic Association
ATP10A	Probable phospholipid-transporting ATPase VA	ENSG00000206190	15	Rare Single Gene Mutation, Genetic Association, Functional
AR	androgen receptor	ENSG00000169083	X	Genetic Association
ATP2B2	ATPase, Ca++ transporting, plasma membrane 2	ENSG00000157087	3	Rare Single Gene Mutation, Genetic Association

AUTS2	autism susceptibility candidate 2	ENSG00000158321	7	Rare Single Gene Mutation, Syndromic, Genetic Association, Functional
AVPR1A	arginine vasopressin receptor 1A	ENSG00000166148	12	Rare Single Gene Mutation, Genetic Association
BST1	bone marrow stromal cell antigen 1	ENSG00000109743	4	Genetic Association, Functional
C4B	complement component 4B	ENSG00000203710	6	Rare Single Gene Mutation, Genetic Association, Functional
CACNA1A	Calcium channel, voltage-dependent, P/Q type, alpha 1A subunit	ENSG00000141837	19	Rare Single Gene Mutation, Genetic Association
CACNA1B	calcium voltage-gated channel subunit alpha1 B	ENSG00000148408	9	Rare Single Gene Mutation, Syndromic, Genetic Association
CACNA1C	calcium channel, voltage-dependent, L type, alpha 1C subunit	ENSG00000151067	12	Rare Single Gene Mutation, Syndromic, Genetic Association, Functional
CACNA1D	calcium channel, voltage-dependent, L type, alpha 1D	ENSG00000157388	3	Rare Single Gene Mutation, Syndromic, Genetic Association, Functional
CACNA1F	calcium channel, voltage-dependent, alpha 1F	ENSG00000102001	X	Rare Single Gene Mutation, Genetic Association
CACNA1G	calcium channel, voltage-dependent, T type, alpha 1G	ENSG00000006283	17	Rare Single Gene Mutation, Syndromic, Genetic Association
AVPR1B	arginine vasopressin receptor 1B	ENSG00000198049	1	Genetic Association, Functional
CACNA1I	Calcium channel, voltage-dependent, T type, alpha 1I subunit	ENSG00000100346	22	Rare Single Gene Mutation, Genetic Association

CACNB2	Calcium channel, voltage-dependent, beta 2 subunit	ENSG00000165995	10	Rare Single Gene Mutation, Genetic Association
CADM2	Cell adhesion molecule 2	ENSG00000175161	3	Rare Single Gene Mutation, Genetic Association
CADPS	calcium dependent secretion activator	ENSG00000163618	3	Rare Single Gene Mutation, Genetic Association
BICDL1	BICD family like cargo adaptor 1	ENSG00000135127	12	Genetic Association
CAMK2A	calcium/calmodulin dependent protein kinase II alpha	ENSG00000070808	5	Rare Single Gene Mutation, Syndromic, Genetic Association, Functional
CAMK4	Calcium/calmodulin-dependent protein kinase IV	ENSG00000152495	5	Genetic Association
CD38	CD38 molecule	ENSG00000004468	4	Rare Single Gene Mutation, Genetic Association, Functional
CD99L2	CD99 molecule like 2	ENSG00000102181	X	Genetic Association
CDH10	cadherin 10, type 2 (T2-cadherin)	ENSG00000040731	5	Rare Single Gene Mutation, Genetic Association
CDH22	cadherin-like 22	ENSG00000149654	20	Genetic Association
CDH9	cadherin 9, type 2 (T1-cadherin)	ENSG00000113100	5	Rare Single Gene Mutation, Genetic Association
CELF6	CUGBP, Elav-like family member 6	ENSG00000140488	15	Rare Single Gene Mutation, Genetic Association
CHRM3	cholinergic receptor muscarinic 3	ENSG00000133019	1	Rare Single Gene Mutation, Genetic Association
CMIP	c-Maf inducing protein	ENSG00000153815	16	Rare Single Gene Mutation, Genetic Association
CNR1	cannabinoid receptor 1 (brain)	ENSG00000118432	6	Rare Single Gene Mutation, Genetic Association
CNTN4	contactin 4	ENSG00000144619	3	Rare Single Gene Mutation, Syndromic, Genetic Association

CNTN5	Contactin 5	ENSG00000149972	11	Rare Single Gene Mutation, Genetic Association
CNTN6	Contactin 6	ENSG00000134115	3	Rare Single Gene Mutation, Genetic Association
CNTNAP2	contactin associated protein-like 2	ENSG00000174469	7	Rare Single Gene Mutation, Syndromic, Genetic Association
CNTNAP5	contactin associated protein-like 5	ENSG00000155052	2	Rare Single Gene Mutation, Genetic Association
CREBBP	CREB binding protein	ENSG00000005339	16	Rare Single Gene Mutation, Syndromic, Genetic Association
CSMD1	CUB and Sushi multiple domains 1	ENSG00000183117	8	Rare Single Gene Mutation, Genetic Association
CTNNA3	catenin (cadherin-associated protein), alpha 3	ENSG00000183230	10	Rare Single Gene Mutation, Genetic Association
CUL3	Cullin 3	ENSG00000036257	2	Rare Single Gene Mutation, Genetic Association
CLIP2	CAP-Gly domain containing linker protein 2	ENSG00000106665	7	Genetic Association
CYFIP1	cytoplasmic FMR1 interacting protein 1	ENSG00000273749	15	Rare Single Gene Mutation, Genetic Association, Functional
DISC1	disrupted in schizophrenia 1	ENSG00000162946	1	Rare Single Gene Mutation, Syndromic, Genetic Association
DDHD2	DDHD domain containing 2	ENSG00000085788	8	Rare Single Gene Mutation, Genetic Association, Functional
DLGAP3	DLG associated protein 3	ENSG00000116544	1	Rare Single Gene Mutation, Genetic Association, Functional
DMD	dystrophin (muscular dystrophy, Duchenne and Becker types)	ENSG00000198947	X	Rare Single Gene Mutation, Syndromic, Genetic Association
DNAH17	dynein axonemal heavy chain 17	ENSG00000187775	17	Rare Single Gene Mutation, Genetic Association
DNER	Delta/notch-like EGF repeat containing	ENSG00000187957	2	Rare Single Gene Mutation, Genetic Association

DNMT3A	DNA (cytosine-5-)-methyltransferase 3 alpha	ENSG00000119772	2	Rare Single Gene Mutation, Syndromic, Genetic Association
DOCK4	Dedicator of cytokinesis 4	ENSG00000128512	7	Rare Single Gene Mutation, Genetic Association, Functional
DPP10	Dipeptidyl-peptidase 10	ENSG00000175497	2	Rare Single Gene Mutation, Genetic Association
DPP4	Dipeptidyl-peptidase 4	ENSG00000197635	2	Rare Single Gene Mutation, Genetic Association
DPP6	dipeptidyl-peptidase 6	ENSG00000130226	7	Rare Single Gene Mutation, Genetic Association, Functional
DPYD	dihydropyrimidine dehydrogenase	ENSG00000188641	1	Rare Single Gene Mutation, Syndromic, Genetic Association
DPYSL2	dihydropyrimidinase like 2	ENSG00000092964	8	Rare Single Gene Mutation, Genetic Association, Functional
DRD2	Dopamine receptor D2	ENSG00000149295	11	Rare Single Gene Mutation, Genetic Association
DRD3	dopamine receptor D3	ENSG00000151577	3	Rare Single Gene Mutation, Genetic Association
DSCAM	Down syndrome cell adhesion molecule	ENSG00000171587	21	Rare Single Gene Mutation, Genetic Association
DUSP15	dual specificity phosphatase 15	ENSG00000149599	20	Rare Single Gene Mutation, Genetic Association
DDC	dopa decarboxylase	ENSG00000132437	7	Genetic Association
DLX2	distal-less homeobox 2	ENSG00000115844	2	Genetic Association
DRD1	Dopamine receptor D1	ENSG00000184845	5	Genetic Association, Functional
DCC	DCCnetrin 1 receptor	ENSG00000187323	18	Genetic Association
EIF4E	eukaryotic translation initiation factor 4E	ENSG00000151247	4	Rare Single Gene Mutation, Genetic Association
ELAVL2	ELAV like neuron-specific RNA binding protein 2	ENSG00000107105	9	Rare Single Gene Mutation, Genetic Association, Functional

ELP4	Elongator acetyltransferase complex subunit 4	ENSG00000109911	11	Rare Single Gene Mutation, Genetic Association
EN2	engrailed homolog 2	ENSG00000164778	7	Rare Single Gene Mutation, Genetic Association, Functional
ESR2	estrogen receptor 2 (ER beta)	ENSG00000140009	14	Rare Single Gene Mutation, Syndromic, Genetic Association
ESRRB	estrogen-related receptor beta	ENSG00000119715	14	Rare Single Gene Mutation, Genetic Association
EXT1	Exostosin 1	ENSG00000182197	8	Rare Single Gene Mutation, Genetic Association
FAN1	FANCD2/FANCI-associated nuclease 1	ENSG00000198690	15	Rare Single Gene Mutation, Genetic Association
FBXO40	F-box protein 40	ENSG00000163833	3	Rare Single Gene Mutation, Genetic Association
FEZF2	FEZ family zinc finger 2	ENSG00000153266	3	Rare Single Gene Mutation, Genetic Association
FGA	Fibrinogen alpha	ENSG00000171560	4	Rare Single Gene Mutation, Genetic Association
FHIT	fragile histidine triad gene	ENSG00000189283	3	Rare Single Gene Mutation, Genetic Association
FMR1	fragile X mental retardation 1	ENSG00000102081	X	Rare Single Gene Mutation, Syndromic, Genetic Association, Functional
FOXG1	Forkhead box G1	ENSG00000176165	14	Rare Single Gene Mutation, Syndromic, Genetic Association
FOXP1	forkhead box P1	ENSG00000114861	3	Rare Single Gene Mutation, Syndromic, Genetic Association, Functional
ELOVL2	ELOVL fatty acid elongase 2	ENSG00000197977	6	Genetic Association
ERG	ERG, ETS transcription factor	ENSG00000157554	21	Genetic Association
FBXO33	F-box protein 33	ENSG00000165355	14	Genetic Association
FOXP2	forkhead box P2	ENSG00000128573	7	Rare Single Gene Mutation, Genetic Association
FRK	fyn-related kinase	ENSG00000111816	6	Rare Single Gene Mutation, Genetic Association

GABBR2	gamma-aminobutyric acid type B receptor subunit 2	ENSG00000136928	9	Rare Single Gene Mutation, Syndromic, Genetic Association, Functional
GABRA4	gamma-aminobutyric acid (GABA) A receptor, alpha 4	ENSG00000109158	4	Rare Single Gene Mutation, Genetic Association, Functional
GABRB3	gamma-aminobutyric acid (GABA) A receptor, beta 3	ENSG00000166206	15	Rare Single Gene Mutation, Genetic Association
GABRG3	gamma-aminobutyric acid type A receptor gamma3 subunit	ENSG00000182256	15	Genetic Association
GALNT14	polypeptide N-acetylgalactosaminyltransferase 14	ENSG00000158089	2	Rare Single Gene Mutation, Genetic Association
GAS2	Growth arrest-specific 2	ENSG00000148935	11	Genetic Association
GDA	guanine deaminase	ENSG00000119125	9	Rare Single Gene Mutation, Genetic Association
GLIS1	GLIS family zinc finger 1	ENSG00000174332	1	Rare Single Gene Mutation, Genetic Association
GLO1	glyoxalase I	ENSG00000124767	6	Rare Single Gene Mutation, Genetic Association
GNB1L	guanine nucleotide binding protein (G protein), beta polypeptide 1-like	ENSG00000185838	22	Rare Single Gene Mutation, Genetic Association
GPC6	glypican 6	ENSG00000183098	13	Rare Single Gene Mutation, Genetic Association
GPD2	glycerol-3-phosphate dehydrogenase 2	ENSG00000115159	2	Rare Single Gene Mutation, Genetic Association
GPR85	G protein-coupled receptor 85	ENSG00000164604	7	Rare Single Gene Mutation, Genetic Association
GPX1	glutathione peroxidase 1	ENSG00000233276	3	Genetic Association

GRID1	Glutamate receptor, ionotropic, delta 1	ENSG00000182771	10	Rare Single Gene Mutation, Genetic Association
GRID2	glutamate receptor, ionotropic, delta 2	ENSG00000152208	4	Rare Single Gene Mutation, Syndromic, Genetic Association
GRIK2	glutamate ionotropic receptor kainate type subunit 2	ENSG00000164418	6	Rare Single Gene Mutation, Genetic Association
FGFR1	fibroblast growth factor receptor 1	ENSG00000077782	8	Genetic Association
GALNT10	polypeptide N-acetylgalactosaminyltr ansferase 10	ENSG00000164574	5	Genetic Association
GRIK3	glutamate ionotropic receptor kainate type subunit 3	ENSG00000163873	1	Rare Single Gene Mutation, Genetic Association
GRIN2A	glutamate receptor, ionotropic, N-methyl D-aspartate 2A	ENSG00000183454	16	Rare Single Gene Mutation, Syndromic, Genetic Association
GRIN2B	glutamate receptor, inotropic, N-methyl D-apartate 2B	ENSG00000273079	12	Rare Single Gene Mutation, Syndromic, Genetic Association
GRM5	glutamate metabotropic receptor 5	ENSG00000168959	11	Rare Single Gene Mutation, Genetic Association, Functional
GRM7	Glutamate receptor, metabotropic 7	ENSG00000196277	3	Rare Single Gene Mutation, Genetic Association
GTF2I	general transcription factor IIi	ENSG00000263001	7	Rare Single Gene Mutation, Genetic Association
GUCY1A2	guanylate cyclase 1 soluble subunit alpha 2	ENSG00000152402	11	Rare Single Gene Mutation, Genetic Association

HCN1	Hyperpolarization activated cyclic nucleotide-gated potassium channel 1	ENSG00000164588	5	Rare Single Gene Mutation, Genetic Association
HDAC4	histone deacetylase 4	ENSG00000068024	2	Rare Single Gene Mutation, Syndromic, Genetic Association
HIVEP3	human immunodeficiency virus type I enhancer binding protein 3	ENSG00000127124	1	Rare Single Gene Mutation, Genetic Association
HLA-DPB1	major histocompatibility complex, class II, DP beta 1	ENSG00000223865	6	Rare Single Gene Mutation, Genetic Association
HOXA1	homeobox A1	ENSG00000105991	7	Rare Single Gene Mutation, Syndromic, Genetic Association
HRAS	v-Ha-ras Harvey rat sarcoma viral oncogene homolog	ENSG00000174775	11	Rare Single Gene Mutation, Syndromic, Genetic Association
GSTM1	glutathione S-transferase M1	ENSG00000134184	1	Genetic Association
HLA-A	major histocompatibility complex, class I, A	ENSG00000206503	6	Genetic Association
HLA-B	Major histocompatibility complex, class I, B	ENSG00000234745	6	Genetic Association
HLA-DRB1	major histocompatibility complex, class II, DR beta 1	ENSG00000196126	6	Genetic Association

HLA-G	major histocompatibility complex, class I, G	ENSG00000204632	6	Genetic Association
HMGN1	high mobility group nucleosome binding domain 1	ENSG00000205581	21	Genetic Association
HS3ST5	heparan sulfate (glucosamine) 3-O-sulfotransferase 5	ENSG00000249853	6	Genetic Association
HTR1B	5-hydroxytryptamine (serotonin) receptor 1B	ENSG00000135312	6	Rare Single Gene Mutation, Genetic Association
HTR3A	5-hydroxytryptamine (serotonin) receptor 3A	ENSG00000166736	11	Rare Single Gene Mutation, Genetic Association, Functional
HTR3C	5-hydroxytryptamine (serotonin) receptor 3, family member C	ENSG00000178084	3	Rare Single Gene Mutation, Genetic Association
HYDIN	HYDIN, axonemal central pair apparatus protein	ENSG00000157423	16	Rare Single Gene Mutation, Genetic Association
IL1RAPL2	interleukin 1 receptor accessory protein-like 2	ENSG00000189108	X	Rare Single Gene Mutation, Genetic Association
IMMP2L	IMP2 inner mitochondrial membrane peptidase-like (S. cerevisiae)	ENSG00000184903	7	Rare Single Gene Mutation, Genetic Association, Functional
INPP1	inositol polyphosphate-1-phosphatase	ENSG00000151689	2	Rare Single Gene Mutation, Genetic Association

ITGB3	integrin, beta 3 (platelet glycoprotein IIIa, antigen CD61)	ENSG00000259207	17	Rare Single Gene Mutation, Genetic Association
ITPR1	inositol 1,4,5-trisphosphate receptor type 1	ENSG00000150995	3	Rare Single Gene Mutation, Genetic Association
JARID2	jumonji and AT-rich interaction domain containing 2	ENSG00000008083	6	Rare Single Gene Mutation, Syndromic, Genetic Association
KCND2	potassium voltage-gated channel subfamily D member 2	ENSG00000184408	7	Rare Single Gene Mutation, Genetic Association
KCND3	potassium voltage-gated channel subfamily D member 3	ENSG00000171385	1	Rare Single Gene Mutation, Syndromic, Genetic Association
KCNJ10	potassium voltage-gated channel subfamily J member 10	ENSG00000177807	1	Rare Single Gene Mutation, Syndromic, Genetic Association
KCNQ2	potassium voltage-gated channel subfamily Q member 2	ENSG00000075043	20	Rare Single Gene Mutation, Syndromic, Genetic Association
KCNQ3	potassium voltage-gated channel subfamily Q member 3	ENSG00000184156	8	Rare Single Gene Mutation, Genetic Association
KMT2E	Lysine (K)-specific methyltransferase 2E	ENSG00000005483	7	Rare Single Gene Mutation, Syndromic, Genetic Association

KRR1	KRR1, small subunit (SSU) processome component, homolog (yeast)	ENSG00000111615	12	Rare Single Gene Mutation, Genetic Association
LAMA1	Laminin, alpha 1	ENSG00000101680	18	Rare Single Gene Mutation, Genetic Association
LAMB1	laminin, beta 1	ENSG00000091136	7	Rare Single Gene Mutation, Genetic Association
LRFN2	leucine rich repeat and fibronectin type III domain containing	ENSG00000156564	6	Rare Single Gene Mutation, Genetic Association, Functional
LRFN5	leucine rich repeat and fibronectin type III domain containing	ENSG00000165379	14	Rare Single Gene Mutation, Genetic Association
LRP2	LDL receptor related protein 2	ENSG00000081479	2	Rare Single Gene Mutation, Syndromic, Genetic Association
LRRC1	leucine rich repeat containing 1	ENSG00000137269	6	Rare Single Gene Mutation, Genetic Association
MACROD2	MACRO domain containing 2	ENSG00000172264	20	Rare Single Gene Mutation, Genetic Association
MAOA	monoamine oxidase A	ENSG00000189221	X	Rare Single Gene Mutation, Syndromic, Genetic Association, Functional
MAOB	monoamine oxidase B	ENSG00000069535	X	Rare Single Gene Mutation, Genetic Association, Functional
MARK1	microtubule affinity regulating kinase 1	ENSG00000116141	1	Rare Single Gene Mutation, Genetic Association
MDGA2	MAM domain containing glycosylphosphatidylinositol anchor 2	ENSG00000139915	14	Rare Single Gene Mutation, Genetic Association
LMX1B	LIM homeobox transcription factor 1 beta	ENSG00000136944	9	Genetic Association

LZTS2	leucine zipper, putative tumor suppressor 2	ENSG00000107816	10	Genetic Association
MAPT-AS1	MAPT antisense RNA 1	ENSG00000264589	17	Genetic Association
MEF2C	myocyte enhancer factor 2C	ENSG00000081189	5	Rare Single Gene Mutation, Syndromic, Genetic Association, Functional
MEGF10	multiple EGF like domains 10	ENSG00000145794	5	Rare Single Gene Mutation, Genetic Association
MET	met proto-oncogene (hepatocyte growth factor receptor)	ENSG00000105976	7	Rare Single Gene Mutation, Genetic Association, Functional
MNT	MAX network transcriptional repressor	ENSG00000070444	17	Genetic Association
MIR137	microRNA 137	ENSG00000284202	1	Rare Single Gene Mutation, Syndromic, Genetic Association, Functional
MRTFB	myocardin related transcription factor B	ENSG00000186260	16	Rare Single Gene Mutation, Genetic Association
MSNP1AS	Moesinpseudogene 1, antisense	ENSG00000251593	5	Genetic Association, Functional
MTF1	metal-regulatory transcription factor 1	ENSG00000188786	1	Syndromic, Genetic Association
MTHFR	methylenetetrahydrofolate reductase (NAD(P)H)	ENSG00000177000	1	Rare Single Gene Mutation, Syndromic, Genetic Association
MYO16	myosin XVI	ENSG00000041515	13	Rare Single Gene Mutation, Genetic Association
MYO5A	myosin VA	ENSG00000197535	15	Rare Single Gene Mutation, Genetic Association
MYT1L	Myelin transcription factor 1-like	ENSG00000186487	2	Rare Single Gene Mutation, Syndromic, Genetic Association
NAALADL2	N-acetylated alpha-linked acidic dipeptidase-like 2	ENSG00000177694	3	Rare Single Gene Mutation, Genetic Association

NEGR1	neuronal growth regulator 1	ENSG00000172260	1	Rare Single Gene Mutation, Genetic Association, Functional
NF1	neurofibromin 1 (neurofibromatosis, von Recklinghausen disease, Watson disease)	ENSG00000196712	17	Rare Single Gene Mutation, Syndromic, Genetic Association
NLGN1	neuroligin 1	ENSG00000169760	3	Rare Single Gene Mutation, Genetic Association
NLGN4X	neuroligin 4, X-linked	ENSG00000146938	X	Rare Single Gene Mutation, Genetic Association
NLGN4Y	neuroligin 4, Y-linked	ENSG00000165246	Y	Rare Single Gene Mutation, Genetic Association, Functional
NRCAM	neuronal cell adhesion molecule	ENSG00000091129	7	Rare Single Gene Mutation, Genetic Association
NRP2	neuropilin 2	ENSG00000118257	2	Rare Single Gene Mutation, Genetic Association
NRXN1	neurexin 1	ENSG00000179915	2	Rare Single Gene Mutation, Syndromic, Genetic Association, Functional
NDUFA5	NADH dehydrogenase (ubiquinone) 1 alpha	ENSG00000128609	7	Genetic Association
NPAS2	neuronal PAS domain protein 2	ENSG00000170485	2	Genetic Association
NRXN2	neurexin 2	ENSG00000110076	11	Rare Single Gene Mutation, Genetic Association
NRXN3	neurexin 3	ENSG00000021645	14	Rare Single Gene Mutation, Genetic Association
NTNG1	netrin G1	ENSG00000162631	1	Rare Single Gene Mutation, Syndromic, Genetic Association
NTRK3	neurotrophic tyrosine kinase, receptor, type 3	ENSG00000140538	15	Rare Single Gene Mutation, Syndromic, Genetic Association
OR2M4	Olfactory receptor, family 2, subfamily M, member 4	ENSG00000171180	1	Genetic Association
OTX1	orthodenticle homeobox 1	ENSG00000115507	2	Genetic Association

OXT	oxytocin/neurophysin I prepropeptide	ENSG00000101405	20	Rare Single Gene Mutation, Genetic Association
OXTR	oxytocin receptor	ENSG00000180914	3	Rare Single Gene Mutation, Genetic Association, Functional
PARD3B	Par-3 partitioning defective 3 homolog B (C. elegans)	ENSG00000116117	2	Rare Single Gene Mutation, Genetic Association
PATJ	PATJ, crumbs cell polarity complex component	ENSG00000132849	1	Rare Single Gene Mutation, Genetic Association
PCDH15	protocadherin related 15	ENSG00000150275	10	Rare Single Gene Mutation, Syndromic, Genetic Association
PCDH9	protocadherin 9	ENSG00000184226	13	Rare Single Gene Mutation, Genetic Association
PCDHA1	Protocadherin alpha 1	ENSG00000204970	5	Rare Single Gene Mutation, Genetic Association
PCDHA10	Protocadherin alpha 10	ENSG00000250120	5	Rare Single Gene Mutation, Genetic Association
PCDHA11	Protocadherin alpha 11	ENSG00000249158	5	Rare Single Gene Mutation, Genetic Association
PCDHA12	Protocadherin alpha 12	ENSG00000251664	5	Rare Single Gene Mutation, Genetic Association
PCDHA13	Protocadherin alpha 13	ENSG00000239389	5	Rare Single Gene Mutation, Genetic Association
PCDHA2	Protocadherin alpha 2	ENSG00000204969	5	Rare Single Gene Mutation, Genetic Association
PCDHA3	Protocadherin alpha 3	ENSG00000255408	5	Rare Single Gene Mutation, Genetic Association
PCDHA4	Protocadherin alpha 4	ENSG00000204967	5	Rare Single Gene Mutation, Genetic Association
PCDHA5	Protocadherin alpha 5	ENSG00000204965	5	Rare Single Gene Mutation, Genetic Association
PCDHA6	Protocadherin alpha 6	ENSG00000081842	5	Rare Single Gene Mutation, Genetic Association
PCDHA7	Protocadherin alpha 7	ENSG00000204963	5	Rare Single Gene Mutation, Genetic Association
PCDHA8	Protocadherin alpha 8	ENSG00000204962	5	Rare Single Gene Mutation, Genetic Association
PCDHA9	Protocadherin alpha 9	ENSG00000204961	5	Rare Single Gene Mutation, Genetic Association
PCDHAC1	Protocadherin alpha subfamily C, 1	ENSG00000248383	5	Rare Single Gene Mutation, Genetic Association
PDE1C	phosphodiesterase 1C	ENSG00000154678	7	Rare Single Gene Mutation, Genetic Association

PER1	period homolog 1 (Drosophila)	ENSG00000179094	17	Rare Single Gene Mutation, Genetic Association
PEX7	peroxisomal biogenesis factor 7	ENSG00000112357	6	Rare Single Gene Mutation, Genetic Association
PITX1	paired-like homeodomain 1	ENSG00000069011	5	Rare Single Gene Mutation, Genetic Association
PLAUR	Plasminogen activator, urokinase	ENSG00000011422	19	Rare Single Gene Mutation, Genetic Association
PREX1	Phosphatidylinositol-3,4,5-trisphosphate-dependent Rac exchange factor 1	ENSG00000124126	20	Rare Single Gene Mutation, Genetic Association
PRKCB	protein kinase C beta	ENSG00000166501	16	Rare Single Gene Mutation, Genetic Association
PRKN	parkin RBR E3 ubiquitin protein ligase	ENSG00000185345	6	Rare Single Gene Mutation, Genetic Association
PRODH	Proline dehydrogenase	ENSG00000100033	22	Syndromic, Genetic Association
PSD3	pleckstrin and Sec7 domain containing 3	ENSG00000156011	8	Rare Single Gene Mutation, Genetic Association
PTBP2	polypyrimidine tract binding protein 2	ENSG00000117569	1	Rare Single Gene Mutation, Genetic Association
PTCHD1	patched domain containing 1	ENSG00000165186	X	Rare Single Gene Mutation, Genetic Association
PCDHAC2	Protocadherin alpha subfamily C, 2	ENSG00000243232	5	Genetic Association
PHB	prohibitin	ENSG00000167085	17	Genetic Association
PIK3CG	phosphoinositide-3-kinase, catalytic, gamma polypeptide	ENSG00000105851	7	Genetic Association
PON1	paraoxonase 1	ENSG00000005421	7	Genetic Association

PPP1R1B	Protein phosphatase 1, regulatory (inhibitor) subunit 1B	ENSG00000131771	17	Genetic Association
PTGS2	prostaglandin-endoperoxide synthase 2	ENSG00000073756	1	Rare Single Gene Mutation, Genetic Association, Functional
PTPRB	protein tyrosine phosphatase, receptor type B	ENSG00000127329	12	Rare Single Gene Mutation, Genetic Association
PTPRC	protein tyrosine phosphatase, receptor type, C	ENSG00000081237	1	Rare Single Gene Mutation, Genetic Association
RASSF5	Ras association domain family member 5	ENSG00000266094	1	Rare Single Gene Mutation, Genetic Association
RBFOX1	RNA binding protein, fox-1 homolog (C. elegans) 1	ENSG00000078328	16	Rare Single Gene Mutation, Genetic Association
RELN	Reelin	ENSG00000189056	7	Rare Single Gene Mutation, Syndromic, Genetic Association, Functional
RERE	Arginine-glutamic acid dipeptide (RE) repeats	ENSG00000142599	1	Rare Single Gene Mutation, Syndromic, Genetic Association
RIMS1	Regulating synaptic membrane exocytosis 1	ENSG00000079841	6	Rare Single Gene Mutation, Genetic Association
RNF135	Ring finger protein 135	ENSG00000181481	17	Rare Single Gene Mutation, Syndromic, Genetic Association
ROBO2	roundabout guidance receptor 2	ENSG00000185008	3	Rare Single Gene Mutation, Genetic Association, Functional
RORA	RAR-related orphan receptor A	ENSG00000069667	15	Rare Single Gene Mutation, Syndromic, Genetic Association, Functional

SATB2	SATB homeobox 2	ENSG00000119042	2	Rare Single Gene Mutation, Syndromic, Genetic Association
RIMS2	regulating synaptic membrane exocytosis 2	ENSG00000176406	8	Rare Single Gene Mutation, Syndromic, Genetic Association
RSRC1	arginine and serine rich coiled-coil 1	ENSG00000174891	3	Syndromic, Genetic Association
SCN1A	sodium channel, voltage-gated, type I, alpha subunit	ENSG00000144285	2	Rare Single Gene Mutation, Syndromic, Genetic Association
RHOXF1	Rhox homeobox family, member 1	ENSG00000101883	X	Genetic Association
RIT2	Ras-like without CAAX 2	ENSG00000152214	18	Genetic Association
RPS10P2-AS1	ribosomal protein S10 pseudogene 2 anti-sense 1	ENSG00000124614	20	Genetic Association, Functional
SDC2	syndecan 2 (heparan sulfate proteoglycan 1, cell surface-associated, fibroglycan)	ENSG00000169439	8	Rare Single Gene Mutation, Genetic Association
SEMA5A	sema domain, seven thrombospondin repeats (type 1 and type 1-like), transmembrane domain (TM) and short cytoplasmic domain, (semaphorin) 5A	ENSG00000112902	5	Rare Single Gene Mutation, Genetic Association, Functional
SERPINE1	serpin family E member 1	ENSG00000106366	7	Genetic Association

SEZ6L2	SEZ6L2 seizure related 6 homolog (mouse)-like 2	ENSG00000174938	16	Rare Single Gene Mutation, Genetic Association
SHANK1	SH3 and multiple ankyrin repeat domains 1	ENSG00000161681	19	Rare Single Gene Mutation, Genetic Association
SHANK2	SH3 and multiple ankyrin repeat domains 2	ENSG00000162105	11	Rare Single Gene Mutation, Syndromic, Genetic Association, Functional
SHANK3	SH3 and multiple ankyrin repeat domains 3	ENSG00000251322	22	Rare Single Gene Mutation, Syndromic, Genetic Association, Functional
SLC1A1	solute carrier family 1 (neuronal/epithelial high affinity glutamate transporter, system Xag), member	ENSG00000106688	9	Rare Single Gene Mutation, Genetic Association
SLC1A2	Solute carrier family 1 (glial high affinity glutamate transporter), member	ENSG00000110436	11	Rare Single Gene Mutation, Genetic Association, Functional
SLC22A15	Solute carrier family 22, member 15	ENSG00000163393	1	Genetic Association
SLC25A12	solute carrier family 25 (mitochondrial carrier, Aralar), member 12	ENSG00000115840	2	Rare Single Gene Mutation, Genetic Association, Functional
SLC25A27	solute carrier family 25 member 27	ENSG00000153291	6	Genetic Association
SLC35B1	solute carrier family 35 member B1	ENSG00000121073	17	Genetic Association

SLC4A10	solute carrier family 4, sodium bicarbonate transporter-like, member 10	ENSG00000144290	2	Rare Single Gene Mutation, Genetic Association
SLC6A1	Solute carrier family 6 (neurotransmitter transporter), member	ENSG00000157103	3	Rare Single Gene Mutation, Syndromic, Genetic Association
SLC6A3	Solute carrier family 6 (neurotransmitter transporter), member	ENSG00000142319	5	Rare Single Gene Mutation, Genetic Association, Functional
SLC6A4	solute carrier family 6 (neurotransmitter transporter, serotonin), member 4	ENSG00000108576	17	Rare Single Gene Mutation, Genetic Association
SLC9A9	solute carrier family 9 (sodium/hydrogen exchanger), member 9	ENSG00000181804	3	Rare Single Gene Mutation, Genetic Association, Functional
SMARCA2	SWI/SNF related, matrix associated, actin dependent regulator of chromatin, subfamily a, member 2	ENSG00000080503	9	Rare Single Gene Mutation, Syndromic, Genetic Association
SNAP25	Synaptosomal-associated protein, 25kDa	ENSG00000132639	20	Rare Single Gene Mutation, Genetic Association
SND1	staphylococcal nuclease and tudor domain containing 1	ENSG00000197157	7	Rare Single Gene Mutation, Genetic Association
SOX5	SRY-box 5	ENSG00000134532	12	Rare Single Gene Mutation, Syndromic, Genetic Association

ST8SIA2	ST8 alpha-N-acetyl-neuraminide alpha-2,8-sialyltransferase 2	ENSG00000140557	15	Rare Single Gene Mutation, Genetic Association
STX1A	Syntaxin 1A (brain)	ENSG00000106089	7	Rare Single Gene Mutation, Genetic Association
SORCS3	sortilin related VPS10 domain containing receptor 3	ENSG00000156395	10	Rare Single Gene Mutation, Syndromic, Genetic Association
STYK1	Serine/threonine/tyrosine kinase 1	ENSG00000060140	12	Rare Single Gene Mutation, Genetic Association
SYN2	Synapsin II	ENSG00000157152	3	Rare Single Gene Mutation, Genetic Association, Functional
SYNE1	spectrin repeat containing, nuclear envelope 1	ENSG00000131018	6	Rare Single Gene Mutation, Genetic Association
TAF1C	TATA-box binding protein associated factor, RNA polymerase I subunit C	ENSG00000103168	16	Rare Single Gene Mutation, Genetic Association
TBC1D5	TBC1 domain family, member 5	ENSG00000131374	3	Rare Single Gene Mutation, Genetic Association
TBR1	T-box, brain, 1	ENSG00000136535	2	Rare Single Gene Mutation, Syndromic, Genetic Association, Functional
SOD1	superoxide dismutase 1	ENSG00000142168	21	Genetic Association, Functional
STK39	serine threonine kinase 39 (STE20/SPS1 homolog, yeast)	ENSG00000198648	2	Genetic Association
SYT17	synaptotagmin XVII	ENSG00000103528	16	Genetic Association
TBL1X	transducin (beta)-like 1X-linked	ENSG00000101849	X	Genetic Association

TCF4	Transcription factor 4	ENSG00000196628	18	Rare Single Gene Mutation, Syndromic, Genetic Association, Functional
THBS1	Thrombospondin 1	ENSG00000137801	15	Rare Single Gene Mutation, Genetic Association
TMLHE	trimethyllysine hydroxylase, epsilon	ENSG00000185973	X	Rare Single Gene Mutation, Genetic Association
TOP3B	Topoisomerase (DNA) III beta	ENSG00000100038	22	Rare Single Gene Mutation, Genetic Association, Functional
TSC2	tuberous sclerosis 2	ENSG00000103197	16	Rare Single Gene Mutation, Syndromic, Genetic Association
UBE3A	ubiquitin protein ligase E3A	ENSG00000114062	15	Rare Single Gene Mutation, Syndromic, Genetic Association
USP9Y	ubiquitin specific peptidase 9, Y-linked	ENSG00000114374	Y	Rare Single Gene Mutation, Genetic Association
TDO2	tryptophan 2,3-dioxygenase	ENSG00000151790	4	Genetic Association
TPO	Thyroid peroxidase	ENSG00000115705	2	Genetic Association
TRIM33	Tripartite motif containing 33	ENSG00000197323	1	Genetic Association
TTC25	tetratricopeptide repeat domain 25	ENSG00000204815	17	Genetic Association
UBE2H	ubiquitin-conjugating enzyme E2H (UBC8 homolog, yeast)	ENSG00000186591	7	Genetic Association
VASH1	vasohibin 1	ENSG00000071246	14	Genetic Association
VDR	vitamin D receptor	ENSG00000111424	12	Genetic Association, Functional
WNT1	Wingless-type MMTV integration site family, member 1	ENSG00000125084	12	Genetic Association
XPO1	exportin 1	ENSG00000082898	2	Rare Single Gene Mutation, Genetic Association
YEATS2	YEATS domain containing 2	ENSG00000163872	3	Rare Single Gene Mutation, Genetic Association
YTHDC2	YTH domain containing 2	ENSG00000047188	5	Rare Single Gene Mutation, Genetic Association

ZBTB16	Zinc finger and BTB domain containing 16	ENSG00000109906	11	Genetic Association
ZNF385B	Zinc finger protein 385B	ENSG00000144331	2	Genetic Association
ZNF804A	Zinc finger protein 804A	ENSG00000170396	2	Rare Single Gene Mutation, Genetic Association
ZNF827	Zinc finger protein 827	ENSG00000151612	4	Genetic Association
ZSWIM6	zinc finger SWIM-type containing 6	ENSG00000130449	5	Syndromic, Genetic Association
AFF2	AF4/FMR2 family, member 2	ENSG00000155966	X	Rare Single Gene Mutation, Syndromic
BCL11A	B-cell CLL/lymphoma 11A (zinc finger protein)	ENSG00000119866	2	Rare Single Gene Mutation, Syndromic
CNTNAP4	Contactin associated protein-like 4	ENSG00000152910	16	Rare Single Gene Mutation, Functional
CX3CR1	Chemokine (C-X3-C motif) receptor 1	ENSG00000168329	3	Rare Single Gene Mutation, Functional
CYP11B1	cytochrome P450, family 11, subfamily B, polypeptide 1	ENSG00000160882	8	Syndromic
DMPK	dystrophia myotonica-protein kinase	ENSG00000104936	19	Rare Single Gene Mutation, Syndromic
HSD11B1	hydroxysteroid (11-beta) dehydrogenase 1	ENSG00000117594	1	Syndromic
MBD5	Methyl-CpG binding domain protein 5	ENSG00000204406	2	Rare Single Gene Mutation, Syndromic, Functional
NTRK1	neurotrophic tyrosine kinase, receptor, type 1	ENSG00000198400	1	Rare Single Gene Mutation, Syndromic
RAI1	retinoic acid induced 1	ENSG00000108557	17	Rare Single Gene Mutation, Syndromic

Reference: SFARI Gene. [(accessed on 29 October 2020)]; Available online: https://gene.sfari.org/.

Table S3. Known ASD recessive inherited risk genes.

Gene symbol	Location	#OMIM	Inheritance	#RefSeq	Ensembl ID
ABAT	16p13.2	137150	AR	NM_020686.5	ENSG00000183044
ACE	17q23.3	106180	AR	NM_000789.3	ENSG00000159640
ACTL6B	7q22.1	612458	AD/AR	NM_016188.4	ENSG00000077080
ACY1	3p21.2	104620	AR	NM_000666.2	ENSG00000243989
ADA	20q13.12	608958	AR	NM_000022.3	ENSG00000196839
ADCY3	2p23.3	600291	AR	NM_004036.4	ENSG00000138031
ADK	10q22.2	102750	AR	NM_001123.3	ENSG00000156110
ADSL	22q13.1	608222	AR	NM_000026.3	ENSG00000239900
AFF2	Xq28	300806	XLR	NM_002025.3	ENSG00000155966
AHI1	6q23.3	608894	AR	NM_017651.4	ENSG00000135541
ALDH1A3	15q26.3	600463	AR	NM_000693.3	ENSG00000184254
ALDH5A1	6p22.2-p22.3	610045	AR	NM_001080.3	ENSG00000112294
AMPD1	1p13.2	102770	AR	NM_000036.2	ENSG00000116748
AMT	3p21.31	238310	AR	NM_000481.3	ENSG00000145020
ANK3	10q21	600465	AR	NM_020987.4	ENSG00000151150
AP1S2	Xp22.2	300629	XLR	NM_003916.4	ENSG00000182287
ARHGEF9	Xq11.1	300429	XLR	NM_015185.2	ENSG00000131089
ARNT2	15q25.1	606036	AR	NM_014862.3	ENSG00000172379
ARX	Xp21	300382	XLR	NM_139058.2	ENSG00000004848
ASPM	1q31.3	605481	AR	NM_018136.4	ENSG00000066279
ALG6	1p31.3	604566	AR	NM_013339.3	ENSG00000088035
AR	Xq12	313700	XLR	NM_000044.4	ENSG00000169083
ATP2B2	3p25.3	108733	AR	NM_001683.4	ENSG00000157087
ATP6V0A2	12q24.31	611716	AR	NM_012463.3	ENSG00000185344
ATRX	Xq21.1	300032	XLD/XLR	NM_000489.4	ENSG00000085224
BBS4	15q24.1	600374	AR	NM_033028.4	ENSG00000140463
BCORL1	Xq26.1	300688	XLR	NM_021946.4	ENSG00000085185
BRCA2	13q13.1	600185	AD/AR	NM_000059.3	ENSG00000139618
BRWD3	Xq21.1	300553	XLR	NM_153252.4	ENSG00000165288
C12orf57	12p13.31	615140	AR	NM_138425.3	ENSG00000111678
CACNA1B	9q34.3	601012	AR	NM_000718.3	ENSG00000148408

CACNA1D	3p21.1	114206	AD/AR	NM_000720.3	ENSG00000157388
CACNA1F	Xp11.23	300110	XLR	NM_005183.3	ENSG00000102001
CAMK2A	5q32	114078	AD/AR	NM_015981.3	ENSG00000070808
CARD11	7p22.2	607210	AD/AR	NM_032415.5	ENSG00000198286
CC2D1A	19p13.12	610055	AR	NM_017721.4	ENSG00000132024
CCDC88C	14q32.11-q32.12	611204	AD/AR	NM_00108041	ENSG00000015133
CEP135	4q12	611423	AR	NM_025009.4	ENSG00000174799
CEP290	12q21.32	610142	AR	NM_025114.3	ENSG00000198707
CEP41	7q32.2	610523	AR	NM_018718.2	ENSG00000106477
CHKB	22q13.33	612395	AR	NM_005198.4	ENSG00000100288
CHMP1A	16q24.3	164010	AR	NM_002768.4	ENSG00000131165
CHRM3	1q43	118494	AR	NM_000740.3	ENSG00000133019
CIB2	15q25.1	605564	AR	NM_006383.3	ENSG00000136425
CLN8	8p23.3	607837	AR	NM_018941.3	ENSG00000182372
CNGB3	8q21.3	605080	AR	NM_019098.4	ENSG00000170289
CNTNAP2	7q35-q36	604569	AR	NM_014141.5	ENSG00000174469
CPT2	1p32.3	600650	AD/AR	NM_000098.2	ENSG00000157184
CORO1A	16p11.2	605000	AR	NM_007074.3	ENSG00000102879
CTNNA2	2p12	114025	AR	NM_00116488	ENSG00000066032
CUL7	6p21.1	609577	AR	NM_014780.4	ENSG00000044090
CYP11B1	8q24.3	610613	AD/AR	NM_000497.3	ENSG00000160882
CYP27A1	2q35	606530	AR	NM_000784.3	ENSG00000135929
DDX3X	Xp11.4	300160	XLD/XLR	NM_00119341	ENSG00000215301
DEAF1	11p15.5	602635	AD/AR	NM_021008.3	ENSG00000177030
DHCR7	11q13.4	602858	AR	NM_001360.2	ENSG00000172893
DDHD2	8p11.23	615003	AR	NM_015214.2	ENSG00000085788
DMD	Xp21.2-p21.1	300377	XLR	NM_004006.2	ENSG00000198947
DNAH17	17q25.3	610063	AR	NM_173628.4	ENSG00000187775
DOCK8	9p24.3	611432	AR	NM_203447.3	ENSG00000107099
DOLK	9q34.11	610746	AR	NM_014908.3	ENSG00000175283
DPYD	1p21.3	612779	AR	NM_000110.3	ENSG00000188641
DST	6p12.1	113810	AR	NM_001723.5	ENSG00000151914
DDC	7p12.2-p12.1	107930	AR	NM_000790.3	ENSG00000132437

DCC	18q21.2	120470	AD/AR	NM_005215.3	ENSG00000187323
EPHB2	1p36.12	600997	AR	NM_017449.4	ENSG00000133216
ESRRB	14q24.3	602167	AR	NM_004452.3	ENSG00000119715
ETFB	19q13.41	130410	AR	NM_001985.2	ENSG00000105379
EXOC6B	2p13.2	607880	AR	NM_015189.2	ENSG00000144036
FAN1	15q13.3	613534	AR	NM_014967.4	ENSG00000198690
FGA	4q31.3	134820	AD/AR	NM_021871.3	ENSG00000171560
ENPP1	6q23.2	173335	AD/AR	NM_006208.2	ENSG00000197594
GATM	15q21.1	602360	AD/AR	NM_001482.2	ENSG00000171766
GPC4	Xq26.2	300168	XLR	NM_001448.2	ENSG00000076716
GPC6	13q31.3-q32.1	604404	AR	NM_005708.4	ENSG00000183098
GPHN	14q23.3	603930	AR	NM_020806.4	ENSG00000171723
GPX1	3p21.31	138320	AR	NM_000581.3	ENSG00000233276
GRID2	4q22.1-q22.2	602368	AR	NM_001510.3	ENSG00000152208
GRIK2	6q16.3	138244	AR	NM_021956.4	ENSG00000164418
FXN	9q21.11	606829	AR	NM_000144.4	ENSG00000165060
GALNT2	1q42.13	602274	AR	NM_004481.4	ENSG00000143641
GRIN1	9q34.3	138249	AD/AR	NM_007327.3	ENSG00000176884
GRIP1	12q14.3	604597	AR	NM_021150.3	ENSG00000155974
GRM7	3p26.1	604101	AR	NM_000844.3	ENSG00000196277
HCFC1	Xq28	300019	XLR	NM_005334.2	ENSG00000172534
HEPACAM	11q24.2	611642	AD/AR	NM_152722.4	ENSG00000165478
HERC2	15q13.1	605837	AR	NM_004667.5	ENSG00000128731
HOXA1	7p15.2	142955	AR	NM_005522.4	ENSG00000105991
HYDIN	16q22.2	610812	AR	NM_00127097	ENSG00000157423
IL1RAPL1	Xp21.3-p21.2	300206	XLR	NM_014271.3	ENSG00000169306
INTS1	7p22.3	611345	AR	NM_00108045	ENSG00000164880
ITGB3	17q21.32	173470	AD/AR	NM_000212.2	ENSG00000259207
ITPR1	3p26.1	147265	AD/AR	NM_002222.5	ENSG00000150995
IGF1	12q23.2	147440	AR	NM_000618.4	ENSG00000017427
KCNJ10	1q23.2	602208	AR	NM_002241.4	ENSG00000177807
KCNMA1	10q22.3	600150	AD/AR	NM_002247.3	ENSG00000156113
KDM5B	1q32.1	605393	AR	NM_006618.4	ENSG00000117139

KDM5C	Xp11.22	314690	XLR	NM_004187.3	ENSG00000126012
KIF14	1q32.1	611279	AR	NM_014875.2	ENSG00000118193
KNG1	3q27.3	612358	AR	NM_00110241	ENSG00000113889
KPTN	19q13.32	615620	AR	NM_007059.3	ENSG00000118162
LAMA1	18p11.31	150320	AR	NM_005559.3	ENSG00000101680
LAMB1	7q31.1	150240	AR	NM_002291.2	ENSG00000091136
LAS1L	Xq12	300964	XLR	NM_031206.4	ENSG00000001497
LDLR	19p13.2	606945	AD/AR	NM_000527.4	ENSG00000130164
LEP	7q32.1	164160	AR	NM_000230.2	ENSG00000174697
LRBA	4q31.3	606453	AR	NM_006726.4	ENSG00000198589
LRP1	12q13.3	107770	AR	NM_002332.2	ENSG00000123384
LNPK	2q31.1	610236	AR	NM_030650.3	ENSG00000144320
LRP2	2q31.1	600073	AR	NM_004525.2	ENSG00000081479
LZTR1	22q11.21	600574	AD/AR	NM_006767.3	ENSG00000099949
MAOA	Xp11.3	309850	XLR	NM_000240.3	ENSG00000189221
MBOAT7	19q13.42	606048	AR	NM_00114608	ENSG00000125505
MCM4	8q11.21	602638	AR	NM_005914.3	ENSG00000104738
MCPH1	8p23.1	607117	AR	NM_024596.4	ENSG00000147316
MECP2	Xq28	300005	XLD/XLR	NM_004992.3	ENSG00000169057
MEGF10	5q23.2	612453	AR	NM_032446.2	ENSG00000145794
MET	7q31.2	164860	AD/AR	NM_00112750	ENSG00000105976
MFRP	11q23.3	606227	AR	NM_031433.3	ENSG00000235718
MTHFR	1p36.22	607093	AD/AR	NM_005957.4	ENSG00000177000
MYO1E	15q22.2	601479	AR	NM_004998.3	ENSG00000157483
MYO5A	15q21.2	160777	AR	NM_000259.3	ENSG00000197535
MYLK	3q21.1	600922	AD/AR	NM_053025.3	ENSG00000065534
NRXN1	2p16.3	600565	AR	NM_00113565	ENSG00000179915
NSMCE3	15q13.1	608243	AR	NM_138704.4	ENSG00000185115
NTNG2	9q34.13	618689	AR	NM_032536.4	ENSG00000196358
NTRK1	1q23.1	191315	AR	NM_00101233	ENSG00000198400
NUP133	1q42.13	607613	AR	NM_018230.3	ENSG00000069248
OCRL	Xq26.1	300535	XLR	NM_000276.3	ENSG00000122126
OFD1	Xp22.2	300170	XLD/XLR	NM_003611.2	ENSG00000046651

OPHN1	Xq12	300127	XLR	NM_002547.2	ENSG00000079482
NUP155	5p13.2	606694	AR	NM_153485.2	ENSG00000113569
PAH	12q23.2	612349	AR	NM_000277.2	ENSG00000171759
PCCA	13q32.3	232000	AR	NM_000282.3	ENSG00000175198
PCCB	3q22.3	232050	AR	NM_000532.4	ENSG00000114054
PCDH15	10q21.1	605514	AR/DR	NM_033056.3	ENSG00000150275
PEX7	6q23.3	601757	AR	NM_000288.3	ENSG00000112357
PHF8	Xp11.22	300560	XLR	NM_015107.2	ENSG00000172943
PLCB1	20p12.3	607120	AR	NM_015192.3	ENSG00000182621
POMGNT1	1p34.1	606822	AR	NM_017739.3	ENSG00000085998
POMT1	9q34.13	607423	AR	NM_007171.3	ENSG00000130714
PRICKLE1	12q12	608500	AR	NM_153026.2	ENSG00000139174
PRKDC	8q11.21	600899	AR	NM_006904.6	ENSG00000253729
PRKN	6q26	602544	AR	NM_004562.2	ENSG00000185345
PRODH	22q11.21	606810	AD/AR	NM_016335.4	ENSG00000100033
PTCHD1	Xp22.11	300828	XLR	NM_173495.2	ENSG00000165186
POLR3A	10q22.3	614258	AR	NM_007055.3	ENSG00000148606
PTPRC	1q31.3-q32.1	151460	AR	NM_002838.4	ENSG00000081237
PXDN	2p25.3	605158	AR	NM_012293.2	ENSG00000130508
RAB39B	Xq28	300774	XLR	NM_171998.3	ENSG00000155961
RPL10	Xq28	312173	XLR	NM_006013.4	ENSG00000147403
SASH1	6q24.3-q25.1	607955	AD/AR	NM_015278.4	ENSG00000111961
RIMS2	8q22.3	606630	AR	NM_00110011	ENSG00000176406
RSRC1	3q25.32	613352	AR	NM_016625.3	ENSG00000174891
SBF1	22q13.33	603560	AR	NM_002972.3	ENSG00000100241
RAD21	8q24.11	606462	AD/AR	NM_006265.2	ENSG00000164754
SCN4A	17q23.3	603967	AD/AR	NM_000334.4	ENSG00000007314
SCN9A	2q24.3	603415	AD/AR	NM_002977.3	ENSG00000169432
SCP2	1p32.3	184755	AR	NM_002979.4	ENSG00000116171
SERPINE1	7q22.1	173360	AD/AR	NM_000602.4	ENSG00000106366
SGSH	17q25.3	605270	AR	NM_000199.4	ENSG00000181523
SHOX	Xp22.33	312865	PD/PR	NM_000451.3	ENSG00000185960
SLC12A5	20q13.12	606726	AD/AR	NM_020708.4	ENSG00000124140

SLC1A1	9p24.2	133550	AR	NM_004170.5	ENSG00000106688
SLC25A12	2q31.1	603667	AR	NM_003705.4	ENSG00000115840
SLC45A1	1p36.23	605763	AR	NM_00108039	ENSG00000162426
SLC6A3	5p15.33	126455	AR	NM_001044.4	ENSG00000142319
SLC6A8	Xq28	300036	XLR	NM_005629.3	ENSG00000130821
SLC7A7	14q11.2	603593	AR	NM_00112610	ENSG00000155465
SLCO1B3	12p12.2	605495	DR	NM_019844.3	ENSG00000111700
SNX14	6q14.3	616105	AR	NM_153816.5	ENSG00000135317
SYN1	Xp11.3-p11.2	313440	XLD/XLR	NM_133499.2	ENSG00000008056
SYNE1	6q25.2	608441	AD/AR	NM_033071.3	ENSG00000131018
SYNJ1	21q22.11	604297	AR	NM_003895.3	ENSG00000159082
TAF1	Xq13.1	313650	XLR	NM_004606.4	ENSG00000147133
TAF6	7q22.1	602955	AR	NM_005641.3	ENSG00000106290
TBC1D23	3q12.1-q12.2	617687	AR	NM_00119919	ENSG00000036054
TBCK	4q24	616899	AR	NM_00116343	ENSG00000145348
SOD1	21q22.11	147450	AD/AR	NM_000454.4	ENSG00000142168
SYCE1	10q26.3	611486	AR	NM_130784.3	ENSG00000171772
TBX22	Xq21.1	300307	XLR	NM_00110987	ENSG00000122145
TECTA	11q23.3	602574	AD/AR	NM_005422.2	ENSG00000109927
TMLHE	Xq28	300777	XLR	NM_018196.3	ENSG00000185973
TRAPPC6B	14q21.1	610397	AR	NM_00107953	ENSG00000182400
TRAPPC9	8q24.3	611966	AR	NM_031466.7	ENSG00000167632
TET3	2p13.1	613555	AD/AR	NM_00128749	ENSG00000187605
TRAPPC2L	16q24.3	610970	AR	NM_016209.5	ENSG00000167515
TRIM32	9q33.1	602290	AR	NM_012210.3	ENSG00000119401
TSPAN7	Xp11.4	300096	XLR	NM_004615.3	ENSG00000156298
TTI2	8p12	614426	AR	NM_00110240	ENSG00000129696
TTN	2q31.2	188840	AD/AR	NM_133378.4	ENSG00000155657
UNC80	2q34	612636	AR	NM_032504.1	ENSG00000144406
UPF3B	Xq24	300298	XLR	NM_080632.2	ENSG00000125351
USH2A	1q41	608400	AR	NM_206933.2	ENSG00000042781
USP45	6q16.2	618439	AR	NM_00108048	ENSG00000123552
USP9X	Xp11.4	300072	XLD/XLR	NM_00103959	ENSG00000124486

VPS13B	8q22.2	607817	AR	NM_017890.4	ENSG00000132549
TDO2	4q32.1	191070	AR	NM_005651.3	ENSG00000151790
TPO	2p25.3	606765	AR	NM_000547.5	ENSG00000115705
TTC25	17q21.2	617095	AR	NM_031421.5	ENSG00000204815
VDR	12q13.11	601769	AR	NM_00101753	ENSG00000111424
WNT1	12q13.12	164820	AR	NM_005430.3	ENSG00000125084
WWOX	16q23.1-q23.2	605131	AR	NM_016373.3	ENSG00000186153
XPC	3p25.1	613208	AR	NM_004628.4	ENSG00000154767
UBR1	15q15.2	605981	AR	NM_174916.2	ENSG00000159459
ZBTB16	11q23.2	176797	AR	NM_006006.5	ENSG00000109906
ZFYVE26	14q24.1	612012	AR	NM_015346.3	ENSG00000072121

Reference: SFARI Gene. [(accessed on 29 October 2020)]; Available online: https://gene.sfari.org/.
OMIM-Oline Mendelian Inheritance In Man. Available online: https://omim.org/.
HGMD-The Human Gene Mutation Database. Available online: http://www.hgmd.cf.ac.uk/ac/i

Table S4. Human *in vitro* cell models of ASD.

Cell lines derived from hiPSCs	ASD associated gene	Cellular phenotype(s)	References
Cortical neurons	*EHMT1*	-Reduced neurite length and complexityAltered neuronal activity<br>-Increased expression of proliferation genes<br>-Decreased expression of maturation and migration genes	[1]
	MECP2	-Increased synaptogenesis and dendritic complexity<br>-Altered neuronal network synchronization	[2]
	NRXN1	-Altered ion transport and calcium signaling	[3]
	PTCHD1	-Decreased frequency of miniature excitatory postsynaptic currents<br>-N-methyl-D-aspartate receptor (NMDARs) hypofunction	[4]
	PTCHD1-AS	-Decreased frequency of miniature excitatory postsynaptic currents	[3]
	SHANK2	-Increased number of synapses, dendritic length and complexity<br>-Increased frequency of spontaneous excitatory postsynaptic currents<br>-Altered expression of genes associated to neuronal morphogenesis, plasticity and synapse	[5]
	SHANK3	-Synaptic alteration and decreased dendritic spines	[6-7]
	TSC2	-Mitochondria disorganization and altered mitophagy<br>-Increased soma size and neurite number<br>-mTORC1 signaling pathway hyperactivation<br>-Increased neuronal activity and upregulation of cell adhesion genes	[8-9]
Dopaminergic neurons	*RELN*	-Altered neuronal migration	[10]
	AFF2	-Alteration in genes associated with neuronal development<br>-Decreased synaptic activity: reduced spontaneous excitatory postsynaptic currents	[11]
	ASTN2	-Alteration in genes associated with neuronal development	[11]

Glutamatergic neurons		-Decreased synaptic activity: reduced spontaneous excitatory postsynaptic currents	
	ATRX	-Alteration in genes associated with neuronal development<br>-Decreased synaptic activity: reduced spontaneous excitatory postsynaptic currents	[11]
	CNTN5	-Increased neuronal activity	[12]
	KCNQ2	-Decreased synaptic activity: reduced spontaneous excitatory postsynaptic currents	[11]
	SCN2A	-Alteration in genes associated with morphogenesis<br>-Decreased synaptic activity: reduced spontaneous excitatory postsynaptic currents	[11]
Neuron-like cells	*ARHGEF9*	-Altered mTORC1 signaling pathway	[13]
	CACNA1C	-Altered calcium signaling<br>-Altered differentiation of neurons from cortical layers<br>-Increased production of norepinephrine and dopamine<br>-Altered expression of tyrosine hydrolase	[14-15]
	CDKL5	-Alterations in neuronal activity	[16]
	CHD8	-Altered expression of genes associated with neural development, -β-catenin/Wnt signaling, extracellular matrix and skeletal system development	[17]
	COSMOC	-Impaired redox homeostasis<br>-Altered *PTBP2* splicing	[18]
	FMR1	-Altered DNA methylation patterns<br>-Altered expression of genes associated with neuronal development, migration and maturation<br>-Altered neurite formation and neuronal differentiation	[19-21]
	SHANK3	-Alterations in the soma and neurites, as well as alterations in synaptic transmission<br>-Altered expression of genes associated to motility and neurogenesis	[22-23]
	TRPC6	-Reduce neurite length and complexity<br>-Altered glutamatergic synapse formation and reduced sodium influx	[24]
Neural	*CHD8*	-Alterations in the expression of gens associated with neurogenesis, -β-catenin/Wnt signaling, neuronal differentiation and axonal guidance	[25]
	FOXG1	-Upregulation of neural cell fate, axon guidance, synaptic and GABAergic genes, downregulation	[26]

organoids		of non-neuronal genes, decreased cell cycle length, increased MAP2 density<br>-Increased inhibitory synapse density<br>-Increased GABAergic progenitor and neuron production	
Neural progenitor cells	*NRXN1*	-Alterations in neuronal adhesion and differentiation<br>-Overactivation of mTORC1 pathway	[27-28]
	RELN	-Alterations in neuronal adhesion and differentiation<br>-Overactivation of mTORC1 pathway	[29]
	TRPC6	-Altered calcium signaling and expression of genes involved in cell adhesion and neurite formation	[24]
	ZNF804A	-Altered expression of pathways mediated by interferon-α 2	[30]
Olfactory placodal neurons	*SHANK3*	-Decreased number of synapses<br>-Alterations during neural development in the soma and neurites	[31]
Purkinje cells	*TSC2*	-Hypoexcitability and synaptic dysfunction<br>-mTORC1 pathway hyperactivation<br>-Altered neuronal differentiation	[32]

References

1. Nagy J, et al. (2017) Altered neurite morphology and cholinergic function of induced pluripotent stem cell-derived neurons from a patient with Kleefstra Syndrome and autism. Transl Psychiatry. 7:e1179. 10.1038/tp.2017.144.
2. Nageshappa S, et al. (2016) Altered neuronal network and rescue in a human MECP2 duplication model. Mol Psychiatry. 21(2):178–88. 10.1038/mp.2015.128.
3. Avazzadeh S, et al. (2019) Increased Ca2+ signaling in NRXN1α +/- neurons derived from ASD induced pluripotent stem cells. Mol Autism. 10:52. 10.1186/s13229-019-0303-3.
4. Ross PJ, et al. (2020) Synaptic dysfunction in human neurons with autism-associated deletions in PTCHD1-AS. Biol Psychiatry. 87(2):139–49. 10.1016/j.biopsych.2019.07.014.
5. Zaslavsky K., et al. (2019) SHANK2 mutations associated with autism spectrum disorder cause hyperconnectivity of human neurons. Nat Neurosci. 22(4):556–64. 10.1038/s41593-019-0365-8.
6. Gouder L, et al. (2019) Altered spinogenesis in iPSC-derived cortical neurons from patients with autism carrying de novo SHANK3 mutations. Sci Rep. 9:94. 10.1038/s41598-018-36993-x.
7. Darville H, et al. (2016) Human pluripotent stem cell-derived cortical neurons for high throughput medication screening in autism: A proof of concept study in SHANK3 haploinsufficiency syndrome. EBioMedicine. 9:293–305. 10.1016/j.ebiom.2016.05.032.
8. Ebrahimi-Fakhari D, et al. (2016) Impaired mitochondrial dynamics and mitophagy in neuronal models of tuberous sclerosis complex. Cell Rep. 17:1053–70. 10.1016/j.celrep.2016.09.054.
9. Winden KD, et al. (2019) Biallelic mutations in TSC2 lead to abnormalities associated with cortical tubers in human iPSC-derived neurons. J Neurosci. 39(47):9294–05.

10.1523/JNEUROSCI.0642-19.2019.
10. Arioka Y, et al. (2018) Single-cell trajectory analysis of human homogenous neurons carrying a rare RELN variant. Transl Psychiatry. 8:129. 10.1038/s41398-018-0177-8.
11. Deneault E, et al. (2018) Complete disruption of autism-susceptibility genes by gene editing predominantly reduces functional connectivity of isogenic human neurons. Stem Cell Rep. 11:1211 – 25. 10.1016/j.stemcr.2018.10.003.
12. Deneault E, et al. (2019) CNTN5−/+ or EHMT2−/+ human iPSC-derived neurons from individuals with autism develop hyperactive neuronal networks. Elife. 8:e40092. 10.7554/eLife.40092.
13. Machado COF, et al. (2016) Collybistin binds and inhibits mTORC1 signaling: A potential novel mechanism contributing to intellectual disability and autism. Eur J Hum Genet. 24:59 – 65. 10.1038/ejhg.2015.69.
14. Paşca SP, et al. (2011) Using iPSC-derived neurons to uncover cellular phenotypes associated with Timothy Syndrome. Nat Med. 179(12):1657 – 62. 10.1038/nm.2576.
15. Tian Y, et al. (2014) Alteration in basal and depolarization induced transcriptional network in iPSC derived neurons from Timothy Syndrome. Genome Med. 6:75. 10.1186/s13073-014-0075-5.
16. Gao Y, et al. (2020) Gene replacement ameliorates deficits in mouse and human models of cyclin-dependent kinase-like 5 disorder. Brain. 143:811 – 32. 10.1093/brain/awaa028.
17. Wang P, et al. (2015) CRISPR/Cas9-mediated heterozygous knockout of the autism gene CHD8 and characterization of its transcriptional networks in neurodevelopment. Mol Autism. 6:55. 10.1186/s13229-015-0048-6.
18. Rontani P, et al. (2020) Impaired expression of the COSMOC/MOCOS gene unit in ASD patient stem cells. Mol Psychiatry. 10.1038/s41380-020-0728-2.
19. Boland MJ, et al. (2017) Molecular analyses of neurogenic defects in a human pluripotent stem cell model of Fragile X Syndrome. Brain. 140:582 – 98. 10.1093/brain/aww357.
20. Doers ME, et al. (2014) iPSC-derived forebrain neurons from FXS individuals show defects in initial neurite outgrowth. Stem Cells Dev. 23:1777 – 87. 10.1089/scd.2014.0030.
21. Sheridan SD, et al. (2011) Epigenetic characterization of the FMR1 gene and aberrant neurodevelopment in human induced pluripotent stem cell models of Fragile X Syndrome. PLoS ONE. 6:e26203. 10.1371/journal.pone.0026203.
22. Huang G, et al. (2019) Uncovering the functional link between SHANK3 deletions and deficiency in neurodevelopment using iPSC-derived human neurons. Front Neuroanat. 13:23. 10.3389/fnana.2019.00023.
23. Shcheglovitov A, et al. (2013) SHANK3 and IGF1 restore synaptic deficits in neurons from 22q13 deletion syndrome patients. Nature. 503(7475):267 – 271. 10.1038/nature12618.
24. Griesi-Oliveira K, et al. (2015) Modeling non-syndromic autism and the impact of TRPC6 disruption in human neurons. Mol. Psychiatry. 20:1350 – 65. 10.1038/mp.2014.141.
25. Wang P, et al. (2017) CRISPR/Cas9-mediated heterozygous knockout of the autism gene CHD8 and characterization of its transcriptional networks in cerebral organoids derived from iPS cells. Mol Autism. 8:11. 10.1186/s13229-017-0124-1.
26. Mariani J, et al. (2015) FOXG1-dependent dysregulation of GABA-glutamate neuron differentiation in autism spectrum disorders. Cell 162(2):375 – 90. 10.1016/j.cell.2015.06.034.
27. Lam M, et al. (2019) Single cell analysis of autism patient with bi-allelic NRXN1-alpha deletion reveals skewed fate choice in neural progenitors and impaired neuronal functionality. Exp Cell Res. 383:111469. 10.1016/j.yexcr.2019.06.014.

28. Zeng L, et al. (2013) Functional impacts of NRXN1 knockdown on neurodevelopment in stem cell models. PLoS ONE. 8:e59685. 10.1371/journal.pone.0059685.
29. Sánchez-Sánchez SM, et al. (2018) Rare RELN variants affect Reelin-DAB1 signal transduction in Autism Spectrum Disorder. Hum Mutat. 39(10):1372 – 83. 10.1002/humu.23584.
30. Chen J, et al. (2015) ZNF804A transcriptional networks in differentiating neurons derived from induced pluripotent stem cells of human origin. PLoS ONE. 10:e0124597. 10.1371/journal.pone.0124597.
31. Kathuria A. (2018) Stem cell-derived neurons from autistic individuals with SHANK3 mutation show morphogenetic abnormalities during early development. Mol Psychiatry. 23:735 – 46. 10.1038/mp.2017.185.
32. Sundberg M, et al. (2018) Purkinje cells derived from TSC patients display hypoexcitability and synaptic deficits associated with reduced FMRP levels and reversed by rapamycin. Mol Psychiatry. 23(11):2167–83. 10.1038/s41380-018-0018-4.

Table S5. Animal models of ASD.

Animal model	ASD associated gene	Gene modification technique	Observed phenotype	References
	BCKDK	Spontaneous mutation	-Neuronal alterations<br>-Reduced protein phosphorylation<br>-Infertility<br>-Altered development	[1]
	CACNA1C	KO by ZFN	-Altered social behavior and reduced USVs<br>-Increased perseverative behaviors	[2,3]
	CNTNAP2	KO by ZFN	-Seizures<br>-Hyperactivity<br>-Altered audition and sleep routines	[4,5]
	CYFIP1	KO by CRISPR/Cas9	-Neuronal alterations<br>-Altered behavioral flexibility in learning tasks	[6]
	FMR1	KO by ZFN	-Increased repetitive behaviors and social alterations.<br>-Altered sensorimotor gating<br>-Memory difficulties<br>-Neuronal alterations<br>-Altered auditory responses	[7-9]
	MECP2	KO by ZFN	-High mortality<br>-Malocclusion<br>-Neuronal alterations<br>-Hypoactivity<br>-Altered social interaction and speech responses.<br>-Memory alterations<br>-Decreased grip strength	[10-11]
	NLGN2	Overexpression in the hippocampus	-Decreased response to new stimuli and aggressive behavior	[12]
	NLGN3	KO by ZFN	-Increased repetitive	[8,13]

Mouse or Rat			behaviors<br>-Hyperactivity and altered sleep routines<br>-Decreased body weight<br>-Altered juvenile play behavior and startle response<br>-Altered sensorimotor gating	
	NLGN4	KO	-Aggression behavior<br>-Altered social communication<br>-Decreased brain volume	[14]
	NRXN1	KO by biallelic deletion	-Hyperactivity<br>-Altered startle response<br>-Memory alterations	[15]
	PTEN	Heterozygous KO by ZFN	-Neuronal alterations	[16]
	SCN1A	KO by ENU mutagenesis	-Increased repetitive behaviors<br>-Hyperactivity and anxiety<br>-Learning and memory difficulties<br>-Motor alterations<br>-Reduced dopamine levels	[17]
	SHANK2	KO by ZFN	-Alterations in social behavior<br>-Hyperactivity and increased repetitive behavior<br>-Memory alterations<br>-Neuronal alterations	[18]
	SHANK3	KO by ZFN	-Alterations in social behavior<br>-Neuronal alterations	[19]
	TBR1	Heterozygous KO	-Alterations in social behavior<br>-Learning deficits<br>-Conditioned taste aversion defects	[20,21]

			-Axonal projection defects in amygdala<br>-Decreased NMDAR function	
	TCF4	KO by CRISPR/Cas9 and KD by shRNA in the prefrontal cortex	-Altered electrophysiological properties in neurons	[22]
	TSC1	Heterozygous KO/ KO	-Alterations in social behavior<br>-Ataxia<br>-Cerebellar deficits	[23]
	TSC2	Spontaneous mutation	-Enhanced episodic-like memory<br>-Enhanced seizure-induced plasticity<br>-Increased induction of phospho-p42-MAPK in the hippocampus<br>-Increased basal oxygen consumption in the brain	[24,25]
	UBE3A	KO by CRISPR/Cas9	-Motor, learning and memory difficulties	[26]
	ARID1B	KD by MOs	-Reduced body length<br>-Altered expression of chondrogenic/osteogenic genes	[27]
	ARX	KD by MOs	-Altered brain development<br>-Neuronal alterations	[28]
	AUTS2	KD by MOs	-Microcephaly<br>-Altered jaw development<br>-Motor alterations<br>-Neuronal alterations	[29]
	CACNA1C	KD by MOs	-Cardiac alterations<br>-Altered jaw development	[30]
	CEP41	KD by MOs	-Neuronal alterations<br>-Social behavior alterations	[31]
	CHD2	KD by MOs	-Altered development<br>-Microcephaly, abnormal body curvature<br>-Swim bladder absence<br>Motor difficulties	[32]
	CHD8	KO by CRISPR/Cas9	-Macrocephaly	[33,34]

Zebrafish		and KD by MOs	-Reduction in post-mitotic enteric neurons	
	CNTNAP2	KO by ZFN	-Altered development<br>-Microcephaly<br>-Neuronal alterations<br>-Motor alterations	[35]
	CTNND2	KD by MOs	-Reduced body length<br>-Notochord alterations	[36]
	DYRK1A	KO by TALENs	-Altered response to social stimuli	[37]
	FMR1	KO by ENU-mutagenesis and CRISPR/Cas9	-Altered cephalic development<br>-Hyperactivity<br>-Increased anxiety<br>-Altered social behavior<br>-Learning difficulties	[38-40]
	KCNJ10	KD by MOs	-Motor alterations<br>-Altered development	[41]
	KDM6A	KD by MOs	-Reduced body length<br>-Altered development<br>-Notochord alterations<br>-Neuronal alterations	[42,43]
	MECP2	KO by ENU-mutagenesis and KD by MOs	-Altered immune response<br>-Neuronal alterations	[44-46]
	MET	KD by MOs	-High mortality<br>-Neuronal alterations	[47]
	MYT1L	KD by MOs	-Reduced levels of oxytocin	[48]
	NBEA	KO by ENU-mutagenesis and TALENs	-Neuronal alterations<br>-Altered response to startle stimuli	[49]
	NR3C2	KO by CRISPR/Cas9	-Altered social behavior<br>-Altered sleep routines	[50]
	OXTR	KO by TALENs	-Altered oxytocin signaling pathway<br>-Memory alterations in social and non-social recognition	[51]
	RELN	KO by TALENs	-Altered social behavior<br>-Altered serotonin signaling pathway	[52]
	RERE	KO by ENU-mutagenesis	-Altered startle response to stimuli	[53]

			-Vision and hearing difficulties	
	SHANK3	KO by CRISPR/Cas9	-Altered development<br>-Neuronal alterations<br>-Reduced social behavior,<br>-hypoactivity	[54,55]
	SYNGAP1	KD by MOs	-Delayed development<br>-High mortality<br>-Neuronal alterations<br>-Motor difficulties	[54]
Monkey or Dog	*MECP2*	TALEN mediated mutation/Overexpression	-Altered social behavior<br>-Defects in recognizing emotional expressions<br>-Altered cortical and sub-cortical volume	[56,57]
	SHANK3	CRISPR/Cas9 mediated mutation	-Fewer neurons and reduced spine density	[58]

CRISPR/Cas9: clustered regularly interspersed short palindromic repeats/CRISPR-associated protein 9; ENU: N-ethyl-N-nitrosourea; KD: knockdown; KO: knockout; TALEN: transcription activator-like effector nuclease; MO: morpholino phosphorodiamidate antisense oligonucleotides; ZFN: zinc-finger nuclease.

References

1. Zigler JS, et al. (2016) A Spontaneous missense mutation in branched chain keto acid dehydrogenase kinase in the rat affects both the central and peripheral nervous systems. PLoS ONE. 11:e0160447. 10.1371/journal.pone.0160447.
2. Kisko TM, et al. (2018) Cacna1c haploinsufficiency leads to pro-social 50-kHz ultrasonic communication deficits in rats. Dis Model Mech. 11(6):dmm034116. 10.1242/dmm.034116.
3. Wöhr M, et al. (2020) Sex-dependent effects of Cacna1c haploinsufficiency on behavioral inhibition evoked by conspecific alarm signals in rats. Prog Neuro-Psychopharmacol Biol Psychiatry. 99:109849. 10.1016/j.pnpbp.2019.109849.
4. Scott KE, et al. (2018) Altered auditory processing, filtering, and reactivity in the Cntnap2 knock-out rat model for neurodevelopmental disorders. J Neurosci. 38(40):8588 – 604. 10.1523/JNEUROSCI.0759-18.2018.
5. Thomas AM, et al. (2017) Cntnap2 KO rats and mice exhibit epileptiform activity and abnormal sleep – wake physiology. Sleep. 40(1). 10.1093/sleep/zsw026.
6. Silva AI, et al. (2019) Cyfip1 haploinsufficient rats show white matter changes, myelin thinning, abnormal oligodendrocytes and behavioural inflexibility. Nat Commun. 10:3455. 10.1038/s41467-019-11119-7.
7. Asiminas A, et al. (2019) Sustained correction of associative learning deficits after brief, early treatment in a rat model of Fragile X Syndrome. Sci Transl Med. 11(494):eaao0498. 10.1126/scitranslmed.aao0498.
8. Hamilton SM, et al. (2014) Fmr1 and Nlgn3 KO rats: Novel tools for investigating autism spectrum disorders. Behav Neurosci. 128(2):103 – 9. 10.1037/a0035988.
9. Ruby K., et al. (2015) Abnormal neuronal morphology and neurochemistry in the auditory

brainstem of Fmr1 KO rats. Neuroscience. 303:285 ‑ 98. 10.1016/j.neuroscience.2015.06.061.
10. Engineer CT, et al. (2015) Degraded neural and behavioral processing of speech sounds in a rat model of Rett syndrome. Neurobiol Dis. 83:26 ‑ 34. 10.1016/j.nbd.2015.08.019.
11. Wu Y, et al. (2016) Characterization of Rett Syndrome-like phenotypes in Mecp2-KO rats. J Neurodev Disord. 8:23. 10.1186/s11689-016-9156-7.
12. Kohl C, et al. (2013) Hippocampal neuroligin-2 overexpression leads to reduced aggression and inhibited novelty reactivity in rats. PLoS ONE. 8:e56871. 10.1371/journal.pone.0056871.
13. Thomas AM, et al. (2017) Sleep/wake physiology and quantitative electroencephalogram analysis of the Neuroligin-3 KO rat model of Autism Spectrum Disorder. Sleep. 40(10). 10.1093/sleep/zsx138.
14. Jamain S, et al. (2008) Reduced social interaction and ultrasonic communication in a mouse model of monogenic heritable autism. Proc Natl Acad Sci USA. 105(5):1710 ‑ 5. 10.1073/pnas.0711555105.
15. Esclassan F., et al. (2015) Phenotypic characterization of nonsocial behavioral impairment in neurexin 1α KO rats. Behav Neurosci. 129(1):74 ‑ 85. 10.1037/bne0000024.
16. Rowley PA, et al. (2019) Convergent microstructural brain changes across genetic models of Autism Spectrum Disorder—A pilot study. Psychiatry Res Neuroimaging. 283:83 ‑ 91. 10.1016/j.pscychresns.2018.12.007.
17. Ohmori I, et al. (2014) Methylphenidate improves learning impairments and hyperthermia-induced seizures caused by an Scn1a mutation. Epilepsia. 55(10):1558 ‑ 67. 10.1111/epi.12750.
18. Modi ME, et al. (2018) Hyperactivity and hypermotivation associated with increased striatal mGluR1 signaling in a Shank2 rat model of autism. Front. Mol Neurosci. 11:107. 10.3389/fnmol.2018.00107.
19. Harony-Nicolas H, et al. (2017) Oxytocin improves behavioral and electrophysiological deficits in a novel Shank3-deficient rat. Elife. 6:e18904. 10.7554/eLife.18904.
20. Huang TN, et al. (2014) Tbr1 haploinsufficiency impairs amygdalar axonal projections and results in cognitive abnormality. Nat Neurosci. 17(2): 240 ‑ 7. 10.1038/nn.3626.
21. Lee EJ, et al. (2015) Trans-synaptic zinc mobilization improves social interaction in two mouse models of autism through NMDAR activation. Nat Commun. 6: 7168. 10.1038/ncomms8168.
22. Rannals MD, et al. (2016) Neurodevelopmental models of transcription factor 4 deficiency converge on a common ion channel as a potential therapeutic target for Pitt Hopkins Syndrome. Rare Dis. 4:e1220468. 10.1080/21675511.2016.1220468.
23. Tsai PT, et al. (2012) Autistic-like behavior and cerebellar dysfunction in Purkinje cell Tsc1-mutant mice. Nature. 488(7431): 647 ‑ 51. 10.1038/nature11310.
24. Chi OZ, et al. (2015) Restoration of normal cerebral oxygen consumption with rapamycin treatment in a rat model of autism ‑ tuberous sclerosis. NeuroMol Med. 17(3):305 ‑ 13. 10.1007/s12017-015-8359-5.
25. Waltereit R, et al. (2006) Enhanced episodic-like memory and kindling epilepsy in a rat model of tuberous sclerosis. J Neurochem. 96(2):407 ‑ 13. 10.1111/j.1471-4159.2005.03538.x.
26. Dodge A, et al. (2020) Generation of a novel rat model of Angelman Syndrome with a complete Ube3a gene deletion. Autism Res. 13(3):397 ‑ 409. 10.1002/aur.2267.
27. Liu X, et al. (2020) De Novo ARID1B mutations cause growth delay associated with aberrant Wnt/β ‑ catenin signaling. Hum Mutat. 41(5):1012 ‑ 24. 10.1002/humu.23990.
28. Ishibashi M, et al. (2015) Copy number variants in patients with intellectual disability affect the regulation of ARX transcription factor gene. Hum Genet. 134(11-12):1163 ‑ 82. 10.1007/s00439-015-1594-x.

29. Oksenberg N, et al. (2013) Function and regulation of AUTS2, a gene implicated in autism and human evolution. PLoS Genet. 9:e1003221. 10.1371/journal.pgen.1003221.
30. Ramachandran KV, et al. (2013) Calcium influx through L-type CaV1.2 Ca2+ channels regulates mandibular development. J Clin Invest. 123(4):1638–46. 10.1172/JCI66903.
31. Patowary A, et al. (2019) Family-based exome sequencing and case-control analysis implicate CEP41 as an ASD gene. Transl Psychiatry. 9(1):4. 10.1038/s41398-018-0343-z.
32. Suls A, et al. (2013) De novo loss-of-function mutations in CHD2 cause a fever-sensitive myoclonic epileptic encephalopathy sharing features with dravet syndrome. Am J Hum Genet 93(5):967–75. 10.1016/j.ajhg.2013.09.017.
33. Bernier R, et al. (2014) Disruptive CHD8 mutations define a subtype of autism early in development. Cell. 158(2):263–76. 10.1016/j.cell.2014.06.017.
34. Sugathan A, et al. (2014) CHD8 regulates neurodevelopmental pathways associated with autism spectrum disorder in neural progenitors. Proc Natl Acad Sci USA. 111:E4468–E4477. 10.1073/pnas.1405266111.
35. Hoffman EJ, et al. (2016) Estrogens suppress a behavioral phenotype in zebrafish mutants of the autism risk gene, CNTNAP2. Neuron. 89(4):725–33. 10.1016/j.neuron.2015.12.039.
36. Turner TN, et al. (2015) Loss of δ-catenin function in severe autism. Nature. 520(7545):51–6. 10.1038/nature14186.
37. Kim OH, et al. (2017) Zebrafish KO of Down syndrome gene, DYRK1A, shows social impairments relevant to autism. Mol Autism. 8:50. 10.1186/s13229-017-0168-2.
38. Hu J, et al. (2020) Hyperactivity, memory defects, and craniofacial abnormalities in zebrafish fmr1 mutant larvae. Behav Genet. 50(3):152–60. 10.1007/s10519-020-09995-7.
39. Kim L, et al. (2014) Anxiety, hyperactivity and stereotypy in a zebrafish model of Fragile X Syndrome and Autism Spectrum Disorder. Prog Neuro-Psychopharmacol Biol Psychiatry. 55:40–9. 10.1016/j.pnpbp.2014.03.007.
40. Wu YJ, et al. (2017) Fragile X mental retardation-1 KO zebrafish shows precocious development in social behavior. Zebrafish. 14(5):438–43. 10.1089/zeb.2017.1446.
41. Sicca F, et al. (2016) Gain-of-function defects of astrocytic Kir4.1 channels in children with Autism Spectrum Disorders and epilepsy. Sci Rep. 6:34325. 10.1038/srep34325.
42. Bögershausen N, et al. (2015) RAP1-mediated MEK/ERK pathway defects in Kabuki syndrome. J Clin Invest. 125(9):3585–99. 10.1172/JCI80102.
43. Van Laarhoven P.M, et al. (2015) Kabuki syndrome genes KMT2D and KDM6A: Functional analyses demonstrate critical roles in craniofacial, heart and brain development. Hum Mol Genet. 24(15):4443–53. 10.1093/hmg/ddv180.
44. Leong WY, et al. (2015) Methyl-CpG binding protein 2 (Mecp2) regulates sensory function through Sema5b and Robo2. Front Cell Neurosci. 9:481. 10.3389/fncel.2015.00481.
45. Pietri T, et al. (2013) The first mecp2-null zebrafish model shows altered motor behaviors. Front Neural Circuits. 7:118. 10.3389/fncir.2013.00118.
46. Van Der Vaart M, et al. (2017) Mecp2 regulates tnfa during zebrafish embryonic development and acute inflammation. DMM Dis. Model Mech. 10(12):1439–51. 10.1242/dmm.026922.
47. Elsen GE, et al. (2009) The autism susceptibility gene met regulates zebrafish cerebellar development and facial motor neuron migration. Dev Biol. 335(1):78–92. 10.1016/j.ydbio.2009.08.024.
48. Blanchet P, et al. (2017) MYT1L mutations cause intellectual disability and variable obesity by

dysregulating gene expression and development of the neuroendocrine hypothalamus. PLoS. d Genet. 13(8):e1006957. 10.1371/journal.pgen.1006957.
49. Miller AC, et al. (2015) Neurobeachin is required postsynaptically for electrical and chemical synapse formation. Curr Biol. 25(1):16 – 28. 10.1016/j.cub.2014.10.071.
50. Ruzzo EK, et al. (2019) Inherited and de novo genetic risk for autism impacts shared networks. Cell. 178(4):850 – 866.e26. 10.1016/j.cell.2019.07.015.
51. Ribeiro D, et al. (2020) Oxytocin receptor signalling modulates novelty recognition but not social preference in zebrafish. J Neuroendocrinol. 32:e12834. 10.1111/jne.12834.
52. Vecchia ED, et al. (2019) Reelin signaling controls the preference for social novelty in zebrafish. Front Behav Neurosci. 13:214. 10.3389/fnbeh.2019.00214.
53. Plaster N, et al. (2007) REREa/Atrophin-2 interacts with histone deacetylase and Fgf8 signaling to regulate multiple processes of zebrafish development. Dev Dyn. 236(7):1891 – 904. 10.1002/dvdy.21196.
54. Kozol RA, et al. (2015) Two KD models of the autism genes SYNGAP1 and SHANK3 in zebrafish produce similar behavioral phenotypes associated with embryonic disruptions of brain morphogenesis. Hum Mol Genet. 24(14):4006 – 23. 10.1093/hmg/ddv138.
55. Liu CX, et al. (2018) CRISPR/Cas9-induced shank3b mutant zebrafish display autism-like behaviors. Mol Autism. 9:23. 10.1186/s13229-018-0204-x.
56. Chen Y, et al. (2017) Modeling Rett Syndrome Using TALEN-Edited MECP2 Mutant Cynomolgus Monkeys. Cell. 169(5):945-55.e10. 10.1016/j.cell.2017.04.035.
57. Liu Z, et al. (2016) Autism-like behaviours and germline transmission in transgenic monkeys overexpressing MeCP2. Nature. 530(7588):98-102. 10.1038/nature16533.
58. Zhao H, et al. (2018) Modeling autism in non-human primates: Opportunities and challenges. Autism Res. 11(5):686-94. 10.1002/aur.1945.
